# The intersection of the social determinants of health and antimicrobial resistance in human populations: a systematic review

**DOI:** 10.1136/bmjgh-2024-017389

**Published:** 2025-05-30

**Authors:** Alison E. Shutt, Diane Ashiru-Oredope, James Price, Maria Clara Padoveze, Nusrat Shafiq, Emma Carter, Amrita Ghataure, Sameed Shariq, Alison H Holmes, Esmita Charani

**Affiliations:** 1 Infectious Diseases, Faculty of Medicine, Imperial College London, London, UK; 2 UKHSA, London, UK; 3 Brighton and Sussex Medical School, Brighton, UK; 4 Escola de Enfermagem, University of Sao Paulo, Sao Paulo, Brazil; 5 Pharmacology, Post Graduate Institute of Medical Education and Research, Chandigarh, India; 6 Global Health and Infectious Diseases, Faculty of Health and Life Sciences, University of Liverpool, Liverpool, UK; 7 Division of Infectious Diseases and HIV Medicine, Groote Schuur Hospital, University of Cape Town, Cape Town, South Africa; 8 Faculty of Health and Life Sciences, Division of Pharmacology and Therapeutics, University of Liverpool, Liverpool, UK

**Keywords:** Global Health, Infections, diseases, disorders, injuries, Systematic review, Public Health, Health Services Accessibility

## Abstract

**Introduction:**

The social determinants of health (SDoH) impact the emergence and spread of antimicrobial resistance (AMR). We conducted a systematic review of literature mapping evidence on the intersection of SDoH, drug-resistant infections, antibiotic use, and public-facing interventions.

**Methods:**

Following Preferred Reporting Items for Systematic Reviews and Meta-Analyses guidelines, Ovid MEDLINE, Ovid EMBASE, the Cochrane Library, PsycINFO and Scopus were searched for published evidence in English between 2000 and 2022. Covidence software was used for data extraction. The evidence was mapped to the SDoH frameworks and the systematic review objectives.

**Results:**

Of 17 252 retrieved papers, 16 636 were included in title and abstract review, with 153 included in data extraction (126 empirical studies and 27 papers with secondary data). Of these, 92/126 (73%) were quantitative, 23/126 (18%) qualitative and 11/126 (9 %) mixed methods. There was evidence from high income 60/126 (47%), lower-middle income 41/126 (33%), low income 14 (11%) and upper-middle income 11 (9%) countries.

There is limited evidence associated with the complete range of SDoH in different populations. Reported determinants affecting the risk of exposure to and spread of AMR include (a) socioeconomic status and the associated impact on health seeking behaviours, (b) housing—living in a deprived area and overcrowding and (c) knowledge linked with education affecting practices, exacerbated by interconnected complexity with the associated influence of socioeconomic status. A gap in understanding the upstream systems which create inequality and negatively impact SDoH is evident. Numerous definitions are used to identify vulnerable populations. There is very limited research examining specific population groups, for example, traveller communities and the disabled. Reported interventions focus on awareness programmes with little evidence on sustained behaviour change.

**Conclusions:**

This review identified the need to (a) develop policies and context-specific solutions to manage upstream determinants, (b) include population groups where current evidence is limited and (c) prioritise community-based research using co-production methods.

**WHAT IS ALREADY KNOWN ON THIS TOPIC**

**WHAT THIS STUDY ADDS**

- This systematic review highlights the evidence gap in the intersection of SDoH and AMR, particularly across the full range of SDoH at macro (societal), meso (community / institutional) and micro (individual) levels. Evidence on the intersection of SDoH and AMR in deprived / vulnerable populations is limited with gaps in disabled, homeless, displaced populations, and traveller communities.
- Robust data are required across multiple indicators to fully assess and prioritise the SDoH. There is an opportunity to develop a framework for SDoH linked to AMR that enables the recognition of drivers and risks in different contexts.

**HOW THIS STUDY MIGHT AFFECT RESEARCH, PRACTICE OR POLICY**

- Recommendations are provided across macro levels, meso levels and micro levels. At macro level, policy recommendations include the importance of identifying and addressing the structural drivers which affect the SDoH.
- At a meso level and micro level, greater use of co-production methods is required to collaborate with individuals and communities to examine which determinants are causing people the most challenges. Sustained community engagement will enable better understanding of structural and behavioural changes required to manage AMR in diverse populations.

## Introduction

 Bacterial antimicrobial resistance (AMR) is a global health challenge with the greatest burden in populations in low-income (LIC) and lower-middle-income countries (LMICs) where inequity in access to resources across populations impacts healthcare access and outcomes.^1 2^ Increasingly, there is evidence of rising inequity in high-income countries (HICs), for example, the UK^3^ and the USA.^4^ The Indices of Deprivation highlight the uneven burden of deprivation across England, with areas in the North of England being severely affected for over a decade.^5^ Despite country-specific National Action Plans (NAPs) for AMR and widespread antimicrobial stewardship and infection prevention and control interventions, many challenges to mitigating this threat remain.^6^,^9^

The emergence and spread of AMR in human populations can be attributed to many factors including exposure to antibiotics, environments that foster transmission and acquisition of infections,^10^ lack of adequate access to water, sanitation and hygiene (WASH), infection prevention and vaccination, and socioeconomic and behavioural factors.^11^ Socioeconomic status, cultural norms, gender and power dynamics influence access to healthcare, healthcare provision and health outcomes.^12^,^15^ Social determinants of health (SDoH) as defined theoretically by Dahlgren and Whitehead^16^ are non-medical factors which affect everyone from birth to death impacting health equity and health outcomes.^17^ SDoH include income, education, unemployment, working life conditions, food insecurity, housing and the associated environment, early childhood development, social inclusion, access to health services.^17^ Social phenomena can be analysed across three distinct levels: macro society level, meso community level and micro individual level.^18^ The definitions by level are not fixed and can vary with context, for example, when considering health systems.^19^ SDoH transect the macro levels, meso levels and micro levels and are affected by respective power dynamics impacting populations to a greater or lesser extent. Marmot argues that approximately 20% of a person’s health is due to the healthcare they receive, while 80% is due to the SDoH.^20^ Health follows a social gradient with marginalised and deprived populations being affected by both health inequalities and inequities.^20 21^ Although studies related to AMR have found that a lower socioeconomic status corresponds with higher risks of infection and colonisation,^1122^,^24^ there is a lack of robust data across the complete range of SDoH.^25^ From a social science perspective, behaviours associated with antibiotic prescribing and (mis) use are linked with the emergence and spread of AMR.^26^,^28^

Previous systematic reviews have examined the relationship between different SDoH indicators/proxies and AMR, including poverty^29^; education as a SDoH and outlined in knowledge, attitude and practice (KAP) studies^30^; healthcare risk factors^31^; examination of peoples’ antibiotic use behaviours^32^,^36^; conflict, refugee and migrant status^37 38^; socioeconomic factors^39^ and prevalence and risk of methicillin-resistant *Staphylococcus aureus* (MRSA) in rural, remote and indigenous communities.^40^ These reviews broadly aimed to identify populations at greater risk of AMR and the conditions which increased vulnerability to AMR.

Defining vulnerability in relation to AMR is challenging as the terminology varies according to acceptable and contextualised socioeconomic indices for different countries and regions linked to their economic development profiles.^2041^,^43^ Within any country, there will be populations who experience socioeconomic deprivation and related health vulnerabilities. Different factors, including the socioeconomic status of people, determine whether individuals are defined as marginalised and deprived. Definitions for people in these groups include vulnerable, ‘hard to reach’, inclusion and disadvantaged health groups. Recent recommendations have suggested that there are no ‘hard to reach groups’ and the terminology should be replaced with under-represented or underserved.^44^,^46^ Despite the wide range of definitions, there is clear evidence that people in these groups are affected by health inequities which affect their health outcomes.^47^ Following the COVID-19 pandemic, there has been an increased focus on health inequalities in different populations.^48 49^ In the UK, this focus has increased recommendations and frameworks from both the National Health Service and the National Institute of Health Research^45 50^ for inclusion of populations affected by health inequalities.

We conducted this systematic review to (a) characterise the evidence on the intersection of the SDoH with AMR in human populations and (b) to identify interventions that are codesigned with populations considered at risk of AMR.

## Methods

### Search strategy and selection criteria

The Dahlgren and Whitehead model encompassing the SDoH at macro levels, meso levels and micro levels was used to theoretically underpin the review.^16 51^ The search strategy used the population, concept, context framework and was designed in four domains, namely (a) the SDoH, (b) health seeking behaviour and health provision, (c) the study population and (d) infections as outlined in the inclusion criteria combined with terms associated with AMR.^52^

The terms for each domain were developed and defined by (a) reviewing the Cochrane library for reviews, (b) search templates from other systematic reviews,^53 54^ (c) reviewing medical subject headings, (d) use of wild cards and (e) search terms were exploded and Boolean operators were used.

The Ovid MEDLINE platform, Ovid EMBASE, the Cochrane Library, PsycINFO and Scopus were used for the searches. Grey literature searches were conducted via Scopus and through hand searches. The full search strategy and terms are provided in the Online supplemental file. This study is registered with the International Prospective Register of Systematic Reviews (PROSPERO), registration number CRD42022337890. ^52^ The Preferred Reporting Items for Systematic Reviews and Meta-Analyses guidelines were used for the development of the study protocol, the systematic review analysis and the writing of the findings.^55^

### Patient and public involvement

As this was a systematic review, there was no public involvement in the conceptualisation or undertaking of this study.

### Inclusion and exclusion criteria

Published studies in English from 1 January 2000 to 31 December 2022 were eligible for inclusion. While we acknowledge that there was an ongoing trajectory of work associated with the SDoH prior to the year 2000 there were several key events from the year 2000 which impacted policy. These included: (a) The Millennium Development Goals were launched in September 2000, (b) The WHO launched the Commission for the SDoH in March 2005 and (c) The UK government requested Sir Michael Marmot to undertake an independent evidence-based review to reduce health inequalities in 2009. The end date was set by the end of the complete year closest to the systematic review being commenced. Further eligibility criteria included adults and children from low socioeconomic backgrounds, all ethnicities, protected characteristic groups as defined in the Equality and Human Rights Commission,^56^ the homeless, people with multimorbidities, migrants, asylum seekers, Gypsy Roma and traveller communities and studies undertaken in all country income categories as defined by The World Bank.^57^ Studies conducted with populations such as sex workers and people with drug and alcohol dependence were excluded as they are specifically defined specialist groups, with research conducted in these prescribed populations. We included the most common infections associated with drug resistance in human populations, namely skin and soft tissue infections, surgical wound infections, urinary tract infections (UTI), sepsis, bacteraemia and respiratory infections. Search terms also included the key pathogens associated with these infections. Excluded health conditions included participants with tuberculosis and/or human immunodeficiency virus, chronic obstructive pulmonary disease, and all other infections not stated in the inclusion criteria. Sexually transmitted infections (STIs) were excluded as we had excluded sex workers as this is a specialist field within literature.

### Data selection process

Covidence systematic review software was used to manage the search results, title and abstract review and data extraction. Duplicate references were automatically removed by the software system. Title and abstract screening, and full-text review were undertaken by five researchers. Conflicts were discussed in detail, and disagreements discussed through conflict review meetings. The inter-rater reliability Cohen’s kappa score was greater than 0.75 between all reviewers (scores are in the online supplemental material).

### Data extraction

Eligible papers had the following data extracted: author(s), title, year of publication, journal/document, country and urban/rural location, health setting (primary care, community, secondary or tertiary hospital), aims and objectives of the study, study inclusion and exclusion criteria, study design, quantitative, qualitative, mixed methods, research participants demographic and socioeconomic status where available, recruitment method, stated protected characteristics, stated inequalities, stated determinants of health, use of AMR language, stated infection, key findings, statistical analysis where applicable, thematic synthesis where applicable, quality assessment and risk of bias assessment.

### Risk of bias (quality) assessment

The extraction template was designed with a range of quality and risk assessment tools applicable for the study design.^58 59^ These included the Cochrane Risk assessment tool for bias ROB 1 and ROB 2.^59^,^61^

### Strategy for data analysis

Data were analysed in two phases. In phase,1 the methodological approach was examined, and the papers were categorised by primary data, secondary data, reviews and grey literature. In phase,2 the included studies with empirical data were descriptively analysed for evidence of (1) the influence of SDoH as defined by Dahlgren and Whitehead^16^ and the WHO,^17^ (2) the burden and impact of AMR across different populations disaggregated by the socioeconomic and demographic variables, for example, race, ethnicity, social deprivation, gender and (3) AMR-related interventions targeting marginalised/vulnerable populations and included co-production and public participation interventions with patients, public and communities.

## Results

**Figure 1:**
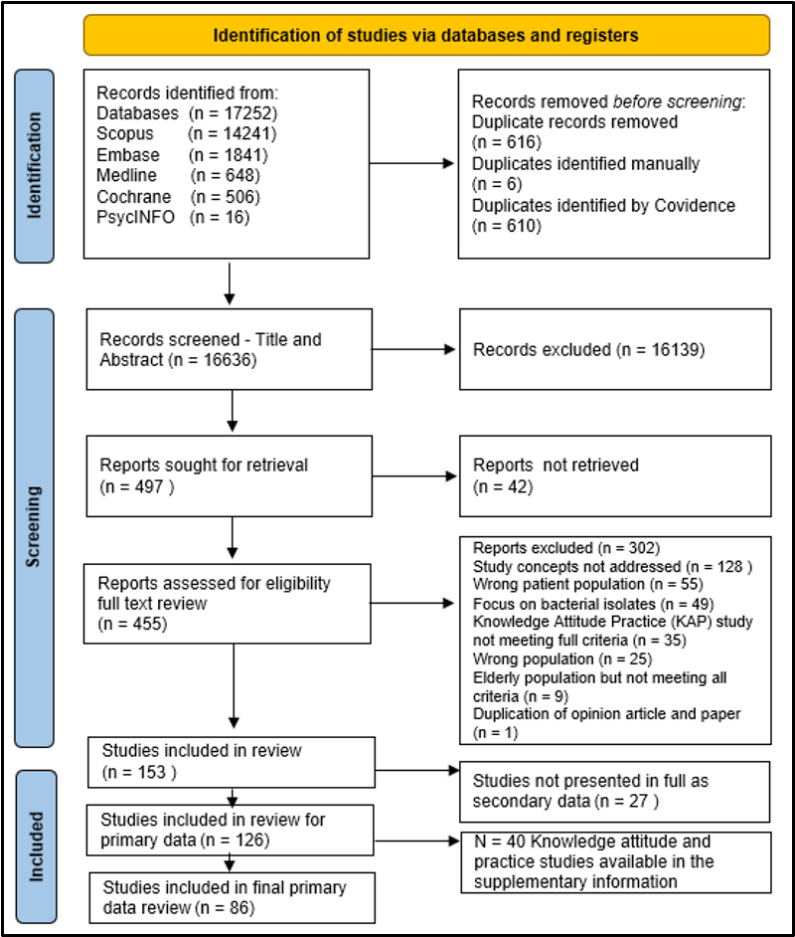
Study selection flow diagram for the systematic review on the intersection of the social determinants of health and antibiotic resistance in human populations.

Our initial search retrieved 17 252 papers, of which 616 were duplicates. The title and abstract reviews were conducted on 16 636 papers (figure 1). There were 153 papers included in the data extraction, with 126 having primary data and 27 secondary data. The study designs of primary papers were 92/126 (73%) quantitative, 23/126 (18%) qualitative and mixed 11/126 (9%). Where applicable, the epidemiological classification for the quantitative studies was cross-sectional studies (55), prevalence studies (9), cohort studies (6), case–control (2), descriptive (1), longitudinal cohort (1), observational prospective (1), cross-sectional retrospective (1), cross-sectional observational (1), prospective observational (1), retrospective epidemiology (1), retrospective longitudinal (1), intervention studies (2), randomised study (1), non-randomised study (1), there was no additional information for eight studies.

**Figure 2:**
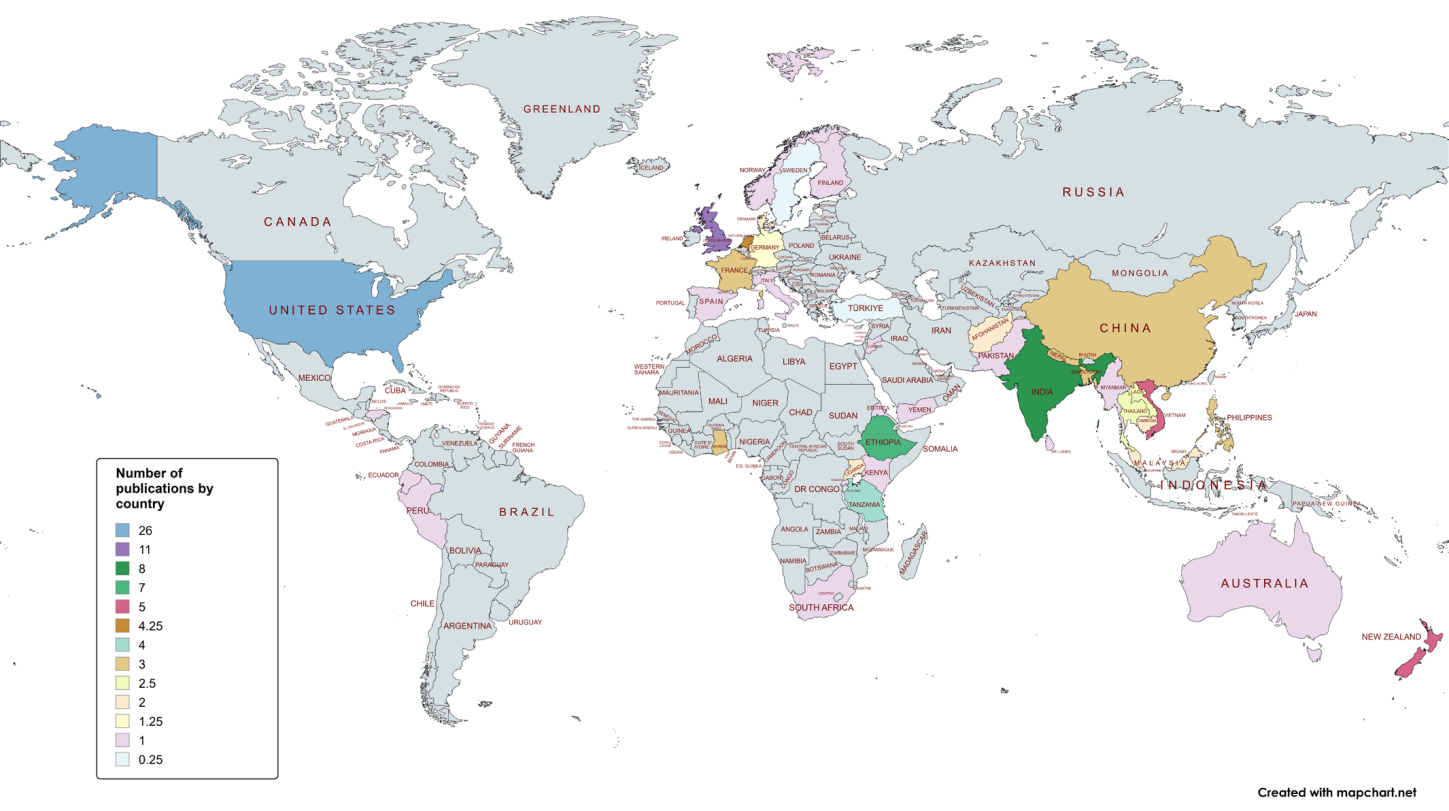
The number of studies retrieved by individual country.

There was evidence, as outlined in figure 2, from HICs 60/126 (47%), LMICs 41/126 (33 %), LICs 14/126 (11%) and upper-middle income (UMIC) 11/126 (9%) countries.

Of the included studies, 52 (36%) were published between 2000 and 2017, while the remaining (64%) were published from 2018. This increase in publications mirrors the increase in research to address health inequities and inequalities over the last decade, particularly following the COVID-19 pandemic.^48 49^ A summary of all the included studies, by the key indicators of interest, is provided in the online supplemental materials. The following text below provides a breakdown of the data by the specific thematic domains originating from the research questions in this review and by population group. In some studies, the evidence transects multiple questions. In such cases, the study has been allocated to the dominant question.

### Social determinants of health

Online supplemental table 1 summarises the existing evidence on SDoH and AMR. The impact of low socioeconomic status was evident in all studies. A cluster of five studies conducted in HICs the UK, Norway and New Zealand showed a direct association between deprivation as defined by the Indices of Deprivation for the respective countries and increased antibiotic prescribing.^62^,^66^ In addition to deprivation, Whyler *et al*^66^ identified further associations with higher rates of antibiotic dispensing among people of Pacific and Māori ethnicities located in New Zealand.

Evidence from HICs examining deprived housing conditions and overcrowding was associated with community-associated MRSA (CA-MRSA).^67 68^ Poor housing combined with Black ethnicity was also associated with CA-MRSA, although this association could not be statistically proven in the USA.^67 69^ In England, Black populations living in poor housing were associated with both CA-MRSA and hospital-acquired MRSA.^68^ In the USA, other indicators included use of Medicaid health insurance and requiring an interpreter, which were associated with Multi Drug Resistant (MDR) UTIs.^70^

In LICs and LMICs, six studies^71^,^76^ highlighted several SDoH and their respective impact on health-seeking behaviour. These determinants included occupation, education level, living environment, home location, rural areas and the distance from home to healthcare facilities. Negative impacts included inability to access appropriate healthcare, which led to inappropriate antibiotic use both generally and in the treatment of respiratory infections. Contributing factors to this misuse included distance to health facilities, transportation costs, females prioritising carer responsibilities and low education levels.^71 75 76^ Two papers used a One Health approach showing the juxtaposition of people and animals living in proximity to risks of AMR transmission.

### KAP studies

Online supplemental table 2 summarises the existing evidence on KAP studies. Knowledge was included as a determinant of health in the search terms because of its links with education, and an individual’s educational attainment is used as a measure of the SDoH. Due to the complexity of this review, these studies are analysed in a separate publication.

### Health seeking behaviour linked with self-medication

Online supplemental table 3 summarises the existing evidence on health seeking behaviour and self-medication with antibiotics. 12 studies reported health-seeking behaviours associated with self-medication, with 10 of these conducted in LICs and LMICs. 11 studies examined the general drivers of self-medication, and 1 study reviewed self-medication providing limited evidence about links to respiratory infections.^77^ At a macro level, self-medication was reported in over 87% of the population group in Yemen.^78^ Self-medication was considered as an acceptable ‘routine’ practice, driven by ease of obtaining antibiotics and poor governance in pharmacy stores.^79^,^81^

At a meso level, evidence of SDoH included low income levels, with self-medication being seen as a way of saving money, not being able to afford to see a medical practitioner and accessing medication quickly without losing income.^7780 82^,^84^ Evidence of low education levels, including being illiterate, was also associated with self-medication.^77 85 86^ A higher prevalence of self-medication was seen in rural locations.^79 83^ Ease of access, saving time and distance to healthcare facilities were also seen as contributory factors.^81 83 84 87^ At a micro level, increased antibiotic use was associated with some religions,^81 86^ perceived acceptable sharing of antibiotics at a family level^79 83 87 88^ and previous experience of successful treatment through self-medication.^83 84^ Female gender was reported as a factor contributing to self-medication in three studies,^85^,^87^ although male gender was positively associated with self-medication in two studies.^83 86^

### Health seeking behaviour: general factors

Online supplemental table 4 summarises the existing evidence on general aspects of health seeking behaviour. Pathways to access and use of antibiotics were discussed in four studies and included access via conventional and unconventional routes, unlicensed suppliers, misuse and understanding about antibiotics.^89^,^92^ Knowledge about antibiotics and their respective use was generally poor, complicated by poor descriptions of the actual drug being purchased. Female gender was associated with a higher incidence of antibiotic use, obtaining antibiotics from non-conventional healthcare routes and deferring treatment decisions to male relatives.^91^,^93^ Higher levels of antibiotic prescribing were evident for children of mothers with Pakistani ethnicity in England.^94^ Antibiotic prescription guidelines were not consistent for refugees in comparison to other population groups in Germany.^95^

### Studies reporting resistant pathogens and infections

Online supplemental table 5 summarises the existing evidence on prevalence studies primarily examining a range of infections. The evidence is presented by organism, resistance mechanism and clinical syndrome including antibiotic use:

*Staphylococcus aureus* (both methicillin susceptible and resistant) MRSA, CA-MRSA infections were reported in 12/38 studies conducted in HICs with inconsistent evidence. Ethnicity, lower socioeconomic status, poor housing, homelessness and living in areas with high HIV prevalence were associated with higher rates of *S. aureus* carriage and/or MRSA.^96^,^101^ In contrast, other studies did not find associations between *S. aureus* with ethnicity, homelessness and education level.^102 103^

Extended-spectrum beta-lactamases (ESBL) producing Gram negative bacteria were reported in 7/38 studies, with six studies conducted in LICs and LMICs and one from an HIC. Determinants included poor living conditions, poor WASH facilities, low educational level, animal husbandry including density of pigs and poultry and distant healthcare facilities. Risk factors included prior antibiotic use, age, prior UTI, prior hospitalisation and use of antibiotics.^104^,^109^ However, there was conflicting evidence about binary gender^105 106^ education level and prior use of antibiotics in children.^110^

Risk factors for respiratory infections included age, ethnicity and urbanicity in relation to penicillin-resistant *Streptococcus pneumoniae*. There were differences in incidence and the proportion of cases between Black and White ethnic groups.^111^ Rural areas showed higher carriage rates for *S. pneumoniae* and antibiotic use for respiratory infections.^112 113^ In Ethiopia, female sex and malnutrition were associated with increased bacteraemia and community-acquired pneumonia.^114^

UTIs were studied in 2/38 from UMICs. Tribal people with poor antibiotic knowledge and behaviours were linked with an increased risk of antibiotic resistance,^115^ and microbial resistance to antibiotics varied by age in patients in Bangladesh.^116^

The incidence of both *S. aureus* bacteraemia and *Escherichia coli* bloodstream infections was higher in Pacific peoples, Māori and indigenous Australians.^117 118^ However, in patients with a history of spinal cord injury and subsequent -MDR bacteraemia, no associations were identified with sociodemographic indicators.^119^

Evidence of multi drug resistance was identified in migrant populations, with asylum seekers and migrants being found to have high levels of MDR ranging from 7% to 45% at screening centres and while hospitalised.^120^,^124^ Pathogens included MRSA, ESBL-producing organisms, *Pseudomonas* spp*, E. coli, Aeromonas* spp and various species of *Acinetobacter*. One study found older age, region of origin and length of migration associated with AMR.^123^

Prevalence of antibiotic prescribing and use of non-prescription antibiotics was reported in 3/38 studies from HICs, the evidence mirrored the results under SDoH as shown in, online supplementary file table 1 . Antibiotic prescriptions were significantly higher in deprived populations and Hispanic populations, particularly in patients with poor health and who spent longer periods of time at home.^125 126^ Use and intention to use non-prescription antibiotics was also observed across all race/ethnic groups.^127^

Evidence of skin and soft tissue bacterial infections included the homeless^96^ and migrant populations.^120 128^ There were no studies of surgical site infections by the defined populations.

### Interventions

#### General interventions

Online supplemental table 6 summarises the existing evidence on two general interventions which examined (a) whether public facing AMR initiatives in the UK were inclusive and (b) the feasibility of using urine bioassay to assess antibiotic consumption in the Philippines.

### Interventions including public engagement and co-production with different stakeholders

Online supplemental table 7 summarises the existing evidence. The evidence included 11 studies from LMICs 6/11, UMIC 1/11 and 4/11 HICs. No studies were conducted in the UK. Studies were primarily aimed at increasing people’s awareness about AMR and reducing antibiotic use. Interventions ranged from co-production, working with communities to ensure studies were context specific to public awareness campaigns and quality improvement strategies for health system delivery. Study methods were supplemented with medical humanities, use of photovoice, photography, films and theatre productions.^129^,^132^ Evidence was limited due to the small number of studies. Although awareness events were seen as useful, communities did not perceive AMR as being a serious health condition and the campaigns did not lead to sustained behavioural changes. Concern was also raised about events being difficult to scale and potentially having inequitable uptake.^133^ Recommendations included a focus on structural determinants, for example, access to healthcare, improvement in WASH and addressing malnutrition.^129133^,^135^ This contrasted with evidence from the USA where focused literacy training interventions were reported as successful.^136 137^ In LMICs, one community engagement study conducted in Nepal outlined evidence of gender affecting access to healthcare. There was also evidence of using traditional medicines.^130 131 135^

### Evidence by subpopulation group

One of the key challenges was defining vulnerable and marginalised populations across different contexts and countries. Table 1 shows the evidence by population group, individual social determinants, indices of deprivation and the categories of protected characteristics. Population groups included low socioeconomic groups, ethnic groups, migrants including refugees and asylum seekers, the homeless, traveller communities and the disabled. There was one study for disabled people and none for the traveller communities.

**Table 1:** Heatmap showing the distribution of studies by population group, country income classification and the data collected by each respective study

Population group/indicators (n=studies)	Evidence by *The World Bank category	Age	Sex gender	Disability	Gender reassignment	Marriage civil partnership	Marital status	Pregnancy and maternity	Race	Religion/belief	Sexual orientation	Income level/poverty status	Employment/occupation	Work environment/conditions	Education attainment	Health deprivation and disability	Proximity of healthcare services	Affordability of healthcare	Environment for example, crime	Housing/living conditions	WASH	Size of family	Culture/beliefs	Multi morbidities
Low socioeconomic groups (n=50)	LMIC (25.5) HIC (12), LIC (9), UMIC (3.5)†	44	43				13		2	5	2	26	22	4	31	3	19	13	16	19	10	20	8	8
Ethnic groups (n=16)	HIC (15), LMIC (1)	14	12	1	1	1	1	1	3	1	2	7	1		6		1	3		4	2	1		1
Migrants, refugees and asylum seekers (n=15)	HIC (13.5), LMIC (1), UMIC (0.5)	13	12				1		1			4	3	1	5			3	1	1	1		1	6
Homeless (n=4)	HIC (3), LMIC (1)	4	3									2			1		2			3	1		1	1
Disabled (n=1)	HIC (1)	1	1	1																				
Gypsy Roma (0)																								

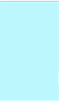
Protected characteristics (age; disability, gender reassignment, marriage and civil partnership, pregnancy and maternity; race; religion or belief, sex, sexual orientation).

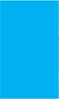
Additional-related indicator for protected characteristics.

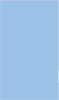
Indices of multiple deprivation (income, employment, education, health, crime, barriers to housing and services, living environment).

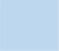
Additional-related indicator for indices of multiple deprivation.

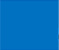
Additional indicator culture and beliefs.

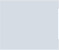
Multimorbidities.

Red=no studies, pink=1–4, yellow=5–10, pale green=11–15, green 16+.

* World Bank Group country classifications by income level.

† Study conducted in two countries=0.5.

HIC, high-income country; LIC, low-income country; LMIC, lower-middle income country; UMIC, Upper-middle income country; WASH, water, sanitation and hygiene.

## Discussion

The aim of this review was to describe available evidence related to SDoH and AMR, and the influence of this on health-seeking behaviours. Research to date in AMR has primarily adopted a biomedical approach with limited reference to social sciences.^1 138^ There is increasing evidence that SDoH significantly impact health outcomes at all life stages, and it is critical that their impact is taken into consideration within policy and interventions aiming to mitigate the emergence and spread of AMR.^12^ To facilitate this, a range of interventions that consider SDoH indicators is needed, aided by a data science approach.^25^ Failure to address the SDoH drives health inequalities,^139^ which subsequently increases the burden and associated impact of AMR.

This review highlighted the limited evidence on the impact of SDoH on AMR. Co-production and participatory research approaches remain underused; however, they are imperative for defining the context and supporting more sustainable change in outcomes and behaviours. A major gap remains in multidisciplinary approaches, particularly the inclusion of anthropology and ethnographic methods which would have enhanced and assisted in addressing the complex challenges in this field. Precarity has been defined as a relatively new indicator which provides a useful adjunct when considering the risks for people when they are exposed to multiple adverse situations.^140^

Research priorities in relation to SDoH vary considerably depending on the socioeconomic ranking of countries. Classifying countries by HIC/LMIC is itself problematic and driven by the World Bank classification for countries.^57^ This classification is based on Gross National Income and fails to adequately describe the variances between countries in the respective classifications and/or outline the different contextual factors, particularly in relation to LMICs.^141^ It leads to LMICs being considered as a monolith, failing to recognise how pockets of inequality exist within countries and often may translate inappropriately to the health systems and disease burden of the countries. When considering the SDoH of populations, it may also be time to consider how we classify countries in relation to disease burden and healthcare access and equity.

In the UK, there was a range of evidence covering the four domains and across a range of population groups. In other HICs, there is more research on migrant populations with the focus on examining the potential carriage and transmission of pathogens among this population. Conversely, in the LMICs, self-medication of antibiotics and its association with AMR infections is the focus of studies, with a limited approach to address the drivers of the behaviour and access to health services. This has resulted in many KAP studies assessing people’s knowledge and self-medication behaviour without examining the multifaceted drivers, particularly cultural and structural determinants. The choice of subject area also reflects the different global and country-specific priorities. For example, examining gender, equality and diversity indicators are accepted criteria in some countries but not always recognised or legal in others. This reinforces the requirement for context-specific research. It is important to recognise that there is a complicated relationship between gender, gender identity and anatomy which requires further consideration. We recognise that in certain situations, this may be more sensitive, for example, adults with UTIs which may affect their health-seeking behaviour.

There was a lack of clarity about defining populations vulnerable to AMR. The evidence includes a range of populations, in varying numbers, with some population groups omitted and/or limited, for example, the disabled, homeless and traveller communities. Apart from KAP studies, there was limited fragmented evidence addressing the full range of SDoH and the corresponding intersections.

**Figure 3:**
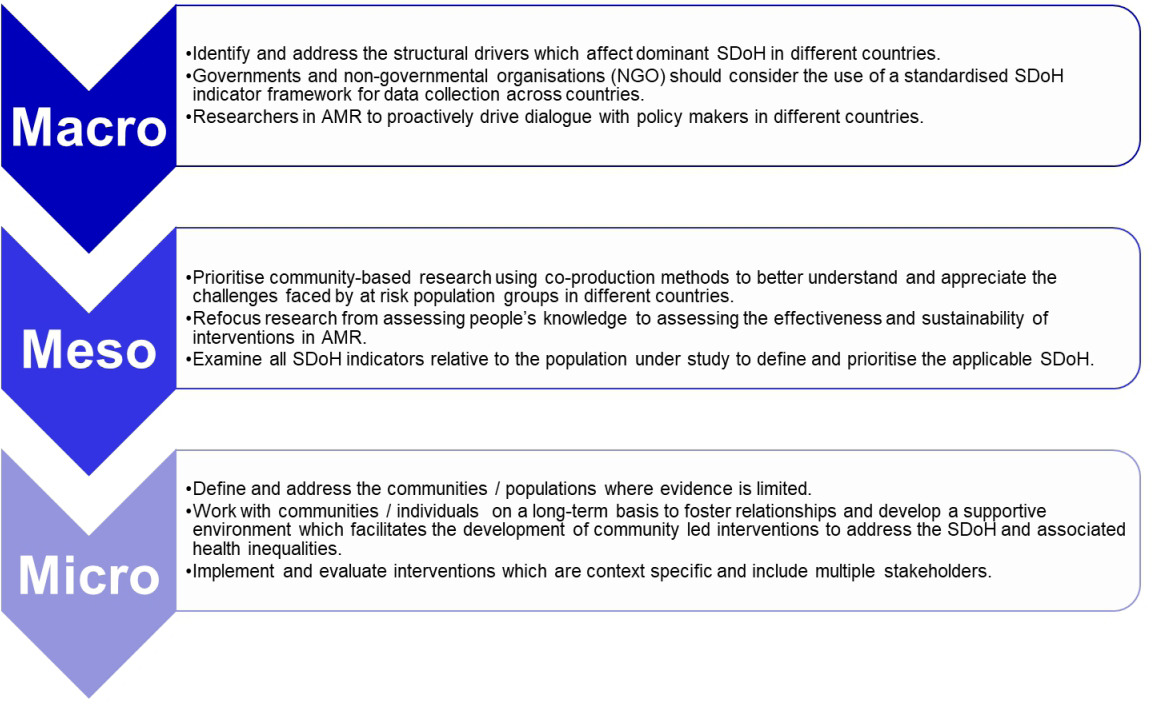
Summary of the key recommendations to consider the impact of the SDoH and AMR when developing policy and interventions. AMR, antimicrobial resistance; SDoH, social determinants of health.

This systematic review provides recommendations and guidance for a range of stakeholders in AMR including, global actors, for example, the WHO, policy-makers and non-governmental organisations (NGOs), across macro, meso and micro levels as outlined in figure 3. At a macro level, priority must be given to SDoH research to assist in the multidisciplinary approach which is required to address the challenges of AMR. Research funding has historically focused on biomedical research. This must now change; priority should be given to research focused on the SDoH. Without the inclusion of this research field, progress will be limited in addressing AMR. Policy recommendations include the importance of identifying and addressing the structural drivers which affect the SDoH. Failure to address these drivers will exacerbate health inequalities, disproportionately impact people already affected by deprivation and may be compounded, causing multidimensional poverty.

The WHO, governments and NGO should consider the use of a standardised framework for data collection and analysis of SDoH. This would assist in prioritising which determinants have the greatest impact on AMR in different populations. Research findings from standardised SDoH frameworks should be incorporated into country NAPs and evaluated annually. A major gap in the AMR literature is the lack of evidence on engaging at macro, systems level to address the environmental and societal drivers of AMR, which often those most affected have no control over. While better understanding and addressing the impact of how people’s culture and agency affects healthcare access is important, unless there are major shifts in policy, the SDoH in relation to AMR cannot be effectively addressed.

At a meso level and micro level, research has focused on assessing and changing people’s behaviour, particularly in relation to obtaining and consuming antibiotics. Scholars are advocating for a greater use of co-production methods, collaborating with individuals and communities to identify the determinants that pose the greatest impact on people. Sustained co-production will enable community engagement and better understanding of policy and structural changes required to accelerate optimised use of antibiotics. Marginalised populations are often the most vulnerable; therefore, consideration needs to be given to ensure research is not repetitive and is primarily focused on delivering long-term sustainable changes to improve people’s lives.

### Limitations

In this review, we adopted a broad approach to capture the availability of global evidence. As a result, a wide range of evidence is presented rather than a more narrow-focused approach. The lack of clarity about defining population groups posed challenges to the selection and exclusion of papers which may have introduced bias. Although certain vulnerable populations, for example, the elderly, may appear understudied in this review, this is incorrect. Studies had been undertaken but were not totally applicable for this review as they did not meet all review criteria. In some studies, the included infections for this review were also combined with other infections. These studies were included if the other domains were also met. The inclusion of terms applicable to one health may have returned more records; however, we focused on human health. People with STIs were excluded as sex workers had been excluded as this is a defined specialist field. We acknowledge that STIs can occur in non-sex workers; however, there may have been potential confusion in trying to segregate the cases if they had been included.

## Conclusions

The existing evidence demonstrates a lack of research examining the complete range of SDoH and their associated indicators. There is also a gap in understanding the upstream systems which create inequalities and negatively impact SDoH for different populations. There was considerable variation in the available data, and the inconsistent approach to data collection exacerbates the difficulties in determining which SDoH, and/or a combination of determinants, have the greatest impact on health inequalities in different countries. A range of evidence was also available: for demographic information, equality and diversity protected characteristics, indices of deprivation, and culture and belief. The wide range of definitions used to define vulnerable populations caused challenges undertaking the review. There was an evidence gap in research examining people marginalised due to disability, homelessness, rural–urban migration including within country, refugees displaced due to war and climate and traveller communities. Reported interventions focus on awareness programmes, with little evidence of the impact of sustainable behaviour changes.

Recommendations from this review include policy-makers and related stakeholders prioritising SDoH research, with associated funding, to assist in the multidisciplinary approach to address the challenges of AMR. Policy-makers and researchers are encouraged to focus on the drivers of health inequalities rather than behavioural changes, particularly in the field of self-medication with antibiotics. Strengthening the development of data science, with identification of the determinants which require the most focus, is required in this field. Further research is required in cooperation with population groups where evidence is limited.

## Supplementary material

10.1136/bmjgh-2024-017389 online supplemental file 1

## Data Availability

There was no data sharing plan set out at the beginning of this study. Specific requests for the data for valid academic reasons as judged by the first and last authors will be granted, with appropriate data sharing agreement, and should be sent to the chief investigator Dr Esmita Charani. For the purpose of open access, the author has applied a CC BY public copyright licence to any Author Accepted Manuscript version arising from this submission.
