# Supplementary material for: The intersection of the social determinants of health and antimicrobial resistance in human populations: a systematic review
Source: BMJ Glob Health. 2025 May 30;10(5):e017389. doi: 10.1136/bmjgh-2024-017389 (PMC12128480; doi:10.1136/bmjgh-2024-017389)
Supplement: online supplemental file 1 [file bmjgh-10-5-s001.pdf]

Supplementary file

Evidence on the intersection of the social determinants of health and antimicrobial resistance in human populations: A systematic review

Alison Shutt, Diane Ashiru-Oredope, PhD, James Price, PhD, Maria Clara Padoveze, Nusrat Shafiq, Emma Carter, Amrita Ghataure, Sameed Shariq, Alison Holmes, MD, Esmita Charani PhD. 2025

**Content**	**Page**
**Evidence for primary research question A -** What evidence is available on the SDoH as defined by gender, race, ethnicity, socioeconomic status on infection related health seeking and health provision behaviours?	2
**Table 1:** Included studies primarily focusing on the social determinants of health and AMR	2
**Table 2** Included studies focusing on knowledge, attitudes and practice in different populations in relation to AMR	8
**Table 3:** Included studies addressing health seeking behaviour categorised by self-medication with antibiotics	21
**Table 4:** Included studies addressing general aspects of health seeking behaviour in relation to AMR	27
**Evidence for primary research question B**- What evidence is there on the burden and impact of AMR in different populations disaggregated by the socio - economic and demographic variables, e.g., race, ethnicity, social deprivation, and gender?	36
**Table 5:** Included studies addressing the prevalence of AMR in vulnerable populations	36
**Evidence for secondary question A -** What evidence is there from the global literature on AMR related interventions which specifically target marginalized / vulnerable populations?	56
**Table 6:** Included studies examining general interventions in relation to AMR	56
**Evidence for secondary question B –** What evidence is there on existing AMR related interventions including public participation and co production targeting low socio-economic populations?	57
**Table 7** Included studies that included evidence of participatory action with populations considered at risk of AMR	57
**Search terms** The search terms used to extract the studies from the selected data bases and the search strategy.	61
**Table 8 Kappa score**	72
**References**	73

Evidence for primary research question A - What evidence is available on the SDoH as defined by gender, race, ethnicity, socioeconomic status on infection related health seeking and health provision behaviours?

Table 1: Included studies primarily focusing on the social determinants of health and AMR

Author, Year, Country, region and reference	Population sample (n)	Indicators of social determinants of health included as data	Evidence of health seeking behaviour	Infection related data	Study design and aim	Key findings
Afari-Asiedu S *et al*. 2020 [1]<br><br>Ghana<br>Kintampo North, South Districts Bono, East Region of Ghana	Men, women and children from 1,100 rural households.	Socioeconomic status, place of residence, age, sex, educational level, occupation, and marital status. Socioeconomic deprivation. Out of pocket expenses for healthcare including use of drug pedlars.	Behaviour associated with obtaining antibiotics	Inappropriate antibiotic use.	Quantitative and qualitative observational study examining determinants of inappropriate antibiotic use at the community level. Assessment of household factors which affect antibiotic use.	3,193 household members, 676 (21.2%) had used antibiotics for 761 episodes of illness and 659 (86.6%) were used inappropriately. 489 (64.3%) were used without prescription and 345 (45.3%) treatment courses were not completed. Instructions for use were not followed in 336 (44.15%) episodes. 232 (34.3%) were between the ages of 21 - 40 years, 433 (64.0%) were females, 250 (37.0%) had no education, 456 (67.5%) were unemployed and 364 (53.8%) were not married. No health insurance and not seeking healthcare from health facilities and pharmacies were associated with inappropriate use. Factors affecting which drug suppliers were used included distance to facility, cost of medicine, trust in supplier, popularity of facility, severity of disease and age of patient.
Babu G *et al.* 2018 [2]<br><br>India<br>Puducherry	Fishermen n=292 (including fishing related activities) in rural coastal villages.	Employment and work environment, illiteracy, socioeconomic status. Individual history including alcoholism, smoking, chewing pan.	Attitude to health seeking practices after occupational injury.	Wound infections caused by occupational injury. Culture and sensitivity of wound swabs.	Quantitative, cross-sectional study including structured questionnaire, conducted over six months. Determining a) the bacterial profile and antimicrobial resistance prevalence b) factors affecting health seeking behaviour.	269 (92.1%) of participants did not undertake first aid after injury and 254 (87.0%) did not visit the hospital after injury. 275 (94.2%) were not vaccinated after injury. From the 292 wounds, *Staphylococcus aureus* was isolated in 136 (53.1%), coagulase-negative *Staphylococcus* spp. (CONS) 34(13.3%), *Pseudomonas* spp., 23 (9.0%), *Streptococcus pyogenes*, 22(8.6%), *Escherichia coli*. 20 (7.8%). Antimicrobial susceptibility testing showed 83 (61.0%) of *S. aureus* and 11 (32.3%) CONS were methicillin resistant. 14 (60.9%) of *Pseudomonas spp*., 11 (55.0%) of *E. coli* and 2 (33.3%) of *Klebsiella pneumoniae* isolates were Extended Spectrum Beta Lactamase (ESBL).

Author, Year, Country, region and reference	Population sample (n)	Indicators of social determinants of health included as data	Evidence of health seeking behaviour	Infection related data	Study design and aim	Key findings
Boo, Y. Y.; *et al.* 2021[3]<br><br>India<br>Delhi, Meerut, Kolkata, Indore, Mumbai, Nagpur, Hyderabad, Chennai	Indian children n= 1424, 0 to 5 years old living in peri urban slums.	Sex, child's age in months, size of child at birth, whether breastfed, education level of mother, source of drinking water, type of toilet, characteristics of home e.g. floor and wall material, religion, caste, tribe, number of household members, type of cooking fuel, wealth index, availability of mosquito bed net, use of tobacco, health insurance, marital status of the mother, distance to health facilities.	Access to healthcare facilities.	Respiratory tract infection (RTIs), Gastrointestinal infection (GI)	1. Investigate the association between individual, household and slum level determinants and risks of RTIs and GI infections 2. Analysis of the determinants linked to phenotypic symptoms for named infections. Quantitative cross-sectional study using the National Family Health Survey data set 2015 to 2016 for the eight cities. Multilevel logistic regression analysis for the association between exposures and outcome variables.	Individual level: Fever and cough (FeCo) increased prevalence in younger children. Higher odds- of developing FeCo if Mother perceived child as being born smaller than average. Many variables at individual, slum, and household levels not statistically significant. Dry toilet had protective trends against FeCo. Animal dung fires - higher levels of FeCo. Mothers not using tobacco lower levels of FeCo. Slum level: FeCo was more prevalent where the community perceived the greatest distance between their home and the healthcare centre.
Casey, J.A. *et al*. 2021[4]<br><br>United States America (USA) California	n=450,612 men and women from Kaiser Permanente Southern California (KPSC) and 150,740 from Sutter Health in North Carolina. Ethnicities included Hispanic, Non-Hispanic, Asian, Pacific Islander, Black, White, and other.	Ethnicity, sex, age, marital status, occupation. Socioeconomic deprivation, work environment, affordability for health insurance.	Treatment seeking behaviour for a urinary tract infection (UTI).	UTI	Quantitative, case control study using electronic health record data to assess sociodemographic factors associated with UTIs and multi drug resistant (MDR) UTIs from 2015 - 2017. Examine whether individual or community-level sociodemographic factors were related to risk of UTI and risk of MDR UTI.<br>Identify and reduce sociodemographic disparities in the outcomes.	Three sociodemographic factors: use of Medicaid, using an interpreter, and community deprivation were weakly associated with increased risk of MDR UTI. Medicaid and requiring an interpreter were associated with an 8% and 36% increase in risk of MDR *Escherichia coli (E. coli)* UTI at KPSC and a 9% and 28% increase at Sutter Health, respectively. Pyelonephritis cases at KPSC, requiring an interpreter or living in a high-deprivation community were more likely to be MDR *E. coli*. At Sutter differences in MDR status by sociodemographic variables were not observed.
Caudell M.A.; *et al*. 2018 [5]<br><br>Tanzania<br>Chagga, Arusha, or Maasai villages	n=391 rural village households (n=118 Arusha agro - pastoralists, n=100 Chagga Highland farmers, and	Ethnicity, level of education, distance to health facilities, water source / treatment, sharing water with livestock, type of toilet,	Access to healthcare facilities	Biological and socioeconomic risk factors for the transmission of *Escherichia coli (E. coli).*	Identify the biological and socioeconomic risk factors associated with carriage of resistant *E. coli* in deprived diverse ethnic groups. Mixed	Antimicrobial use in both humans and livestock was not associated with resistance. The primary predictors of antimicrobial resistance were related to transmission and the cultural differences of the various groups. Factors included access to water and consuming unboiled milk. Findings

Author, Year, Country, region and reference	Population sample (n)	Indicators of social determinants of health included as data	Evidence of health seeking behaviour	Infection related data	Study design and aim	Key findings
in Northern Tanzania	n=173 Maasai pastoralists)	children under 5 vaccinated, characteristics of livestock, employment.			methods cross sectional study. A survey including 226 items was administered to measure antibiotic use, including self-reported indicators of lay use, health-care visits, the number of antibiotics or packages in the household, and ownership of syringes and needles. Human stool samples were taken and liquid milk and swabs of milk containers from the households were also plated. Statistical analysis included 56 variables.	emphasised the need to increase resources for the improvement of hygiene and sanitation.
Covvey, J.R. *et al.* 2014 [6]<br><br>United Kingdom Scotland	n=5.1 million men, women and children attending primary care clinics general practice (GP) service.	Socioeconomic deprivation mapped against Scottish Index of Multiple Deprivation including income, employment, health, education/training, access to services, crime, and housing. Data were stratified by patients age and sex and the antibiotic class.	Antibiotic prescriptions.	Proxy - antibiotic prescription.	Quantitative, cohort study evaluating association between deprivation and dispensed antibiotic prescriptions from 14 Scottish National Health Service boards from 2010 to 2012.	Consistent association between deprivation and increased rates of antibiotic prescribing; with the peak occurring in 40 to 59year-olds.<br>Patients from deprived areas showed less knowledge about the use of antibiotics. Drivers of antibiotic use included overcrowding, chronic illness linked with living in deprived areas, the impact of health behaviours including smoking and alcohol use were evident. Women were shown to be more affected by deprivation.
See, I. *et al.* 2017 [7]<br><br>United States America (USA) across 9 states	Centres for Disease Control and Prevention Emerging Infections Programme surveillance data. n=2609 adults with invasive community-associated methicillin resistant *staphylococcus aureus* (CA MRSA) between 2009 to 2011 eligible for inclusion.	Socioeconomic deprivation, education status, race, employment status, housing including crowding, rural areas, health insurance, poverty, income equality index, medically underserved area.	Lack of access to treatment briefly considered.	Community acquired methicillin-resistant *Staphylococcus aureus* (CA MRSA)	Quantitative retrospective cohort study investigating factors associated with differences in MRSA incidence and proportion of racial disparity mediated by socioeconomic factors.	Crude annual invasive CA MRSA incidence was 7.60 per 100 000 in Black adults and 4.59 per 100 000 in white adults (rate ratio 1.66; 95% CI, 1.52 to 1.80). In the mediation analysis after accounting for designated underserved areas, education, income, housing value and rural status, 91% of the racial disparity was explained. There was no significant link between Black ethnicity and CA MRSA; the differences were related to social factors.

Author, Year, Country, region and reference	Population sample (n)	Indicators of social determinants of health included as data	Evidence of health seeking behaviour	Infection related data	Study design and aim	Key findings
Svalestuen, S. *et al.* 2022 [8]<br><br>Norway<br>National study	Primary care prescription data for n= 734359 children under 3 years old between 2006 to 2016.	Parents education level, poverty measured using household income, sex, travel distance to healthcare facility.	Children`s treatment is dependent on how parents seek treatment which is affected by socioeconomic status and their level of education.	Proxy - antibiotic dispensing.	Quantitative retrospective longitudinal study at municipality level investigating the association between population education levels and growth trajectories in antibacterial dispensing rates.	Municipalities with low levels of population education have predicted reductions of approximately two prescriptions per 100 children, while municipalities with comparatively high levels of population education have predicted reductions approximately equal to ten prescriptions per 100 children over the period. Recommendation for antimicrobial stewardship programmes to consider social demographics e.g., education that may affect health behaviour.
Thomson, K. *et al.* 2020 [9]<br><br>United Kingdom<br>England	n=29,631 GP practices included for antibiotic prescribing data for adults and children.	Deprivation - Index of Multiple Deprivation (IMD) at Lower Layer Super Output Area (LSOA), ethnicity, measure of urbanity.	Assess whether differences in antibiotic prescribing are attributable to health need or health seeking behaviour or differences in access for GP services.	Prescribing of antibiotics	Quantitative, prevalence study using antibiotic prescribing data from the NHS Business Services examining the association between antibiotic prescribing and area-level deprivation. The proportion of broad-spectrum antibiotics prescribed by area-level deprivation and geographic regional influence on prescribing rates.	Although there was a reduction over time in prescribing there were significant inequalities in antibiotic prescribing linked to deprivation, the highest levels of deprivation had the highest prescription levels even when adjusted for health need. However, there were higher levels of broad-spectrum antibiotics prescribed in more affluent areas. There was also considerable variability across regions with the East and Northeast of England having the highest rates of prescribing.
Tosas Auguet, O. *et al.* 2016 [10]<br><br>United Kingdom, England. Lambeth, Southwark, and Lewisham boroughs in Southeast London	n=471 patients who attended secondary and tertiary hospitals and community health units.	Socioeconomic deprivation including indices of deprivation 2010, homelessness, housing –overcrowding, immigration to the UK, ethnicity.	People who had attended a healthcare facility.	Methicillin-resistant *Staphylococcus aureus* (MRSA)	Quantitative cross-sectional study mapping MRSA cases to area-level aggregated socioeconomic and demographic data.	Community acquired (CA) and hospital acquired (HA) MRSA were both positively associated with household deprivation, Black African ethnicities and residences in purpose-built flats and tenements. HA MRSA was associated with poor health and living in a communal care home. CA MRSA was linked with overcrowding, low income, homelessness, and recent immigration to the UK.
Walls G. *et al.* 2015 [11]<br>New Zealand<br>Manukau	n=94,242 people in Counties Manukau District Health Board. Diverse deprived populations in both urban and rural areas.	Ethnicity, income, education, employment, housing - people to a bedroom ratio. Socioeconomic status including access to a vehicle, access to a telephone.	Health provision at primary care level.	Proxy - antibiotic prescribing.	Quantitative, prevalence study including the Area Unit overall New Zealand Deprivation Index to determine the socioeconomic factors that may influence antimicrobial prescribing.	The number of antimicrobial scripts prescribed for an Area Unit varied from 0.52 to 2.71 scripts per person per year (median 1.11, interquartile range (IQR)= 0.36)<br>The Deprivation Index was positively correlated with per capita antimicrobial prescriptions. The most deprived decile population received roughly twice the number of antimicrobial scripts per

Author, Year, Country, region and reference	Population sample (n)	Indicators of social determinants of health included as data	Evidence of health seeking behaviour	Infection related data	Study design and aim	Key findings
						capita as the least deprived decile (0.84 versus 1.76, estimated ratio 2.1, 95% confidence interval (CI) [1.1, 3.1]). Percentage of the population unemployed and percentage with no educational qualification were positively correlated with the prescriptions. Identifying as being of Pacific Island or Māori ethnicity, smoking tobacco and no access to transportation or communication was also positively correlated with increased antibiotic prescriptions. Multiple regression strongly identified antimicrobial prescriptions associated with overcrowding and some links with Māori ethnicity.
Whyler N. *et al.* 2018 [12]<br><br>New Zealand National	n=4315871 adults and children registered with general practitioners in 2015. National Pharmaceutical Collection - 5204143 antibacterial courses dispensed.	Socioeconomic deprivation and the effect of age and ethnicity on the rate of dispensing.	Health provision - antibiotic dispensing.	Proxy antibiotic dispensing.	Quantitative study from 1 January 2015 to 31 December 2015 mapping rates of dispensing per 1,000 population per day to deprivation, age, and ethnicity.	Antibacterial dispensing rates were higher for people with Māori or Pacific ethnicities in comparison to people from other ethnic groups. Antibacterial dispensing rates were also higher for people from socioeconomically deprived areas.
Yong S. *et al.* 2022 [13]<br><br>Cambodia Phnom Penh, Kampong Chhnang, Prey Veng Province	n=512 households in 4 urban communities and 8 rural communities.	Gender, age, marital status, level of education, occupation, level of income of care givers. Age and sex of children, family size, monthly income and financial status of children's family and family relationships.	Source and inappropriate use of antibiotics by the care givers for children under 5 years.	Proxy antibiotic use	Quantitative cross-sectional study and questionnaire interviews were conducted to investigate the determinants of inappropriate antibiotic use.	Most caregivers in both settings attended healthcare facilities to obtain antibiotics. Nearly 80% of children had inappropriate antibiotic use. There was a significant difference between settings - rural 53.3% and urban 26%. Antibiotics were incorrectly used for fever (74.1% in urban and 73.1% in rural), and common cold/running nose (52.5% in urban and 52.6% in rural). Antibiotic use for bacterial infections was 82.1% urban, 52.6% rural. Although most caregivers gave the correct form, dose and time, antibiotics were given less frequently in urban settings. Concerns were not administering the complete course and inappropriately using left over medication.
Zheng, C. *et al.* 2021[14]<br><br>Nepal<br><br>National study	n=15372 children under 5 years living in urban and rural settings across 3 consecutive demographic health surveys.	Age, sex, socioeconomic status - wealth index, education status. Housing including household size, household members, home environment e.g.,	Health seeking patterns and sources of antibiotics.	Respiratory tract infection, fever and diarrhoea.	Examine antimicrobial resistance factors associated with antibiotic use in acute respiratory infection (ARI).	Prevalence of ARI, fever and diarrhoea was highest in children aged 6 to 23 months. The prevalence for the three conditions decreased over the ten-year period. However, the number of children under 5years receiving antibiotics increased. Antibiotic use in rural areas increased during each time interval with 2016 data

Author, Year, Country, region and reference	Population sample (n)	Indicators of social determinants of health included as data	Evidence of health seeking behaviour	Infection related data	Study design and aim	Key findings
		water by well, sanitation-external latrines. Nutritional status. Maternal education access to healthcare and vaccinations.				demonstrating rural antibiotic consumption for ARI and fever exceeding urban regions. The prevalence of ARI was highest in the hill areas in all three surveys. Measles vaccination, basic vaccinations, nutritional status, sanitation, and access to health care were associated with antibiotic use. Decrease in people seeking care from the public sector, in contrast to the private sector which increased. Pharmacies remained a predominant healthcare provider while unregulated traditional healers and shopkeepers were the minority providers.

Table 2 Included studies focusing on knowledge, attitudes and practice in different populations in relation to AMR

Author, Year, Country/Region and reference	Population sample (n)	Population demographics and social determinants of health	Evidence of health seeking behaviour	Infection related data	Study design and aim	Key findings
Afari-Asiedu, S.; *et al.* 2020 [15]<br><br>Ghana Kintampo North and South Districts	n=55 Ghanaian men and women community members from rural forest and savannah areas.	Age, sex, occupation, knowledge, cultural practices including rituals.	Obtaining medication from hospitals, pharmacies, and unlicensed vendors.	Proxy use and obtaining antibiotics	Qualitative, exploratory, ethnomethodology study including focus groups and in-depth interviews to explore inappropriate antibiotic use and confusing antibiotics with other medicines.	Analysed separately.
Al Baz, M.; *et al.* 2018 [16]<br>Jordan<br>Irbid, Baqaa South, Taybeh and Marka	n=245 Palestinian refugees (men and women) in urban and rural locations.	Sex, age, education status, employment status, income level, location of residence.	Attending United Nations Relief and Works Agency (UNRWA) health centres.	Proxy antibiotic use.	Quantitative; cross sectional study.<br>To assess knowledge, attitude and behaviour regarding antibiotic use.	Analysed separately.
Alkirawan, R.; *et al.* 2022 [17]<br><br>Netherlands National study	n=12 newly arrived Syrian refugees. Men n=5 and women n= 7.	Sex, age 30 to 46. Level of education, former profession. Health beliefs.	Adapting to new health system - Dutch primary care.	Antibiotic use as a proxy.	Qualitative study using semi-structured interviews to explore the perspectives and expectations about antibiotic use and prescribing.	Analysed separately.
Barker, A. K.; *et al.* 2017 [18]<br>India<br>Haryana<br>Sikandarpur Badha, Bhirawati, Silani, Kadipur and Pratap Nagar	n=20 men and women from villages and roadside stalls in both rural and urban areas.	Sex, age, education status, illiteracy, monthly household income. Socioeconomic deprivation and associated health behaviour.	The impact of healthcare access, knowledge and income on antibiotic use and practices.	Proxy use of antibiotics.	Qualitative study using semi structured interviews and questionnaires to assess health literacy, antibiotic use and knowledge.	Analysed separately.

Author, Year, Country/Region and reference	Population sample (n)	Population demographics and social determinants of health	Evidence of health seeking behaviour	Infection related data	Study design and aim	Key findings
Bernadas, J. M. A. C. 2019 [19]<br><br>Philippines Manila	n=28 Filipino women (mothers or guardians and female senior citizens) in urban areas.	Socioeconomic deprivation.	Sourcing antibiotics.	Proxy obtaining and meanings of antibiotics	Qualitative study using focus groups to explore the meanings, experiences, and information sources for antibiotics.	Analysed separately.
Bogale, A.A.; *et al*. 2019 [20]<br><br>Ethiopia<br>Addis Ababa	n= 595 Ethiopian men and women from community urban homes.	Sex, age, ethnicity, location of home, educational status, occupation, household income.	Use and knowledge of antibiotics.	Proxy antibiotics	Mixed methods, cross sectional study to assess the knowledge, attitude, and practice of antibiotic self-medication.	Analysed separately.
Burtscher, D.; *et al*. 2021 [21]<br><br>Afghanistan, Kabul	n= 21 men and women over 18 years and caregivers of children, based in urban areas attending outpatients in the Ahmad Shah Baba (ASB) District Hospital. Pashto ethnicity and illiterate. Health care staff from the same institution n=18, n=351 patients and caretakers completed a questionnaire.	Age, sex, ethnicity, education status, number of children, household size, housing location.	Attending secondary care	Proxy - perceptions and use of antibiotics.	Mixed methods study conducted in two stages to explore the perceptions and attitudes toward antibiotics among patients, prescribers, and pharmacists.	Analysed separately.

Author, Year, Country/Region and reference	Population sample (n)	Population demographics and social determinants of health	Evidence of health seeking behaviour	Infection related data	Study design and aim	Key findings
Cheng, J.; *et al.* 2018 [22]<br><br>China<br>Anhui	Men and women n= 2760 living in rural village households.	Sex, age, socioeconomic deprivation, education status, health insurance, household size.	Behaviours associated with antibiotic use.	Proxy antibiotic knowledge and associated behaviours.	Quantitative cross-sectional study, to explore a) knowledge and behaviours of participants b) participants knowledge of antibiotics distributed by sociodemographic characteristics c) the relationship between level of knowledge about antibiotics and antibiotic use behaviours.	Analysed separately.
Corbett, K. K; *et al.* 2005 [23]<br><br>United States of America (USA)Colorado	n=992 (total). Non-Hispanic Whites and Hispanic Latino men and women living in urban households. n= 692 non-Hispanic whites and n=300 Hispanics.	Age, sex, education level, income level, internet access, children under 5 years and office visit.	Proxy- antibiotic knowledge and antibiotic use. Access to healthcare including preferred language to speak to health provider and for written material.	Respiratory tract infection, upper respiratory tract infection.	Quantitative cross-sectional study including a telephone survey in English or Spanish. Multivariable logistic regression conducted. The study examined relationships with ethnicity, primary language use, and antibiotic-related knowledge, attitudes, and awareness.	Analysed separately.
Crigger, N. J.; *et al.* 2004 [24]<br><br>Honduras Tegucigalpa, La Libertad.	n= 939 Honduran men and women in 2 rural areas including one poor and one farming community and 2 urban areas.	Sex, age, rural and urban location, education status, literacy levels. Questions to assess poverty included owning a car, domestic help and having a living room which wasn't used as a bedroom.	Proxy associated behaviours for obtaining and reasons for taking antibiotics.	Proxy use of antibiotics and reasons for consumption	A quantitative study comparing rural and urban areas using a model to assess choices and acquisition of antibiotics. Preparation to develop an educational programme for the designated population.	Analysed separately.

Author, Year, Country/Region and reference	Population sample (n)	Population demographics and social determinants of health	Evidence of health seeking behaviour	Infection related data	Study design and aim	Key findings
Dunn-Navarra, A.-M.; *et al.* 2012 [25]<br><br>United States of America (USA) Northern Manhattan	Urban Latino immigrant parents n= 154.	Age, sex, ethnicity, race, country of birth, time in the US, health care coverage, socioeconomic deprivation, income levels, education status. Children attending head start programme, weekly hours spent away from home by parent, health literacy abilities Short Test of Functional Health Literacy in Adults (S-TOFHLA) and Newest Vital Sign (NVS) English proficiency. Self-reported heath assessment.	Access to healthcare, preferred language for healthcare	Upper respiratory tract infection (URTI).	Quantitative study including a Knowledge and Attitude survey. The aims were to: a) describe the influence of health literacy on parental knowledge and attitudes/beliefs for URTI b) examine the correlation between two health literacy tools.	Analysed separately.
Emgard, M.; *et al.* 2022 [26]<br><br>Tanzania Moshi Municipal District, Kilimanjaro.	n=54 Mothers of children under 5 years old. Dispensaries and health facilities in urban and rural areas.	Socioeconomic deprivation, education level, marital status.	Access to healthcare for children.	Proxy use of antibiotics.	Qualitative study was undertaken with a phenomenographic approach. The aim was to understand the mothers' perceptions of antibiotic use in their children.	Analysed separately.

Author, Year, Country/Region and reference	Population sample (n)	Population demographics and social determinants of health	Evidence of health seeking behaviour	Infection related data	Study design and aim	Key findings
Francois Watkins, L. K.; *et al.* 2015 [27]<br><br>United States of America (USA) National study	Survey data for Hispanic men and women Year 2012 n= 4044<br>Year 2013<br>n= 3502<br>The participants represented a) adult consumers (all ethnicities); b) adult Hispanic participants c) primary, secondary and tertiary health care providers.	Sex, age, race, ethnicity, household income, household size, education, region.	Proxy - antibiotic knowledge and attitudes.	Upper Respiratory tract infection.	Quantitative study using online national survey data. The aim was to understand health care provider and consumer knowledge and attitudes that influence antibiotic use.	Analysed separately.
Gebeyehu, E.; *et al.* 2015 [28]<br><br>Ethiopia Northwest Ethiopia, Bahir Dar	Ethiopian households n= 1082 divided into urban areas n= 362 and rural n= 719.	Sex, age, family size, family monthly income, education status, including literacy, employment status, marital status, socioeconomic deprivation.	Inappropriate use of antibiotics in the community.	Proxy - antibiotic use.	Quantitative comparative cross-sectional study to determine inappropriate use of antibiotics and its associated factors.	Analysed separately.
Geta, K.; *et al.* 2022 [29]<br><br>Ethiopia Amhara Northwestern Regional State	n=232 men and women in urban secondary care public hospitals.	Age, sex, religion, marital status, level of education status, employment status.	Knowledge, attitudes and practice for antibiotics and antibiotic resistance.	Proxy antibiotic knowledge and use.	Quantitative cross-sectional survey using a questionnaire. The aim was to assess knowledge, attitudes and practices regarding antibiotic use and resistance.	Analysed separately.

Author, Year, Country/Region and reference	Population sample (n)	Population demographics and social determinants of health	Evidence of health seeking behaviour	Infection related data	Study design and aim	Key findings
Gunasekera Y.D.; *et al.* 2022 [30] Sri Lanka Western province, Uva	Communities - urban and indigenous rural people. Urban: female n= 132, male n= 48 Rural: female n=61male n= 83	Sex, age, education level, urban/ rural.	Antibiotic understanding and use.	Proxy: antibiotic use.	Quantitative study including questionnaires and interviews. The aim was to explore the respective community's knowledge, attitudes and practices of antibiotics and antimicrobial resistance (AMR), including use of antibiotics.	Analysed separately.
Ha, Thuy Van; *et al.* 2019 [31] Vietnam Kon Tum, Gia Lai, Dak Lak, Dak Nong, and Lam Dong.	Highland rural households n=1000. Kinh dominant ethnicity and other ethnic minorities.	Sex, age, marital status, ethnicity, socioeconomic status, education level, occupation.	Awareness about resistance, antibiotics and their use.	Proxy: Antibiotic use	Quantitative household study including a structured questionnaire and face-to-face interviews. The purpose was to explore people's awareness about antibiotic resistance, antibiotic use and identify associated factors.	Analysed separately.
Haenssgen, M. J.; *et al.* 2019 [32] Laos, Thailand Chiang Rai Salavan	n=2141 men and women living in rural villages.	Sex, age, ethnicity, nationality, education level, employment status, religion, cultural practices e.g., use of traditional healer.	Access to health services.	Proxy: use of antibiotics.	Aim was to a) describe antibiotic related knowledge, attitudes and practices b) Assess the role of antibiotic related knowledge and attitudes on antibiotic access from different providers. Quantitative study using a face-to-face questionnaire.	Analysed separately.

Author, Year, Country/Region and reference	Population sample (n)	Population demographics and social determinants of health	Evidence of health seeking behaviour	Infection related data	Study design and aim	Key findings
Halfvarsson, J.; *et al.* 2000 [33]<br><br>Vietnam<br>The Uong Bi District, Quang Ninh Province	n= 249 mothers of children under 5 years old and drug vendors from nine different ethnic groups located in rural areas.	Socioeconomic conditions, ethnicity, local cultural practices, including traditional Vietnamese medicine.	Perceptions and use of antibiotics for treatment.	Respiratory tract infection.	A combination of qualitative and quantitative methods. The aim was to assess mothers' perceptions and use of antibiotics specifically for the stated infection in children.	Analysed separately.
Hernandez-Diaz, I.; *et al.* 2019 [34]<br><br>Puerto Rico<br>San Juan East and West Puerto Rico	n =101 Latino parents of children under 6 years old in an urban secondary care hospital including the Emergency department.	Ethnicity, level of Medical Insurance, level of education, age, number of children.	Antibiotic use and preparation for educational tool.	Upper respiratory tract infection	Cross sectional study. The aim was to evaluate parents or legal guardians' knowledge, beliefs, behaviours, and adherence for antibiotic use.	Analysed separately.
Hika K.; *et al.* 2022 [35]<br><br>New Zealand<br>Apakura Marae, Papkura	n=30 Māori men and women aged 20 to 77	Systemic factors - effect of colonisation. Social factors - knowledge, health literacy and poverty barriers. Individual factors- illness perceptions and treatment beliefs.	Systemic factors - General Practitioner times and ratios. Social factors, access to healthcare, relationship with health professionals. Individual factors- natural vs western medicine.	Upper respiratory tract infection	A qualitative study. Overall aims were a) examine the experiences, perceptions and beliefs that Māori people have about antibiotics b) assess their use of antibiotics for the stated infection and c) knowledge about antimicrobial resistance.	Analysed separately.

Author, Year, Country/Region and reference	Population sample (n)	Population demographics and social determinants of health	Evidence of health seeking behaviour	Infection related data	Study design and aim	Key findings
Irawati, L.; *et al.* 2019 [36]<br><br>Malaysia<br>Jelutong District<br>Penang	n = 22 urban community residents. Including Malay, Chinese and Indian ethnic groups.	Gender, age, ethnicity, marital status, education level, employment status, monthly household income, socioeconomic deprivation.	Obtaining antibiotics.	Proxy - antibiotic knowledge and perceptions.	Qualitative study using semi structured interviews. The aim was to a) explore knowledge, attitudes and perceptions about antibiotics and resistance b) identify areas to be addressed when designing an educational intervention to increase residents' knowledge and change their attitudes and perceptions.	Analysed separately.
Khan, F.U.; *et al.* 2020 [37]<br><br>Pakistan<br>Swat, Khyber-Pakhtunkhwa	n=399 Pakistani men and women attending pharmacies in an urban, post conflict, region.	Socioeconomic deprivation, age, sex, level of education, occupation.	Obtaining antibiotics from pharmacies.	Proxy: antibiotics.	Qualitative; cross sectional study. The aim was to assess knowledge, attitude and practices for antibiotics and antibiotic resistance.	Analysed separately.
Kong, L.S.; *et al.* 2019 [38]<br>Malaysia<br>Kuala Lumpur	Urban tertiary hospital<br>n= 402<br>male (n=201) and female (n=201) patients 60 years and over.<br>Ethnicities:<br>Malay n= 233 (58%)<br>Chinese (111 27.6%)<br>Indian 55 (13.7%) and Other 3 (0.7%)	Age, ethnicity, educational level, occupation and employment status, monthly income related to healthcare.	Knowledge, expectations and sources of antibiotics.	Proxy – antibiotics.	Aim to assess participants knowledge on antibiotic use and their expectations towards the need for antibiotics and the relationship between an older age and the outlined factors. Quantitative; cross sectional study.	Analysed separately.

Author, Year, Country/Region and reference	Population sample (n)	Population demographics and social determinants of health	Evidence of health seeking behaviour	Infection related data	Study design and aim	Key findings
Larson, E. L.; *et al.* 2006 [39]<br><br>United States of America (USA) Manhattan	n = 30 Group 1 (n = 6) urban community members not in formal health system – no primary care provider / medical insurance. Group 2 (n =19) Participants with health insurance. Groups 1 and 2 included Latino women with Spanish as primary language, households with one plus preschool child/ children, residents of region. Group 3 (n = 5) two independent store staff, three healthcare staff.	Socioeconomic deprivation, education status, health literacy, costs for healthcare. (Health insurance Medicaid).	Ease of access to purchase antibiotics from a Bodega.	Self-prescription of antibiotics for multiple conditions including upper and lower respiratory tract infections and wounds.	Mixed methods, exploratory descriptive study. Outline knowledge, attitudes, beliefs and practices of community members use of antibiotics.	Analysed separately.
Lindenmeyer, A.; *et al.* 2016 [40]<br><br>United Kingdom (UK) National	n=23 migrants with Iranian, Polish, Pakistani, Indian, Iraqi, African, Chinese ethnicity who had been in the UK for >1 year, but <5 years.	Ethnicity, age, immigration status, reason for move and language.	Seeking antibiotics.	Proxy antibiotic use.	Qualitative interviews were undertaken with the aim to obtain maximum variation in the socio demographic factors.	Analysed separately.
Mason, T.; *et al.* 2018 [41]<br><br>United Kingdom Greater London	Stage 1 the public from affluent areas n= 384, stage 2 public from deprived areas n=384. Community pharmacists from both areas n=240. Age range 18 to 65. Ethnicity classified as White and non-White. Urban area.	Ethnicity, language, education, profession, profession of family member +/- health care professional and campaign exposure, socioeconomic deprivation.	Obtaining antibiotics and knowledge of antimicrobial resistance (AMR).	Proxy - obtaining antibiotics.	Quantitative; two stage cross sectional study. The aim was to assess the awareness and knowledge of antibiotic usage and antibiotic resistance between the two areas. The community pharmacists were recruited from the same areas. Questionnaires used.	Analysed separately.

Author, Year, Country/Region and reference	Population sample (n)	Population demographics and social determinants of health	Evidence of health seeking behaviour	Infection related data	Study design and aim	Key findings
McNulty, C. A. M.; *et al.* 2019 [42]<br><br>United Kingdom England	n= 2283 adults over 15 years old including n=777 parents with children under 5 years old. General public community venue across both rural and urban areas.	Age, sex, social grade, education level, children under 15 years old in household, attendance at GP / pharmacy in previous 12 months ethnic grouping, health literacy.	Antibiotic use.	Antibiotics used for: respiratory tract infection including upper and lower respiratory tract infections, urinary tract infection and cystitis.	Quantitative; cross sectional study. The aim was to understand public understanding and use of antibiotics.	Analysed separately.
McNulty C.; *et al.* 2022 [43]<br><br>United Kingdom England	n= 2022 adults aged 15+ including n=521 Black, Asian and minority (BAME) participants and n=406 aged 15 to 25year-olds.	Age, sex, social grade, level of education / reached qualification, ethnic grouping.	Antibiotic use.	Proxy – antibiotics.	Quantitative; cross sectional study to describe public attitudes and knowledge around antibiotic activity, use and resistance.	Analysed separately.
Norris, P.; *et al.* 2009 [44]<br><br>New Zealand Auckland and Wellington	Samoan men and women n= 13 interviews.<br>n=112 attending health care facilities in urban areas.	Ethnicity, age, sex culture.	Use of antibiotics.	Antibiotic usage for multiple conditions including upper and lower respiratory tract infections.	Qualitative in-depth interviews. The aim was to investigate understandings and use.	Analysed separately.
Paredes, J. L.; *et al.* 2022 [45]<br>South America, Peru	Peruvian parents n=231 based in rural areas including jungle and highlands. Primary health centres.	Sex, age, socioeconomic deprivation, education status, number of children, and respective age.	Obtaining antibiotics including without prescription.	Proxy: antibiotic use behaviour.	Cross sectional study across 6 centres. The aim was to describe the knowledge attitudes and practices.	Analysed separately.

Author, Year, Country/Region and reference	Population sample (n)	Population demographics and social determinants of health	Evidence of health seeking behaviour	Infection related data	Study design and aim	Key findings
Pattnaik, M.; *et al.* 2022 [46]<br><br>India<br><br>Odisha Tigiria	Households n= 1003 men and women aged from 18 to 60 and above from 25 rural villages.	Age, sex, education level, occupation, family type including joint, single, nuclear and extended. Ethnicity including general, schedule caste, schedule tribe, other backward castes.	Healthcare utilisation and antibiotic use.	Proxy - antibiotic use.	Quantitative; cross sectional study to assess knowledge, attitude and practices about antibiotic behaviour and antimicrobial resistance.	Analysed separately.
Russom, M.; *et al.* 2021 [47]<br><br>Eritrea<br>Gash Barka, Debub, Keih-Bahri, Anseba, Debubawi Keih-Bahri and Maekel	Men and women aged over 18 years living in 13 urban places<br>n= 2477.	Age, sex, religion, residence location (zone), education level, household size, occupation.	Use and knowledge of antibiotics.	Proxy: antibiotics.	Quantitative cross-sectional study to measure knowledge, attitude and practice of antibiotics and identify key determinants. Data was analysed in Statistical Package for the Social Sciences (SPSS).	Analysed separately.
Schuts, E.C.; *et al.* 2019 [48]<br><br>Netherlands<br>Amsterdam	Men and women from the Healthy Life in an Urban Setting (HELIUS) study<br>n= 21,617. Six ethnic groups including: Dutch, Surinamese, Ghanaian, Moroccan, and Turkish. urban areas.	Sex, age, ethnicity, migration generation level, education level, marital status, health status including 13 health conditions, smoking and alcohol history, Dutch language ability, perceived health.	Behaviours associated with antibiotic use.	Proxy: understanding of antibiotic use during influenza-like illness, pneumonia, fever, sore throat and bronchitis.	Aim to determine whether appropriate knowledge and use of antibiotics differ by ethnicity and whether knowledge on antibiotics is associated with antibiotic use. Quantitative; cross sectional study.	Analysed separately.

Author, Year, Country/Region and reference	Population sample (n)	Population demographics and social determinants of health	Evidence of health seeking behaviour	Infection related data	Study design and aim	Key findings
Sindato, C.; *et al.* 2020 [49]<br><br>Tanzania<br>Ilala, Kilosa and Kibaha districts	Men and women from urban and rural households n=828 participants across three regions.	Sex, age, marital status, education level, employment status, income source.	Knowledge and behaviours associated with antibiotic use.	Proxy: antibiotics.	Quantitative; cross sectional community-based study to determine the knowledge, attitudes, and practices regarding antibiotic use and antimicrobial resistance AMR among people with different livelihoods.	Analysed separately.
Ulaya G.; *et al.* 2022 [50]<br><br>Vietnam<br>Ha Nam Province, Northern region	n= 324 men and women from rural households.	Age, sex, education level, occupation, household wealth tertile, usual health facility, distance to health facility, medical insurance, frequency of media use, source of health information.	Health seeking information.	Proxy: antibiotic use.	Quantitative, cross-sectional study to assess the levels of awareness and knowledge of antibiotics and antibiotic resistance. Investigate the determinants of awareness and knowledge to inform the development of interventions.	Analysed separately.
Wang, Y.; *et al.* 2022 [51]<br><br>China<br>Eastern China - Zhejiang and Jiangsu province.	n=1494 (n=1379 eligible) men and women in two rural villages.	Sex, age, marital status, education years, occupation, annual household income, chronic disease history.	Obtaining antibiotics.	Proxy: antibiotics.	Quantitative, cross-sectional study aimed to understand antibiotic use and access patterns and their influencing factors.	Analysed separately.

Author, Year, Country/Region and reference	Population sample (n)	Population demographics and social determinants of health	Evidence of health seeking behaviour	Infection related data	Study design and aim	Key findings
Westerling, R.; et al. 2020 [52] Germany, Netherlands, Turkey, Sweden<br><br>National	n= 130 men and women in Turkey and Turkish migrants in the respective countries outlined, family physicians and pharmacists.	Citizens –sex, age, educational level. Family physicians - sex, age, professional experience. Pharmacists – sex, age, professional experience.	Antibiotic use and policy review of health systems across several countries.	Proxy: antibiotic use	Qualitative data was collected with the aim to a) explore the variation in implemented policies and the perceived access to antibiotics and information on antibiotic use among migrants living in the three European countries (EU) countries.	Analysed separately.
Whittaker A.; *et al.* 2019 [53]<br><br>Australia Melbourne	n =31 ethnic inpatients and interpreters in an urban public hospital and ethnic community members. Ethnicities included Chinese, Afghan, Chilean, Ethiopian, Samoan, South African, Indian, French, German, Pakistani, Pacific Islands, Sri Lankan, Thai, Dutch, New Zealand Vietnamese, Sudanese.	Age, sex, country of origin.	Hospital treatment, seeking treatment abroad, traditional remedies.	Upper respiratory tract infection	The aim was to outline the understandings and experiences of people with antibiotic use and antimicrobial resistance (AMR) to inform public antimicrobial stewardship and education programmes. Qualitative, semi-structured face to face in-depth interviews.	Analysed separately.
Xu, Y., *et al.* 2020 [54]<br><br>China Zhejiang and Shaanxi.	n=2924 parents from Zhejiang and n=3355 parents from Shaanxi whose children were 0 to 13 years old living in both rural and urban areas.	Sex of the child, age of the child, sex of the parent, parents' level of education, parents medical background, monthly household income, residential location.	Use of antibiotics.	Proxy: antibiotics.	Quantitative cross-sectional study to assess antibiotic use in children in one developed and one less developed region to identify regional parental behaviour disparities.	Analysed separately.

Table 3: Included studies addressing health seeking behaviour categorised by self-medication with antibiotics

Author, Year, Country, region and reference	Population sample (n)	Indicators of the social determinants of health included as data	Evidence of health seeking behaviour	Infection related data	Study design and aim	Key findings
Ahiabu, MA.; *et al.* 2018 [55]<br><br>Ghana<br><br>Koforidua and Awenare in Atiwa	Men, women, and children from 12 households. Observation from three urban and three rural pharmacy outlets. n=1548 transactions observed.	Ethnic groups in the communities include the Akan (major ethnic group), Ewe, Krobo and migrants from the three Northern regions. Household size, age, National Health Insurance scheme (NHIS) status.	Treatment practices in households for acute illnesses including antibiotic behaviour, self-medication, and antibiotic self-prescription.	Proxy: antibiotic use.	The study documents the treatment of acute illnesses in households and the antibiotic dispensing practices of medicine sale outlets in the Eastern region. Quantitative; qualitative and observational methods were used. Household interviews and medicine outlet observations were used.	Fever, abdominal, and respiratory symptoms were the most common causes of ill-health. Most (65%) medicine-use events involved self-treatment. Antibiotic without prescription in rural outlets (n = 139, 27.4%) was more statistically significant than urban pharmacies (n = 140, 13.5%). Lack of dispensing controls, community knowledge, inconsistent use of antibiotics, poverty and perceived barriers to formal healthcare influenced inappropriate use of antibiotics. Self-medication was the accepted method for both prevention and treatment. Treatment practices included sharing of medications, poor adherence to treatment, deliberate modification of prescribed drug dosages, caregivers adjusting dosing frequency with daily schedules and unconventional use.
Albawani S. M.; *et al.* 2017 [56]<br><br>Yemen<br><br>Sanaa City	n= 363 men and women in an urban area accessing community pharmacies.	Age, sex, marital status, education status, employment status, income level, medical insurance, medical centre.	Antibiotic self-medication.	Proxy: antibiotics.	To determine the prevalence of self-medication with antibiotics and associated risk factors. Quantitative, cross sectional study using questionnaires.	The prevalence of antibiotic use during self-medication was 87.1 % (58.2 % of males and 41.8 % of females). Only 49.5 % were aware of bacterial resistance due to antibiotic use. Most illness/symptoms treated during self-medication were common cold, cough, diarrhoea, and fever. The high cost of physician consultation was the main reason for self-medication with antibiotics. Information source was community drug dispensers. Although different factors were assessed, no association could be found between age, gender, material status, educational status, employment, monthly income or knowledge regarding bacterial resistance and self-medication with antibiotics. Self-medication with antibiotics may be related to low income with no access to medical insurance.

Author, Year, Country, region and reference	Population sample (n)	Indicators of the social determinants of health included as data	Evidence of health seeking behaviour	Infection related data	Study design and aim	Key findings
Anderson, A. 2021 [57]<br><br>Europe<br><br>Croatia, Slovakia, Slovenia, Cyprus	Data from the Eurobarometer survey series up to n=1000 per country.	Age, age left education, children under 10 and 10 to 14 in the household, location geography, GDP per capita, out of pocket health expenditure, control of corruption. Cultural dimensions including power distance, individualism, masculinity, uncertainty avoidance, indulgence, long term orientation.	Behaviour of self-medication.	Proxy: Antibiotics	a) Examined the determinants of self-medication with time-variant and time-invariant context analysis. b) examined the relationship between self-medication behaviour and antibiotic resistance at a national level. Quantitative cross sectional study using data from the Eurobarometer survey series. Included 2009, 2013, 2016, and 2018. The survey included a binary dependent variable for self-medication with participants having taken an antibiotic in the past 12 months. Analysis of antibiotics by a) prescribed by a medical practitioner and b) participants who used leftover antibiotics or obtained without a prescription.	Although antibiotic stewardship and antibiotic resistance are related biological mechanisms, they are also social phenomena. Therefore, structural challenges including inequality and poor access to healthcare should be addressed. Key findings included participants being more likely to self-medicate if located in countries with sustained inequality, out-of-pocket health expenditure, and corruption have an increased probability of self-medicating with antibiotics. National wealth (GDP per capita) negatively associated with participants self-medicating. Higher levels of income inequality positively associated with self-medication.
Annadurai K.; *et al.* 2017 [58]<br><br>India<br><br>Tamil Nadu Nellikuppam village, Kancheepuram district.	n=335 rural households	Age, sex marital status, education status including illiteracy, employment status, religion including, Hindus, Muslims, Christians, other.	Perceptions, practices, and diseases treated with self-medication.	Proxy: antibiotic self-medication	Evaluating self-medication practice and its determinants. Quantitative pretested semi structured survey.	Prevalence of self-medication was 53.43% and approximately 50% of the study participants stated it was harmful. Being female, a housewife and illiterate were indicators of higher levels of self-medication. Minor ailments (56.42%) and unavailability of doctors (22.34%) were the main reasons for self-medication. Previous experience of treating a similar ailment (18.43%) and requiring emergency care (11.17%) were other reasons. Advice about self-medication was sought primarily from Pharmacists (72.06%).

Author, Year, Country, region and reference	Population sample (n)	Indicators of the social determinants of health included as data	Evidence of health seeking behaviour	Infection related data	Study design and aim	Key findings
Ayana, H.; *et al.* 2021[59]<br><br>Ethiopia<br><br>Ambo Town	n =399 Ethiopian men and women purchasing antibiotics from rural and urban drug stores and pharmacies.	Age, sex, marital status, education status, employment status, location of residence (urban/rural).	Practices of obtaining medication without a prescription.	Proxy: antibiotic practices.	To assess the size of the non-prescription sale of antibiotics and associated factors. Quantitative cross-sectional study and qualitative observational study. A simple random sampling technique was used to select two pharmacies out of five and 8 drug stores out of 27 drug stores in the town.	The mean age was 36.32 years. 214 (53.6) were males, 228 (57.1%) were married, 191 (47.9%) were orthodox by religion, and 291 (72.9) were urban residents. One-fourth of the participants were unable to read and write. Non-prescription use of antibiotics was 43.2%. Reasons 107(62.2%) to save time, 111 (64.5%) to save money, 111 (64.5%) to get well quickly, 64% previous successful treatment of the same disease. 110 (64%) thought antibiotics can be purchased without a prescription, 93 (54.1%) thought doctors would prescribe the same medication. Residence (rural), sex (male), educational status (diploma and degree holder), and occupation (farmer) were statistically significant.
Barber, D.A.; *et al.* 2017 [60]<br><br>Philippines<br><br>Central Visayan region	n= 307 men, women, and transgender from five low-income community areas including churches, schools, and health centres. In addition, people from two communities visiting the sari - sari stands selling drugs n=106	Socioeconomic deprivation, age, sex, education, household size.	Sharing antibiotics.	Proxy: antibiotic use.	a) Identify sociodemographic, knowledge and attitudinal correlates to antibiotic sharing b) explore community informal distribution of antibiotics from roadside stands. Quantitative cross-sectional studies. Study 1- Liaison with non-governmental organisations (NGO) and community leaders re selection of community venues. 46 item survey self-administered to eliminate bias. Eight sessions over five weeks. Study 2 -n=106 roadside stands selling antibiotics assessed. Logistic regression was used to assess the univariate and multivariable correlations.	278 surveys completed with a mean age of 32. 57 % of participants were female. 35% of the study population were unemployed. Prevalence of antibiotic sharing 78% most often with family members. Antibiotic knowledge moderate. 70% not aware about developing antibiotic resistance. In multivariable analysis, agreement that it is safe to prematurely stop a course of antibiotics and concerns about antibiotic side effects were significantly associated with antibiotic sharing. However, it was not associated with sociodemographic factors or antibiotic knowledge. Out of 107 sari- sari stands 64 (60%) sold antibiotics. 56 (59%) out of 95 sets of antibiotics had no expiry date. Those with an expiry date 10 % had already expired.

Author, Year, Country, region and reference	Population sample (n)	Indicators of the social determinants of health included as data	Evidence of health seeking behaviour	Infection related data	Study design and aim	Key findings
Chowdhury, M.; *et al.* 2019 [61]<br><br>Bangladesh.<br><br>Chandpur	n = 59 participants (25 female and 34 male) from 15 different rural villages.	Age, sex, education and occupation, home building material, water and sanitation, income sources and transportation.	Access for antibiotics.	Proxy: antibiotics.	To explore socio-cultural factors and practices for access and use of antibiotics and understanding of antimicrobial resistance. Qualitative study, second stage of the Antibiotic Access and Use (ABACUS) study. Residents identified in the Matlab Health and Demographic Surveillance System (HDSS) database and randomly sampled. Interviews were held in people's homes and the focus group discussions (FGD) were held in village community spaces. Levesque's Access Framework used for thematic analysis.	Participants access formal and informal services with treatment seeking behaviour affected by social and cultural beliefs, previous experience and perceptions about factors identified in the theoretical framework. Islamic spiritual practices are important local healing methods including Jharfuk (sorcery involving blowing holy verses), Kabirajiâ (treatment with herbal extracts), Tabijâ (an amulet containing verses from holy books believed to be protective and curative against diseases) and Pani Pora (water with spell from the religious leader believed to be curative). Seeking conventional healthcare affected by ease of access. Men complained about the distance to hospitals. Structural violence in hospitals hindered access for antibiotics. Pharmacies and clinics preferred sources.
Mainous lll, A. G.; *et al.* 2008 [62]<br><br>United States of America (USA) Charleston, South Carolina	n= 28 Latino migrant men and women in an urban area.	Age, sex, migrants, education status.	Antibiotic self-medication.	Proxy: antibiotics.	Qualitative study using three focus groups to assess experiences of acquiring and using antibiotics as treatment for infections. Flyers used and participants self-identified for study. Focus groups conducted in Spanish.	Themes identified included: a) previous experience affected risks of self-medication with antibiotics, - self-diagnosis and self-treatment if past history of similar illness including injections b) the need to see a physician to obtain antibiotics- concern about costs and language barriers. If illness an emergency / serious would consult a doctor but may contact practitioner in Mexico. c) physician visits for the diagnosis and treatment of children prioritised; d) informal strategies to obtain antibiotics without a prescription included relatives sending antibiotics from Mexico, obtaining medication illegally from local Latino stores; e) risks of self-medicating with antibiotics - very limited awareness. Preferred messaging - need to go to the doctor for a consultation and not treat themselves.

Author, Year, Country, region and reference	Population sample (n)	Indicators of the social determinants of health included as data	Evidence of health seeking behaviour	Infection related data	Study design and aim	Key findings
Mishra, S.; *et al.* 2020 [63]<br><br>Nepal<br><br>Rolpa District	n= 720 Nepalese men and women from a rural community.	Socioeconomic deprivation, age, sex, education. Religion including Hindu Buddhist and Christian. Marital status, family types including extended family. Average income per month. Decisions for treatment, health insurance.	Self-medication practice.	Proxy antibiotics.	The purpose of this study was to assess self-medication practice and its influencing factors. Quantitative cross-sectional study. A trained research assistant conducted face-to-face interviews. The questionnaires constructed from concept of model comprised of a) general characteristics (11 items), b) perception of self-medication (15 items), c) access to health service (30 items), d) social support (13 items), e) self-medication practice (19 items).	54.6% of the participants regularly self-medicated with the remaining 45.4% sometimes undertaking the practice. Reasons included saving time, keeping medicine obtained from family for an emergency and the distance between home and hospital facilities. Statistically significant factors included being female, over 49 years old, non-Hindu, extended family, middle/high income, decision making by others and having National Health Insurance.
Nabaweesi, I.; *et al*. 2021 [64]<br><br>Uganda<br><br>Kampala	n= 279 men and women attending the urban Kiruddu National Referral Hospital either as an outpatient or inpatient.	Age, sex, education status, employment status, occupation and religion.	Antibiotic practices and self-medication.	Proxy: antibiotics.	To assess the perceptions and practices of antibiotic misuse including self-medication among patients seeking healthcare. Quantitative cross-sectional study was conducted. The sample size was calculated using Kish Leslie formula. Interviewers received training and administered the pre-tested questionnaire. Assessment was on use and misuse of medication. Analysis was undertaken using STATA software.	212 (76%) participants had taken antibiotics in the previous 6 months. 60.4% had taken for bacterial infections, 17% had used antibiotics for parasitic infestations and 12.7% for non-communicable diseases. Factors associated with antibiotic misuse included people attending outpatients, being female, youths aged 18 to 35 years, unemployment, and low educational achievement. Approximately one-third of participants did not complete the course of antibiotics. Reasons included feeling better, lack of money to purchase medication and side effects were cited.<br>Factors associated with self-medication included male participants who were twice as likely to take medications without prescriptions and Muslims who were significantly more likely to self-medicate.
Om C.; *et al.* 2017 [65]<br><br>Cambodia	n= 35 family members of inpatients, n= 7 untrained pharmacy	Socioeconomic deprivation.	Antibiotic seeking behaviour.	Proxy: antibiotics.	Explored the antibiotic-seeking behaviour and drivers of antibiotic misuse. Qualitative study using in-depth interviews and focus groups. A purposeful selection of family	Widespread misuse of antibiotics in the community due to antibiotic-seeking behaviours with unrestricted access and poor knowledge about antibiotics. Reasons for self-medication included: convenience, balancing earning an income, caring for children, the elderly and

Author, Year, Country, region and reference	Population sample (n)	Indicators of the social determinants of health included as data	Evidence of health seeking behaviour	Infection related data	Study design and aim	Key findings
Khmer	attendants, n= 3 trained pharmacists and n= 30 nurses.				members of patients admitted to public hospitals was used to recruit participants for individual interviews.	providing care to relatives in hospital, cost of treatment and medicine, trust in the healthcare provider and effectiveness of treatment. Unrestricted access to antibiotics is facilitated by community actors including both trained and untrained healthcare providers, shopkeepers, and grocery shops in remote areas. All actors had poor knowledge about the indications and uses of antibiotics. Uses included, minor wounds, injuries, cuts, inflammation.
Saradamma, R.D.; *et al.* 2000 [66]<br><br>India<br><br>Trivandrum City, Kerala	n = 400 for household surveys and n=405 for pharmacy surveys in a peri-urban community primary care clinic.	Age, ethnicity, sex, socioeconomic deprivation, education status, employment status, medical insurance benefits from employment.	Antibiotic seeking behaviour.	Respiratory tract infection.	Aim to investigate the incidence of self-medication with antibiotics and the factors influencing this practice. Cross- sectional study with initially a random sample of households which were surveyed in one primary health centre. Followed by pharmacy-based interviews and observation. Data were collected from antibiotic purchasers sampled from 11 out of the 12 private pharmacies in the area.	By combining the household survey and pharmacy observations, it was estimated that almost four people per 1000, is engaged in self-medication using antibiotics in any two-week period. People least likely to follow this practice are from higher income families, having more education and higher status occupations and having medical insurance. Conversely, logistic regression analysis indicated that risk of buying antibiotics without a script was associated with education at the secondary level or below, the perception that it is expensive to consult a doctor and low satisfaction with medical practitioners.

Table 4: Included studies addressing general aspects of health seeking behaviour in relation to AMR

Author, Year, Country, region and reference	Population sample (n)	Indicators of the social determinants of health included as data	Evidence of health seeking behaviour	Infection related data	Study design and aims	Key findings
Anstey Watkins, J.; *et al.* 2019 [67]<br><br>South Africa Agincourt, Ehlanzeni District of Mpumalanga	n= 26 men and n=34 women caring for children both under and over five years old from rural villages.	Age, sex. socioeconomic deprivation, employment most unemployed or pensioners. Traditional healer.	Antibiotic access and use.	Proxy: antibiotics.	Investigated where community members are accessing and sourcing healthcare treatment and antibiotics. Explored understandings and experiences of antibiotics and antibiotic resistance. Qualitative cross-sectional study. Exploratory study subset of the Antibiotic Access and Use (ABACUS) project under the International Network for the Demographic Evaluation of Populations and their Health Network, conducted within six LMICs. In depth interviews- sample standardized and stratified for comparison with ABACUS sites. Interviews conducted in homes. Six focus groups assisted by Community Advisory Group and permission from village chief. N Vivo – (qualitative software) thematic analysis.	Most of the respondents used the government, public health system. Traditional healers and traditional medicines also combined with biomedical treatments. Large variety of other sources used to obtain antibiotics e.g., door to door sellers, church pastors, markets. Participants knew where to access but limitations due to cost and transport. Mixed understandings about antibiotics - most not heard of antibiotics. Evidence of not taking the full course, evidence of sharing antibiotics, unsure whether medication purchased was antibiotics due to packaging and mixed together and sold as nutritional supplements or analgesics. Results improved if previous treatment for Tuberculosis (TB), Human immunodeficiency virus (HIV), acquired immunodeficiency syndrome (AIDS).

Author, Year, Country, region and reference	Population sample (n)	Indicators of the social determinants of health included as data	Evidence of health seeking behaviour	Infection related data	Study design and aims	Key findings
Do N.T.T.; *et al.* 2021 [68]<br><br>Bangladesh, Ghana, South Africa, Thailand, Vietnam, Mozambique Kintampo, Dodowa, Manhica Agincourt, Matlab, Kanchanaburi Filabavi	n= 8214 customers and n= 6190 rural households	Sex, age, number of people in the household, highest education level in the family. Health care coverage, healthcare options available to the household. Environment - drug suppliers, information sources.	Antibiotic access and treatment. Factors determining choice of drug supplier convenience, trust, cost, other factors.	Proxy: antibiotics for multiple conditions.	Comparison of community-based antibiotic access and use practices across communities in Lower middle-income countries LMICs to identify contextually specific targets for interventions to improve antibiotic use practices. Multi modal approach using both quantitative and qualitative methods. Part 1 mapping of suppliers and inventories, part 2 preparatory in-depth interviews and focus group discussions with suppliers and community members. Part 3 quantitative longitudinal household surveys of Health and Demographic Surveillance Sites. Simultaneously, community antibiotic supply was quantified through standardised customer exit interviews. Part 4, to explain any result discrepancies - explanatory in-depth interviews and focus group discussions conducted.	8214 exit interviews were undertaken at 140 suppliers. Antibiotics dispensed without prescription were greater in the low-income (LIC) and LMIC Ghana (1168 [36·1%] of 3237, Bangladesh 844 [45·7%] of 1859, Vietnam 773 [55·2%] of 1399 compared with upper middle-income countries (UMIC) Thailand (18 [3·9%] of 462 South Africa five [1·2%]. The exception was Mozambique 67 [8·0%] of 839. Self-medication was considered less time consuming, cheaper, and overall, more convenient than access via healthcare facilities. Factors involved relevant policies, trust in the supplier and the drug, disease severity, and whether the antibiotic was intended for a child. Confusion regarding how to identify oral antibiotics was identified in both Africa and Asia.

Author, Year, Country, region and reference	Population sample (n)	Indicators of the social determinants of health included as data	Evidence of health seeking behaviour	Infection related data	Study design and aims	Key findings
Essigmann, H. T; *et al.* 2022 [69]<br><br>United States of America (USA) and Mexico<br><br>Starr Country, Texas	n= 616 USA urban border residents of any ethnicity.	Age, sex, socioeconomic deprivation, education status, employment status, marital status.	Sourcing antibiotics.	Proxy: antibiotic use.	To determine social, cultural, and clinical features associated with cross-border procurement. Quantitative study, data collected during the baseline examination of a longitudinal cohort study. Participants self-reported the name, date of use, and the source country of each antibiotic used in the past 12 months. Questionnaires used to collect and assess socioeconomic status.	274 participants (44.48%) had taken one or more antibiotics in the previous 12 months with 63.5% (174/274) sourcing from Mexico. Females (48.9%) were more likely to use antibiotics than males (33.3 %). Health insurance status was strongly associated with the country used for obtaining antibiotics. People without insurance, part-time, no employment or greater acculturation with Mexican culture were more likely to acquire antibiotics in Mexico. However, factors not statistically significant included being retired, on leave, or disabled were significantly less likely to cross the border.

Author, Year, Country, region and reference	Population sample (n)	Indicators of the social determinants of health included as data	Evidence of health seeking behaviour	Infection related data	Study design and aims	Key findings
Haenssgen, M. J.; *et al.* 2020 [70]<br><br>Laos, Thailand Chiang Rai province, Salavan province, Lao PDR	Data from five rural villages including n=1421 illness episodes from n=2066 villagers. Chaing Rai n=625 Salavan n=796	Sex, age, socioeconomic deprivation, ethnicity, education status, employment status, housing, size of household, environment including work environment, religion, culture, healthcare facilities. Marginalisation evaluated in terms of education, wealth, and ethnicity. The respondent had not received any formal education, belonged to the lowest household asset quintile, did not belong to the majority Thai/Lao Loum ethnic groups.	Assessment of precarity affecting health seeking behaviour.	Respiratory tract infection, upper respiratory tract infection, sepsis. Survey of multiple illnesses.	Assessed a) the impact of precarity, marginalisation and clinical presentation on healthcare-seeking behaviour b) assessed whether patients subjected to precarious livelihoods have clinically less advisable healthcare-seeking behaviour. Quantitative study using two-round individual-level census surveys over a three-month period. Household face to face 45-minute survey. Analysis included descriptive statistical and multivariate logistic regression analysis for the level of illness.	Patients in precarious circumstances were up to 44.9 percentage points more likely to misuse antibiotics in the presence of situational facilitators (predicted antibiotic misuse: 6.2% (95% CI: 0.9% to 11.4%) vs 51.1% (95% CI: 16.6% to 85.5%) for precarious circumstances with/without facilitation). Marginalisation was linked to lower antibiotic use, but this did not translate into clinically more advisable behaviour. Clinical presentation was only a minor point in determining healthcare access and antibiotic use.

Author, Year, Country, region and reference	Population sample (n)	Indicators of the social determinants of health included as data	Evidence of health seeking behaviour	Infection related data	Study design and aims	Key findings
Kamenshchikova, A.; *et al.* 2018 [71]<br><br>Netherlands	n= 17<br>Public health nurses x2<br>Microbiologists x5<br>Public lay physicians x4<br>Syrian refugees x6 in an urban community venue.	Socioeconomic deprivation, age, sex.	Assessment of views about screening refugees.	General review about acceptability of screening for antimicrobial resistance (AMR).	To understand how different stakeholders view screening of non-hospitalised refugees. Qualitative study including four stakeholder groups already working with refugees. Methods included observations of participants, in depth interviews, group interviews and informal discussions.<br>Refugees recruited via Syrian Physician. Number of participants dictated by data saturation. Interviews in English. Preparatory work undertaken to build trust.	1. Microbiologists and the public health physicians supported screening of healthy refugees for Public Health and research purposes.<br>2. Nurses - personal and contextualised views. Risk of causing refugees to worry about their health status and if tested cannot treat them. Lack of general knowledge about AMR can cause hostility and stigma as being different to the other populations.<br>3. Refugees - Concerns re a) stigma b) the media being involved and associated risks c) lack of personal benefits even if screened and in some cases no treatment for resistant bacteria.<br>Refugees viewed antibiotics positively as they perceive antibiotics can cure other problems e.g., pain.<br>Expressed differences in obtaining antibiotics i.e., from pharmacies and voiced their concerns about the regulated system for obtaining antibiotics.
Khare, S.; *et al.* 2021 [72]<br><br>India<br><br>Ujjain	n= 270 Children under five and their respective caregivers based in rural villages.	Childs age and sex. Mothers age, socioeconomic status, education status, occupation, parity of mother. Household size.	Determinants and pathways for children with infections seeking antibiotics.	Respiratory tract infection (RTI), skin infection e.g., Impetigo. Gastrointestinal infections.	To explore the health care seeking pathways of the care givers, antibiotic prescribing and determine the socio demographic factors associated with the population. Quantitative, cohort study including twice weekly visits for over 113 weeks to complete observational diaries.<br>Outcome measures included: first point of care, healthcare-seeking pathway and antibiotic prescribing. Health seeking behaviours were determined using mixed-	RTI were the most prevalent infection (69%). Caregivers did not seek treatment for 33% of episodes, mostly RTIs. Informal healthcare providers (49% of illness episodes) were the most frequent health care seeking pathway. The adjusted relative risk for obtaining no treatment, home treatment and treatment by informal healthcare providers was higher for RTIs (aRR=11.54, 1.82 and 1.29, respectively), illiterate mothers (aRR=2.86, 2.38 and 1.93, respectively), and mothers who were homemakers (aRR=2.90, 4.17 and 2.10, respectively). Socioeconomic status was associated with health seeking behaviour, with the highest aRR for no treatment in the lowest two socioeconomic quintiles (aRR=6.59 and 6.39, respectively). Antibiotics were prescribed in 46% (n=670/1450) illness episodes.

Author, Year, Country, region and reference	Population sample (n)	Indicators of the social determinants of health included as data	Evidence of health seeking behaviour	Infection related data	Study design and aims	Key findings
					effects multinomial logistic regression.	

Author, Year, Country, region and reference	Population sample (n)	Indicators of the social determinants of health included as data	Evidence of health seeking behaviour	Infection related data	Study design and aims	Key findings
Kleinert E.; *et al.* 2021 [73]<br><br>Germany<br><br>Celle and Friedland	Refugee centres: First cohort: n= 1017 refugee patients (with a total of 2282 encounters). Second cohort: n=2238 refugee patients who had 4094 encounters in total. Refugees from Syria, Iraq and Afghanistan.	Age, sex (female), residence permit.	Obtaining prescribed antibiotics in refugee centres.	Upper respiratory tract infection (URTI) and urinary tract infection (UTI).	To provide an overview of antibiotic prescription behaviour including diagnoses and demographic characteristics. Quantitative cohort study. The first cohort included participants who sought medical care at a temporary shelter built to accommodate the refugee crisis in 2015. The second cohort involved participants at a reception facility in Friedland between August 2017 and August 2018. Data including demographic data, International Classification of Diseases ICD-10 codes and prescribed antibiotics were extracted from either electronic or paper records.	11% of consultations resulted in antibiotic prescription. 19% (n = 624) of all patients were prescribed at least one course of antibiotics. Prescriptions were most prevalent in children under the age of 10 years (n= 209, 24%) and patients over the age of 70 years (n = 7, 24%). Tonsillitis, acute bronchitis, URTI, UTI, and otitis were the most common diagnoses with antibiotic prescriptions. For URTI (n = 95), 8% of patients received antibiotics. The very low prescribing rate was consistent with the guidelines however, acute bronchitis was treated with antibiotics in 74% of cases, which was contrary to guideline advice. Patients with UTI were treated with antibiotics in 73% of cases, mostly with sulfamethoxazole /trimethoprim and ciprofloxacin, neither was recommended in the guidelines.

Author, Year, Country, region and reference	Population sample (n)	Indicators of the social determinants of health included as data	Evidence of health seeking behaviour	Infection related data	Study design and aims	Key findings
Lucas, P.J.; *et al*. 2019 [74]<br><br>Bangladesh Gazipur District, Mirzapur District	n= 48 rural and urban indigenous households.	Ethnicity, socioeconomic deprivation, housing, household size, livestock in home, culture, proximity to healthcare resources.	Access to antibiotics	Proxy: acquiring antibiotics	To explore how households in Bangladesh were accessing antimicrobials for themselves and their domestic animals. Qualitative study using in depth household interviews. Purposively sampled to include households with lower income, children under five, older adults, minority groups and keeping livestock at home.	Antibiotics were obtained from drug shops, government and charitable hospitals, community /family planning clinics (rural area only), private and specialised hospitals. Low socio-economic households predominantly used government and charity locations. Cost prevented purchasing full courses of antibiotics. Multiple and incomplete courses were common even when prescribed by a qualified doctor. Male participants mainly made decisions about health care treatment seeking and purchase of medicines. Decisions were made independently or with other adults including, brothers, neighbours, and their mother. Women frequently deferred to the decisions of others including husbands, brother-in-law, mother-in-law, or older sons even if this caused delays. People's knowledge and understanding of antibiotics was categorised as knowledge by name, little or no knowledge, powerful medicines, and knowledge of action. Antibiotics were identified by their high cost compared to other medicines.

Author, Year, Country, region and reference	Population sample (n)	Indicators of the social determinants of health included as data	Evidence of health seeking behaviour	Infection related data	Study design and aims	Key findings
Miller M. F.; *et al.* 2022 [75]<br><br>United Kingdom<br><br>England<br><br>Bradford	Urban singleton children under 2 years old n= 2493.<br>Follow up stage at 24-month n= 2002 (80.3%).	Socioeconomic status, sex of infant, ethnicity, number of people in household.<br>Household mould/damp, gas cooking. Quartile of Particulate Matter 25 in relation to Bradford level.<br>Additional data re the child e.g., delivery, presence of congenital abnormalities.	Obtaining antibiotics.	Respiratory tract infection (RTI), upper respiratory tract infection (URTI), lower respiratory infection (LRTI).	Establish child, family, and environmental factors that were associated with prescribing of amoxicillin. Findings to inform antimicrobial stewardship for children. Quantitative cohort study, including assessing the variation in amoxicillin prescribing at GP surgery-level. Outcome variables were at least one amoxicillin prescription from primary care during the first and second year of life and at least one primary care consultation for upper or lower RTIs.	48.9% of all mothers had a Pakistani ethnic background (n = 1220/2493) and 43.7% were from the two most deprived socioeconomic groups (n = 1089/2493). A total of 1594/2493 children (63.9%) received amoxicillin prescription during the first 2 years of life. The odds of receiving at least one prescription for amoxicillin during year 1 was higher for children with mothers from a Pakistani ethnic background, regardless of their country of birth compared to children of White British mothers. There were associations between the odds of having at least one URTI. GP consultation and ethnic background and breastfeeding duration during years 1 and 2. For LRTIs, significant associations were seen between GP attendance and sex, ethnicity, and socioeconomic status during year 1, and ethnicity, socioeconomic status, congenital anomalies, delivery mode, and childcare attendance during year two.
Rahill, G. J.; *et al.* 2012 [76]<br><br>United States of America (USA)<br><br>Miami-Dade County, Florida	n=10 picuristes Haitian immigrants providing injections on demand) to 25 clients over 18 years old with at least one Haitian parent.	Ethnicity, culture	Obtaining antibiotics via injection.	Infections including skin / wound infection. septicaemia	Mixed methods study to investigate the experiences of picuristes and the people seeking treatment via injection.<br>Semi-structured, face-to face interviews conducted in various settings. Interviews conducted in English and Haitian facilitated in-depth information about the types and frequency of substances injected. Interviews transcribed and analysed using ATLAS.ti and SPSS software packages.	Injections from picuristes are a hidden health practice among some immigrants. Injections based on clients' symptoms however, there are no treatment protocols. Findings indicated frequent use and misuse of antibiotics. 70% of the picuristes reported routine injections of broad-spectrum antibiotics including penicillin, ampicillin, tetracycline, and streptomycin. The clients and the picuristes share the same supernatural beliefs.

Evidence for primary question B- What evidence is there on the burden and impact of AMR in different populations disaggregated by the socio - economic and demographic variables, e.g., race, ethnicity, social deprivation, and gender?

Table 5: Included studies addressing the prevalence of AMR in vulnerable populations

Author, Year, Title, Country, region and reference	Population sample	Indicators of social determinants of health included as data	Evidence of health seeking behaviour	Infection related data	Study design and aim	Key findings
Albanese, B.A.; *et al.* 2002 [77]<br><br>United States of America (USA) Baltimore	Surveillance data from the urban Baltimore metropolitan area (BMA). Included men, women and children n= 2094. Racial groups: White, Black, Other.	Age, racial group / ethnicity, region	Attended urban acute care hospital.	Penicillin-Resistant *Streptococcus pneumoniae* accounting for bacteraemia; pneumonia, otitis media and other infections.	Outlined the epidemiology of penicillin-resistant *Streptococcus pneumoniae* (PRSP) in the designated area from 1995 to 1997. Identify geographic, demographic, and associated risk factors. Quantitative; prevalence study. Blood and cerebrospinal specimens from invasive active surveillance programme. Hospital infection teams collected demographic, clinical and isolate information for positive specimens. Univariate and multivariate logistic regression analysis was performed.	In the study period the proportion of pneumococcal isolates resistant to penicillin increased 42%, from 5.7% to 8.1% of cases. PRSP rates were highest in children under 5 and adults over 65, Black patients and people living in an urban environment. However, logistic regression showed the proportion of PRSP cases was higher in White people (10%) than Black people (5%) and suburban counties (10%) versus urban counties (6%). A range of multi morbidities were also identified across a range of ages.
Angeletti S.; *et al*. 2016 [78]<br><br>Italy Castelnuovo di Porto near Rome	n= 48 Syrian refugees newly arrived. Males 30/48 (62.4%), female 18/48 (37.5%), median age 20 years. 11/48 (23%) children 'below 14 years. All refugees were Muslim.	Age, sex, religion.	Migrant health surveillance on entry to country.	Rectal, nasal/pharyngeal swabs and serum samples taken for surveillance.	To determine the health status and presence of microorganisms. Quantitative, prevalence study. Newly arrived migrants were accommodated at an asylum centre and screened. Susceptibility tests were conducted on all microorganisms identified.	18/48 refugees did not consent to blood tests for religious reasons. Bacteria isolated from swabs included: unusual gram-negative bacteria species e.g., *Pseudomonas putida*, *Pseudomonas monteilii*, *Pseudomonas fulva*, *Pseudomonas moselii*, *Aeromonas veronii*, *Aeromonas caviae*, *Aeromonas hydrophila*, *Acinteobacter guilloviae*, *Acinteobacter lowffii; Acinetobacter johnsonii; Acinteobacter tjernbergae; Pantoea agglomerans; Pantoea calida*. Among isolates, strains resistant to carbapenems, ESBL producers and methicillin resistant were present.

Author, Year, Title, Country, region and reference	Population sample	Indicators of social determinants of health included as data	Evidence of health seeking behaviour	Infection related data	Study design and aim	Key findings
Aro, T.; *et al*. 2018 [79]<br><br>Finland Helsinki	Data from asylum seekers and refugees admitted to Helsinki University Hospital. n=447	Age, sex, country of origin, co-morbidities (Charlson Comorbidity score).	Screening for methicillin-resistant *Staphylococcus aureus* (MRSA) and multi resistant gram-negative (MRGN) bacteria following admission to a tertiary hospital.	Patients admitted with a range of diagnoses. Infectious diseases 83/447 included respiratory tract infection; urinary tract infection; skin infections, acute gastroenteritis. Other conditions included non-infectious diseases and pregnancy related 133/447.	Quantitative prevalence study to investigate the prevalence of multi drug resistant bacteria and identify people at risk of colonisation. All attendees screened for MRSA, vancomycin-resistant extended-spectrum *Enterococcus* (VRE), extended-spectrum beta-lactamase-producing Enterobacteriaceae (ESBL-PE), Carbapenemase-producing Enterobacteriaceae (CPE) multi resistant *Acinetobacter baumannii* (MRAB) and multiresistant *Pseudomonas aeruginosa* (MRPA). Screening included two sets of samples, ideally collected on consecutive days. Demographic data and Charlson Comorbidity Index collected.	Patients from Iraq (46.5%), Afghanistan (10.3%), Syria (9.6%), and Somalia (6.9%). 45.0% of patients (201/447) were colonised by multi drug resistance (MDR) bacteria: 32.9% had ESBL-PE, 21.3% MRSA, 0.7% CPE, 0.4% MRPA; 0.4%MRAB). 12.5% of patients had two or more MDR bacteria strains. MDR bacterial carriers was highest in patients from Iraq and Syria (57.2% and 55.8% respectively), Afghanistan (34.8%) and Somalia (25.8%). 10 of the MDR bacterial carriers (5.0%; 10/201) had a clinical MDR bacterial infection, with wound infections and urinary tract infections (UTI) as the most common presentation. Multivariable analysis showed independent risk factors for colonisation with ESBL-PE as geographical region of origin, age under 6 years, short time from arrival to first sample and prior hospitalisation abroad. MRSA colonisation included geographical region and prior surgery outside Nordic countries as risk factors.
Barger, S.D.; *et al*. 2020 [80]<br><br>United States of America (USA) Yuma County, in Southwestern Arizona	Men and women from an underserved Hispanic border urban community n=613 including non-Hispanic White and Hispanic White. Public and private venues.	Age, race / ethnicity, marital status, education status, home ownership.	Screening	*Staphylococcus aureus (S. aureus)* colonisation assessment	Quantitative prevalence study to evaluate hypotheses generated by fundamental cause theory regarding the socioeconomic status gradient in colonisation with *(S. aureus).* Participants recruited in small groups based on a theoretical sampling frame. Data collected over a 2-year period. Sampling kits and information about swab handling and swabbing methods provided.	34.4% of participants were colonised with *S. aureus*. Colonisation among non-Hispanic participants was nominally higher (39.0%) than among Hispanics (31.3%). Education was not associated with colonisation in the full sample or Hispanics or non-Hispanic Whites with or without adjustment for age and sex. Male sex was the only variable consistently associated with *S. aureus* colonisation. *S. aureus* colonisation was not associated with ethnicity or educational attainment and may be outside the influence of socioeconomic status-based resources.

Author, Year, Title, Country, region and reference	Population sample	Indicators of social determinants of health included as data	Evidence of health seeking behaviour	Infection related data	Study design and aim	Key findings
Charlebois, E.D.; *et al.* 2002 [81]<br><br>United States of America (USA)<br>San Francisco	Men and women of mixed ethnicity including White, Black and mixed from an urban poor environment. n=833	Sex, age, race, homeless, injection drug use, socioeconomic deprivation, use of healthcare resources.	Screening for colonisation	Prevalence and colonisation of methicillin-Resistant *Staphylococcus aureus* (MRSA)	Quantitative prevalence study investigating the prevalence and risk factors for nasal colonisation with MRSA. Compared antibiotic resistance patterns and genetic similarity between community samples of *S. aureus* and health care-identified MRSA isolates.	60.2% (n = 500) of the sample were "true homeless" and (n = 331) were recruited from hotels. 190 (22.8%) were nasally colonised with *S. aureus*. Of those colonised with *S. aureus*, 12% were resistant to methicillin. White individuals were significantly more likely to be colonised with *S. aureus* than Black or those of other or mixed race (27.7% vs. 19.0% P =.0034). Colonisation with *S. aureus* did not vary significantly by gender or homelessness however, people age >50 years and a history of drug abuse were more likely to be colonised.
Chattopadhya D.; *et al.* 2020 [82]<br><br>India<br>Delhi,<br>Gurgaon	Urban migrant male and female labourers in a peri urban setting. A total of n=136 and n=124 labourers belonging to migrant labourers (ML) and non-migrant labourers (NML) subgroups, respectively.	Ethnicity, age, sex, socioeconomic deprivation, education status, employment, work environment, housing and home environment including water, sanitation, hygiene (WASH).	Screening to assess AMR	AMR based on intestinal carriage rate of extended spectrum beta-lactamase (ESBL), carbapenemase and New Delhi metallo-beta-lactamase type-1 (NDM-1) in *Escherichia coli* (*E. coli)* as indicator organism	To assess the level of antimicrobial (AMR) based on intestinal carriage rate of ESBL, carbapenemase and NDM-1 in *E. Coli* as indicator organism in study population employed as unskilled labourers for civil constructions. Investigate potential evidence for transmission of AMR from the urban migrant labourers to labourers from local resident rural community employed for the same role, sharing residential premises and civil facilities as urban migrant labourers during the employment period.	The ML group had a low literacy level, poor accommodation - makeshift tents, practiced open defaecation and had poor drinking water. There was a higher frequency of chest infections and diarrhoea and intestinal carriage rate of *E. coli* with AMR compared to the NML group. Treatment seeking behaviour varied by group. Practice of self-medication was more prevalent in the ML group compared to the NML group. Left over medicines were used by both groups. Non-completion of medication was also evident and was higher in the ML group. On follow-up, no significant difference in the faecal carriage rate of ESBL-EC between both the groups. The NML group showed an increase in the prevalence of carbapenem resistance with NDM-1 production during the period of co-inhabitation with urban migrant labourers.

Author, Year, Title, Country, region and reference	Population sample	Indicators of social determinants of health included as data	Evidence of health seeking behaviour	Infection related data	Study design and aim	Key findings
Conceição, T.; *et al.* 2019 [83]<br><br>Portugal, Lisbon	Homeless men and women n=84 in urban areas.	Age, sex, housing, co-morbidities including mental health.	Assess nasal carriage and colonisation	*Staphylococcus aureus (S. aureus)* nasal carriage.	The aim of the study was to determine methicillin resistant *Staphylococcus aureus* (MRSA) colonisation rates among the homeless in Lisbon, determine major risk factors for *S. aureus* carriage, and characterise *S. aureus* clonal population, antibiotic resistance, and virulence.	A total of 84 homeless people (34 with no permanent address and 50 living in a shelter) were nasally screened 43 (51.2%) were *S. aureus* carriers. MRSA was detected in a single individual. The incidence of *S. aureus* carriage in individuals with no permanent address was higher than among people living in shelters however, it was not statistically significant. A high proportion carried the highly transmissible ST398-t1451 MSSA lineage. *S. aureus* carriage was higher among younger individuals (45.7 ± 12.7 versus 52.5 ± 10.8 years, p = 0.009) and people with asthma (9% versus 0%, p = 0.04). However, there was no significant difference between male and female individuals (50.8%, n = 32 and 52.4%, n = 11 respectively, p = 0.90), or in other conditions associated with increased MRSA carriage.
Cooke F.J.; *et al.* 2008 [84]<br><br>United Kingdom, England, Cambridge	Homeless men and women n= 284 patient episodes attending a General Practice (GP) in an urban area.	Age, housing homelessness, drug use.	Treatment for skin and soft tissue wound infection in GP clinics.	Skin and soft tissue wound infection	Quantitative cohort study assessing the presence of methicillin resistant *Staphylococcus aureus* (MRSA) in skin and soft tissue infections. Specialist General Practitioner surgery caring for the homeless. Four-year study (3 August 2003 to 3 August 2007).	*Staphylococcus aureus S. aureus* was cultured from 299 of 641 (46.7%) wound swab (284 patient episodes). Sites included lower limb/groin (41.9%); the upper limb (20.8%), head/neck (18.7%), skin (site unspecified 8.1%), genitals/sacrum (3.2%) and other (7.4%). Age range 17 to 72 years (median: 30 years). 284 *S. aureus* infections, 85 (29%) were due to MRSA. Patients with MRSA were younger than those with methicillin susceptible *S. aureus* (MSSA) (median: 30 vs 36 years). Query due to the local intravenous drug users (IDU), 80% (68/ 85) MRSA infections were from known IDUs. 87.1% of MRSA infections were identified as being community based.

Author, Year, Title, Country, region and reference	Population sample	Indicators of social determinants of health included as data	Evidence of health seeking behaviour	Infection related data	Study design and aim	Key findings
Dinh, A.; *et al*. 2016 [85]<br><br>France<br>Paris	Paraplegic and tetraplegic men and women > 16 years n= 318 episodes in 256 in patients at a secondary care hospital.	Age, sex. Disability- mean duration of disability.	Spinal cord injured patients post trauma.	Blood stream infections (BSI) due to multidrug-resistant organisms.	Quantitative cohort study to examine the prevalence of multidrug-resistant organisms (MDRO) during blood stream infections (BSI) in patients with spinal cord injury. Comparative study of the characteristics of the MDRO population with the non-MDRO population. Severity defined as the requirement of at least one of the criteria: volume expansion required, assisted (mechanical) ventilation, vasopressor requirement and ICU (intensive care unit) admission during the episode.	A total of 318 BSIs from 256 patients were analysed. Primary sites included urinary tract infection (34.0%), pressure sore (25.2%) and catheter line-associated bloodstream infection (11.3%). MDROs were responsible for 41.8% of BSIs, however the prevalence remained stable over 16 years. No significant factors for MDRO BSI could be identified concerning sociodemographic and clinical characteristics, primary site of infection and bacterial species in univariate and multivariate analyses.
Eiset, A.H.; *et al*. 2020 [86]<br><br>Denmark, National study.	Syrian asylum seekers n= 113 at an urban asylum centre.	Sex, age, ethnicity, time in Denmark, area of origin, record of child vaccination programme, comorbidities.	Screening at an asylum centre.	Prevalence of methicillin-resistant *Staphylococcus aureus* (MRSA), Giardia, and Blastocystis	Quantitative cross sectional study part of the “Asylum seekers and Refugees’ Changing Health” (ARCH) project. On arrival in Denmark samples were collected for extended-spectrum beta-lactamase-producing Enterobacterales (ESBL-E), carbapenemase producing organisms (CPO), MRSA and *Corynebacterium diphtheriae*. Full medical history and a 20-item questionnaire including socio-demographics, health status, health behaviour and migration history.	Individuals aged between 25 and 37 years old from Aleppo and Damascus agreed to participate. Throat swabs and faecal samples were received from 104 and 48 participants, respectively. Seven individuals (6.7%, 95% CI: 3.3–13.3%) were colonised with MRSA and one with ESBL-E. Three individuals (7.3%, 95% CI: 2.5–19.4%) were colonised with *Giardia intestinalis* and 28 (68.3%, 95% CI: 53.0– 80.4%) with *Blastocystis sp*. (subtypes 1 [n = 5], 2 [n = 9] and 3 [n = 14]). None had CPO or *Corynebacterium diphtheriae* and none reported any gastro-intestinal symptoms. The prevalence of MRSA was high in the asymptomatic population.
Eshetie, S.; *et al*. 2015 [87]<br><br>Ethiopia, Northwestern Ethiopia	Men, women, and children n=442 in the University of Gondar hospital.	Age, sex, education status, housing, rural vs urban.	Symptomatic patients requiring treatment for urinary tract infection (UTI)	UTI with multidrug resistant (MDR) and carbapenemase producing Enterobacteriaceae (CPE).	Quantitative study to determine the prevalence and risk factors of MDR and CPE producing strains among patients with UTI at the hospital.	There were 183 /442 Enterobacteriaceae recovered. Of these isolates, 160 (87.4%) were multidrug resistant Enterobacteriaceae (MDRE).<br>*K. pneumoniae* (95.6%) and *Escherichia coli* (*E. coli)* (92.9%) were found to be the principal MDR isolates. 252 (57.0%) of patients were from rural areas and 286 (64.7%) of study participants had educational level of elementary school and below. The isolates were evaluated for antimicrobial susceptibility, 160 (87.4%, 95% CI; 82 - 92.3%) showed resistance to two or more classes of antibiotics. Bivariate analysis showed age, hospitalisation for the last 12 months, prior urinary

Author, Year, Title, Country, region and reference	Population sample	Indicators of social determinants of health included as data	Evidence of health seeking behaviour	Infection related data	Study design and aim	Key findings
						tract infection for the past 12 months, prior antibiotic use for the past 6 months was associated with MDRE infections. Multivariate logistic regression, independent risk factors for MDRE were prior antibiotic use, and hospitalisation since the past 12 months, age, and sex (female).
Farr, A.M.; *et al*. 2013 [88]<br><br>United States of America (USA)<br>New York	Data set of men, women, and children over 1 year old hospitalised with community-associated methicillin-resistant *Staphylococcus aureus* (CA-MRSA) n=645 and non-*Staphylococcus* n=908,184.	Age, sex, social deprivation, multi morbidities, sexual orientation, environment.	Hospitalisation for CA-MRSA	Respiratory tract infection, skin infection, bacteraemia and unspecified infection included.	Quantitative cross-sectional study to evaluate the influence of neighbourhood factors on CA-MRSA hospitalisations. International Classification of Diseases codes used for data collection (V09.0) and individual variables collated.	CA-MRSA hospitalisations had more than 3 times the odds of having Human immunodeficiency virus (HIV) diagnosis (males: OR 3.3, 95% CI 2.5, 4.3; females: OR 3.1, 95% CI: 1.9, 5.1). After adjusting for individual-level risk factors, females hospitalised with CA-MRSA had more than twice the odds of residing in New York City United Hospital Fund (UHF) neighbourhoods with the highest HIV prevalence compared to the lowest (AORQ4 vs. Q1 2.3, 95% CI 1.2, 2.7). The odds of hospitalisation with CA-MRSA increased with increasing HIV prevalence.
Galindo, G. R.; *et al.* 2012[89]<br><br>United States of America (USA)<br>New York	Men: men who have sex with men (MSM) n=13 Black/African American, Latino/Hispanic White/Caucasian located in an urban area.	Ethno racial, socioeconomic deprivation, age, sex.	Previous treatment for infection	Community associated methicillin resistant *Staphylococcus aureus* (CA MRSA)	Qualitative study examining risk factors that potentially increase transmission. Results to support healthcare professionals in planning potential outbreaks. Previous research highlighted links with Human immunodeficiency virus (HIV) serostatus, methamphetamine use and sexual risk with MSM populations and methicillin resistant *Staphylococcus aureus* (MRSA) infection. Therefore, purposeful sampling was used to ensure inclusive representation.	Two thirds of the participants were engaged in party and play - use of illicit drugs (crystal methamphetamine) while engaging in sexual activity. Participants had multiple partners, and the episodes ranged from a few hours to several days. Knowledge of how men had become infected with CA MRSA ranged from discussing how they became infected while using methamphetamine, to outlining potential sources and not being aware of risks. Many had experienced multiple CA-MRSA infections and had developed self-treatment and prevention strategies. Data from thematic content analysis indicates behaviours and exposures linked with transmission of CA-MRSA are prevalent in certain MSM networks.

Author, Year, Title, Country, region and reference	Population sample	Indicators of social determinants of health included as data	Evidence of health seeking behaviour	Infection related data	Study design and aim	Key findings
Gualandi N.; *et al*. 2018 [90]<br><br>United States of America (USA), Colorado, Connecticut Georgia, Maryland, Minnesota, New York, Oregon, and Tennessee	Data from surveillance of men, women and children in urban and rural areas. n= 45550 cases of invasive Methicillin-resistant *Staphylococcus aureus* (MRSA) infections by race including White,<br>Black or African American, American Indian or Alaska native, Asian, native Hawaiian or other Pacific Islander, or unknown.	Race, age, sex.	Community, hospital, and healthcare related onset of symptoms.	Invasive MRSA infections.	Quantitative, cross sectional prevalence study using surveillance data from 1 January 2005 to 31 December 2014 to describe temporal trends in invasive MRSA both Community associated (CA) and Healthcare associated (HA) by race. Determine whether incidences by race have changed over a time period.	The Emerging Infection Programme sites reported 45550 cases of invasive MRSA infections for the study period including 17225 (38%) in patients of Black race and 25977 (57%) in patients of White race. Epidemiological classes were 9591 (21%) HA onset, 27 041 (59 and 8256 (18%) CA. In 2005, incidence rates for all invasive MRSA cases per 100 000 population were 31.23 for Whites and 79.11 for Black people. In 2013, rates were 19.78 for Whites and 39.93 for Blacks; the unadjusted rate ratios for Black race in 2005 and 2013 were 2.53 and 2.02, respectively. Despite a reduction in healthcare associated MRSA rates there continues to be racial disparities in MRSA rates.

Author, Year, Title, Country, region and reference	Population sample	Indicators of social determinants of health included as data	Evidence of health seeking behaviour	Infection related data	Study design and aim	Key findings
Hewagama S.; *et al.* 2012 [91]<br><br>Australia Central, Australia	Indigenous and non-Indigenous men, women and children admitted to a rural hospital n=125 patients.	Ethnicity, age, sex, multi morbidities, housing, and hygiene facilities.	Patients admitted to hospital with infection	*Staphylococcus aureus (S. aureus)* bacteraemia	Quantitative cross-sectional study to determine whether racial differences exist between racial groups and resistance phenotypes in the clinical manifestations and outcomes of *Staphylococcus aureus* bacteraemia (SAB). Medical records and pathology results reviewed for all patients with positive blood culture for *S. aureus* between 1 January 2003 and 31 December 2006. Data collected included demographic information, comorbidities, clinical presentations, management, and outcomes.	125 patients (indigenous, 111; non-indigenous, 14) were admitted with SAB including 109 adults (indigenous, 95; non- indigenous, 14) and 16 indigenous children (no nonindigenous children were admitted with SAB). Indigenous patients were more likely to reside in remote communities (P < 0.001) and to live within the Alice Springs Region (P = 0.022). Mean annual incidence rate (IR) for adults and children combined were 160.7 per 100 000 Indigenous population and 8.1 per 100 000 non-Indigenous population (incidence rate ratio (IRR) 19.9). Mean annual IR for Indigenous and non-Indigenous adults were 202.9 (95% CI 100.5 -305.4) and 10.2 (95% CI 1.0 -19.5) per 100 000 population respectively (IRR 19.8; 95% CI 4.9 - 42.1). Anti-*staphylococcal* antibiotic was empirically administered to 66 (69.5%) of Indigenous adults, but only five (35.7%) non-Indigenous adults (P = 0.011). After blood culture results were available 12 of 71 (16.9%) Indigenous adults who did not self-discharge and three (21.4%) non-Indigenous adults were not treated with an appropriate antibiotic. Indigenous Australians have the highest reported incidence rate of SAB worldwide reflecting socioeconomic disadvantage and living conditions which do not facilitate adequate hygiene.
Hossain, A., *et al.* 2020 [92]<br><br>Bangladesh, National study	National database with isolate and sensitivity results for men and women n=1663 reports	Urban poor population, age, sex.	Attending healthcare facilities due to infection.	Antibiotic resistance patterns for urinary tract infection caused by *Escherichia coli. (E. Coli)*	Quantitative cross-sectional study designed to explore the age and gender-specific trends of antibiotic resistance for urinary tract infection (UTI) associated with *E. Coli*. A doctor had examined patients to review signs and symptoms and where necessary attended Medinova Medical Services Ltd (MMSL) centres for urine culture and sensitivities. Data was analysed from MMSL outlets. Microbial resistance was examined in Amikacin, Nitrofurantoin, Meropenem, Imipenem and Colistin.	There were 1663 reports of UTI patients with 1285 (77.27%) being females. The subjects' average age was 43.87 - range 6 to 87 years old. Participants sex had a significant effect on the resistance to amikacin, nitrofurantoin, and colistin. Bacteria from males was 2.27 times more resistant to amikacin than isolates obtained from females. Isolates obtained from males were 2.09 times more resistant to colistin and 1.45 times more resistant to nitrofurantoin compared to isolates obtained from females. Isolates obtained from older patients appeared to have high resistance to amikacin and nitrofurantoin. Patients within the age group less than or equal to 18 years were 71% less resistant to amikacin compared to the patients of the age group >60 years.

**Author, Year, Title, Country, region and reference**	**Population sample**	**Indicators of social determinants of health included as data**	**Evidence of health seeking behaviour**	**Infection related data**	**Study design and aim**	**Key findings**
Hota, B.; *et al.* 2007 [93]<br><br>United States of America (USA), Chicago	Men, women and children over one year old. n= 6894 patients attending an urban hospital.	Race, age, sex, socioeconomic deprivation, housing, incarceration, social housing and multi morbidities.	Patients attending hospital for culture of abscess fluid, joint fluid, and bone cultures. Ongoing surveillance.	Community-associated methicillin-resistant *Staphylococcus aureus* (CA-MRSA) skin and soft tissue infections.	Quantitative laboratory-based surveillance nested case control study using CA-MRSA cases compared with community associated methicillin-susceptible *Staphylococcus aureus* (CA-MSSA) controls to estimate rates of infection, impact of overcrowding and geographic distribution of CA-MRSA. Surveillance undertaken from 1 January 2000 to 31 August 2005. Patients were eligible who had been seen in the emergency department or inpatient wards or affiliated clinics and had a positive CA MRSA specimen from one of the following sites- soft tissue, abscess fluid, joint fluid, or bone specimens.	Geographic analysis 4 clusters of CA-MRSA infections one cluster had five high-rise public housing complexes and 8 (18.2%) of 44 patients were within the cluster. However, no association between case status and overcrowding. There was no association between high-occupancy block groups and CA-MRSA infection. There was no association when occupancy was stratified by race/ethnicity or by year of culture. The incidence of CA-MRSA skin and soft tissue infections increased from 24.0 cases per 100 000 people in 2000 to 164.2 cases per 100 000 people in 2005 (relative risk, 6.84 [2005 vs 2000]). Risk factors were incarceration (odds ratio [OR], 1.92; 95% confidence interval [CI], 1.00-3.67), African American race/ethnicity (OR, 1.91; 95% CI, 1.28-2.87), and residence at a group of geographically proximate public housing complexes (OR, 2.50; 95% CI, 1.25-4.98); older age was inversely related (OR, 0.89; 95% CI, 0.82-0.96 [for each decade increase]).
Immergluck L.C.; *et al.* 2019 [94]<br><br>United States of America (USA), Metro Atlanta	Children n= 5379 with ethnicity categorised as White, Black, Hispanic, and other (included: Asian, Native American, multi-racial, Native Hawaiian, and declined) attending an urban hospital.	Race, age, sex, overcrowded housing, health insurance, household income level.	Children requiring hospital treatment.	Community Associated methicillin resistant *Staphylococcus aureus* CA MRSA)	Quantitative retrospective epidemiology study to identify risk factors and characterise geographic variations of CA-MRSA rates compared to community associated methicillin-susceptible *Staphylococcus aureus* (CA-MSSA) rates between 1 January 2002 and 31 December 2010. Data sourced from two paediatric hospitals in the region. Children seen in the emergency department and or inpatients were included. CDC guidelines were used to define cases as community associated, or hospital acquired infections. American Community Survey data was used to determine household crowding, proportion of Black people by block group, and below poverty. Race/ethnicity, children living in poverty, and	Children with no or public health insurance had higher odds of CA-MRSA compared to those with private insurance ($p < 0.01$). Black children were one and a half times (aOR 1.58 (1.44-1.75, $p < 0.0001$)) as likely as Whites to have CA-MRSA infection, after adjusting for gender, age, and health insurance. Modelling at the neighbourhood level showed significant differences between CA-MRSA and CA-MSSA, with risks for antibiotic resistance being higher in blocks with concentrated Black populations (> = 40.46%, p < 0.001). Blocks in the third and fourth quartiles of crowding had higher odds of poverty levels (3rd quartiles OR 1.32; 95% CI 1.17 - 1.48; p-value < 0.0001; 4th quartiles OR 1.54, 95% CI 1.38 -1.75, p < 0.0001). Multi-level or mixed model analysis showed Black people living in neighbourhoods with > = 40.6% Black people, being female, public or self-pay insurance, and younger age remained highly significant for risk of CA-MRSA infections compared to CA-MSSA, even after adjusting for income, health insurance, gender, crowding, and race/ethnicity.

Author, Year, Title, Country, region and reference	Population sample	Indicators of social determinants of health included as data	Evidence of health seeking behaviour	Infection related data	Study design and aim	Key findings
					median household income were obtained from 2010 Decennial Census.	
Intahphuak, S.; *et al.* 2021[95]<br><br>Thailand Lahu villages from Wiang Pa Pao District, Chiang Rai Province	Lahu men, women, and children over 15 years old n= 240 in rural villages	Age, sex, marital status, multi morbidities, education status, employment status.	Seeking treatment with an infectious disease.	Respiratory tract infection, urinary tract infection (UTI), Gastrointestinal infection - assessment of antibiotic resistance	Quantitative cross-sectional study aimed to estimate the prevalence and factors associated with antibiotic resistance in the stated population. To assist with recruitment the village chiefs were given information on infection signs and symptoms. Participants were asked to collect applicable specimen (stool, sputum, urine). A questionnaire was developed comprising of sociodemographic information, use of antibiotics in last 12 months, knowledge of infectious diseases, knowledge of antibiotics in daily life, attitudes towards antibiotics, antibiotic behaviours, and basic health information. Assistance was provided by 15 village health volunteers who were fluent in Thai and Lahu.	A total of 240 people participated 70.4% were female, 25.4% were aged 30-40 years and 82.9% were married. More than half worked in the agricultural sector (55.4%) and had poor education, which was less than primary school (45.8%). UTIs were the largest group of infections over 12 months (67.9%), with 163 cases. 30/187 specimens had drug resistance The main sources of antibiotics were district hospitals (51.7%), drugstores (27.9%), and private medical clinics (22.5%). Most sources showed good knowledge of infectious diseases (82.9%). Knowledge of and attitudes toward antibiotics and practices were poor (66.2%, 51.2%, and 64.6%, respectively). Those who had poor knowledge of antibiotic use had a 2.56-fold greater chance (95% CI = 1.09-5.32) of having antibiotic resistance than those who had good knowledge of antibiotic use. People who had poor antibiotic use behaviours had a 1.79-fold greater chance (95% CI = 1.06 -4.80) of having antibiotic resistance than those who had good antibiotic use behaviours.

Author, Year, Title, Country, region and reference	Population sample	Indicators of social determinants of health included as data	Evidence of health seeking behaviour	Infection related data	Study design and aim	Key findings
Kurz, M.S.E.; *et al.* 2017 [96]<br><br>Rwanda, Butare	Men and women patients and caregivers attending hospital n=392	Education status, employment status, housing, home environment e.g. water by well, external latrines.	Attending hospital, screening process.	Surveillance and risk factors of intestinal carriage of extended-spectrum beta-lactamase-producing Enterobacteriaceae (ESBL-PE)	Quantitative study to assess the presence and risk factors of intestinal carriage of ESBL-PE among admitted patients and attending caregivers. Analysed the acquisition of ESBL-PE carriage during hospital stay and the associated factors. Microbiology and rectal swabs were undertaken within 48 hours of admission and at discharge.	At admission, 49.7% (195/392) of patients and 37.4% (135/361) of caregivers carried at least one ESBL-PE species. After a median hospital stay of 6 days, proportions increased to 64.6% (173/268, P = 0.01) and 46.5% (106/228, P = 0.06) among patients and caregivers, respectively. Almost one-third of admitted patients (124/392) were not available for follow-up, mostly due to prematurely leaving the hospital. Among ESBL-PE isolated from admitted patients, *Escherichia coli (E. coli)* was the predominant species (51.3%, 100/195), *Klebsiella pneumoniae* (38.5%, 75/195) and *Enterobacter cloacae* (18.5%, 33/195). Patients accompanied by a caregiver colonised with ESBL-PE had 3 times increased odds of being carriers themselves. Univariate analysis showed patients' ESBL-PE carriage associations as being male, referral case, processing antibiotic treatment, few household rooms, chicken husbandry, an unprotected spring as main source of drinking water, preferential consumption of tomatoes and eggs and a distant health centre. Factors reducing the odds involved nearby residence, formal education, electricity or a TV set at home, hand cleaning with soap after defaecation and boiling drinking water.

Author, Year, Title, Country, region and reference	Population sample	Indicators of social determinants of health included as data	Evidence of health seeking behaviour	Infection related data	Study design and aim	Key findings
Lambourg, E.; *et al.* 2022 [97]<br><br>United Kingdom, England	NHS prescribing data for men, women, and children. Approximately 10 million records per month	Age, sex and indices of social deprivation, income level and multi morbidities.	Obtaining antibiotics	Proxy: antibiotic usage linked to indices of deprivation.	Quantitative cross sectional retrospective analysis of General Practices prescribing data to determine: a) the association between socioeconomic inequalities and pharmaceutical consumption, b) determine the interaction between deprivation level, c) temperature affecting prescribing rates. Data used included: a) NHS Business Services Authority prescription database, from 1 January 2011 to 31 December 2018. b) UK Deprivation data IMD (2019 version) Ministry of Housing, Communities and Local Government, c) Disability adjusted life years (DALY) rates were obtained from the global burden of disease (GBD) tool The dependent variable was the average number of items dispensed per day, aggregated per Lower Super Output Area (LSOA), and normalised per thousand inhabitants to account for each LSOA' s exact population.	Results showed antibiotics were prescribed at a higher rate in the most deprived area. Antibiotic prescription rates showed a 9% general decrease in England between 2011 and 2017, with a similar trend for most and least deprived areas. Even after accounting for temperature and DALYs attributable to skin, sexually transmitted, enteric and respiratory infections, antibiotics remained more prescribed in deprived areas by 29%. DALYs failed to explain the higher prescription volume in the most deprived areas suggests that the excess in prescription could be unrelated to a proper health need.
Lanyero, H.; *et al.* 2020 [98]<br><br>Uganda, Gulu	Children under 5 years old and their care giver in rural areas n= 856 households. Care givers mostly not educated and peasant farmers.	Employment- subsistence agriculture. Care givers - distance to health facility within one hour walking distance. Location of household, education of care giver, occupation of care giver.	Seeking treatment from various facilities and factors including distance to health facility.	Respiratory infection	Quantitative cross-sectional study to investigate the prevalence and factors associated with use of antibacterials in the management of symptoms. Data were collected from child caregivers in households with participants who had displayed symptoms of acute respiratory infections (ARIs) within two weeks preceding the data collection date.	Out of 856 children under five years with symptoms of ARIs, 515 (60.2%; CI: 54.5 - 65.6) were treated with antibacterials. The prevalence of antibacterial use was higher in males (51.5%; CI: 47.4- 55.5), children aged 13 - 36 months (46.4%; CI:40.6 -52.3) and those who attended a health facility (57.3%; CI: 49.9 - 64.3). Antibacterial use was higher if the caregiver was a female (96.5%; CI: 94.5 - 97.8), had no education (56.3%; CI: 48.1- 64.2), was a peasant (60.2%; CI:49.0 -70.4), and was aged 25 -34 years (41.2%; CI: 36.8 - 45.7). Antibacterial use was higher in rural areas (80.6%; CI: 61.9 - 91.4). In Gulu, 60.2% of children under five years received antibacterials for symptoms of ARIs.

**Author, Year, Title, Country, region and reference**	**Population sample**	**Indicators of social determinants of health included as data**	**Evidence of health seeking behaviour**	**Infection related data**	**Study design and aim**	**Key findings**
Larramendy, S.; *et al.* 2021 [99]<br><br>France Pays de la Loire, Bretagne	Antibiotic susceptibility of *Escherichia coli (E. coli)* isolated from urine samples of individuals analysed in non-hospital laboratories n= 358291	Age, sex, socioeconomic deprivation, education status, employment status, work environment, income status, size of household, proximity of healthcare services.	People attending nonhospital facilities for urine testing	Prevalence of ESBL-producing *E. coli* in community-acquired urinary tract infection.	An observational, cross-sectional study to determine whether the ESBL-*E. coli* rate in urine samples was associated with the socio-economic, environmental, agricultural, and healthcare characteristics of the local community. Retrospective data was used from the MedQual-Ville network of laboratories who monitor antibiotic resistance in the community.	Specimen data was received from 92 laboratories. There were 358291 *E. coli* isolates and the mean ESBL-*E. coli*. prevalence rate was 3.30%. The multivariate analysis identified seven features that were significantly associated with the ESBL-*E. coli* rate: two socio-demographic characteristics: percentage of people >65 years old (P < 0.001) and the deprivation index (P = 0.002). Two healthcare linked association - number of hospital beds (P = 0.017) and patients taking third generation cephalosporins (P < 0.016). Three agri-environmental characteristics - density of pigs (P = 0.017) and poultry (P = 0.024) and percentage of agricultural land.
Larson, E., *et al.* 2003 [100]<br><br>United States of America (USA), Manhattan	Hispanic men, women and children group 1 (n=398 households with 1,645 individual participants). Group 2 households (n=238 with 1,177 participants). Single urban neighbourhood of working poor and middle-class immigrants.	Multi morbidities, age, sex.	Use of antibiotics	Respiratory tract infection, gastrointestinal, fever and skin infections.	Quantitative cross-sectional study to determine community prevalence and antibiotic use in the stated population. This study was part of a larger clinical trial, convenience sampling including use of posters, brochures, word of mouth were used. Eligibility for the study included households having at least 3 participants including a preschool child and the ability to speak in Spanish or English. The questionnaire was psychometrically assessed, and pilot tested. Trained interviewers visited households to assess symptoms and treatment. Analyses were conducted at the level of the individual household member. Statistical analysis to compare demographic and illness variables between the two survey periods.	Infectious disease symptoms reported in 911 (33.2%) of 2,743 household members in the previous 30 days: medical attention was sought by 441 (48.4%) of 911 persons, and 354 (38.9%) of 911 took antibiotics for symptoms. Reported symptoms were respiratory (68.9%), gastrointestinal (15.3%), fever (12.8%), and skin infection (2.8%). Medical attention was sought significantly more often among those with chronic illness, those born in the United States, and those with fever, runny nose, or skin infections (all p<0.05). Antibiotics were taken significantly more often among those with poor health, those who spent more time at home, and those with fever and respiratory symptoms. Recommendations included interventions involving both clinicians and the public, and for the Hispanic population interventions need to be culturally specific and in Spanish.

Author, Year, Title, Country, region and reference	Population sample	Indicators of social determinants of health included as data	Evidence of health seeking behaviour	Infection related data	Study design and aim	Key findings
McMullen, K. M.; *et al.* 2009 [101]<br><br>United States of America (USA)<br>St Louis, Missouri	Caucasian, African American and other men and women attending an urban hospital. n= 10,530 cultures	Age, sex, ethnicity, income level.	Attending hospital	Community-associated methicillin resistant *Staphylococcus aureus* (CA MRSA) and skin infections.	Quantitative cross sectional prevalence study to review hospital (a 1250-bed academic medical centre) data over a ten-year period from 1996 to 2005. Positive methicillin resistant *Staphylococcus aureus* (MRSA) cultures were extracted from the microbiology database including all outpatients, inpatients, and emergency department attendances. The analysis involved the patients first positive culture and antibiotic sensitivities. The patients home ZIP code was linked to the median household income from 2000 US census data. Low socioeconomic status (SES) was determined if the median income for the home ZIP code was $25,000 or less.	Positive MRSA cultures increased from 1.0 positive cultures per 1000 ED visits to 3.3 positive cultures per 1000 ED visits over the study period. CA-MRSA phenotype significantly increased from 8.9% (n= 43) in 1996 to 39.6% (n= 672) in 2005. Overall, MRSA and CA-MRSA showed significant trends toward decreasing median age; the median age for all emergency department (ED) patients remained at approximately 49 throughout the study period. There was a significant trend toward increasing proportion of non-Caucasian race in both the overall and CA-MRSA results. Among the CA-MRSA patients, the proportion of non-Caucasian patients increased from 30.2% in 1996 to 60.4% in 2005. A trend was also seen in the SES of the CA-MRSA patients, with the proportion of patients categorised as low SES increasing from 25.6% to 35.6% over the study period. Patients of non-Caucasian race and lower SES may be at increasing risk for MRSA, especially CA-MRSA.
Moremi, N.; *et al.* 2017 [102]<br><br>Tanzania, Mwanza city	Urban street children n= 107	Age, sex, housing, water, sanitation and hygiene (WASH), education status, source of income and food, proximity of healthcare services, use of traditional medicine.	Street children attending a health camp.	Surveillance for faecal carriage of CTX-M extended-spectrum beta-lactamase-producing Enterobacteriaceae	Quantitative cross-sectional study to examine the prevalence of ESBL-producing Enterobacteriaceae (EPE). A structured survey was used to assist with interviews. Participants street lifestyle was categorised as type 1 "on street children" (those who spent the day on streets and at night slept either at home or in street institutions for children) and type 2 "of street children" (those who spent the day and sleep on streets). General examinations included anthropometric measurements. Stool samples were assessed for EPE.	There were 63/107 (58.9%) Type 2 children. 10 (9.3%) completed primary education, 60 (56.1%) stopped attending and 37 (34.6%) did not attend primary education. 30 (28%) used antibiotics in the past 4 weeks prior to the study. 34 (31.8%, 95% CI; 22.7 - 40.3) were colonised with at least one ESBL-producing *Escherichia coli* (*E. coli*) or *K. pneumoniae* isolate. Of the 36 ESBL-producing isolates detected, 83.3% (30/36) and 16.7% (6/36) were *E. coli and K. pneumoniae*, respectively. All 36 ESBL isolates were resistant to a range of antibiotics. Higher ESBL carriage was observed among children who used local herbs for paramedical purposes compared with those who did not use herbs (50% vs. 22.2%, P = 0.003. Univariate analysis indicated that ESBL carriage was significantly higher in type 2 street children (OR: 3.6). There was no association between either source of food or education level and EPE carriage. There was no significant association of ESBL carriage in children

Author, Year, Title, Country, region and reference	Population sample	Indicators of social determinants of health included as data	Evidence of health seeking behaviour	Infection related data	Study design and aim	Key findings
						who reported having used antibiotics and those who did not report use (33.3% vs. 33.7%).
Negash, A.A.; *et al*. 2019 [103]<br><br>Ethiopia<br>Addis Ababa	Children who were attending urban paediatric emergency departments aged 0 to 15 years old n= 549	Socioeconomic deprivation, education status, home environment e.g. water by well, external latrines.	Attending hospital emergency departments	Respiratory tract infection and community acquired pneumonia (CAP).	Quantitative study to examine prevalence, aetiology, and risk factors of bacteraemic CAP and the role of *S. pneumoniae* in causing bacteraemic pneumococcal CAP after the introduction of pneumococcal conjugate vaccine (PCV10). Caregivers consulted, structured questionnaire used for demographic details. Blood specimens collected for analysis and antibiotic susceptibility testing.	Enrolled participants showed 321 (58.5%) were males. The prevalence of bacteraemic pneumonia was 5.6% (31) and most (61.3%) were females (P = .022). The median age of the children was 9 months. 93.7% were infants. The overall in-hospital case fatality rate was 2.37% (13/549). Nutritional status was the only variable associated with mortality. Children severely malnourished (WAZ < .001). Predominant pathogens were *Staphylococcus aureus* (26.5%), *Enterococcus faecium* (11.8%), *Escherichia coli* (11.8%), and *Klebsiella pneumoniae* (11.8%). In univariate analysis, parental smoking, and non-vaccination with PCV10 were associated with bacteraemic CAP. In multivariable analysis, female sex and malnutrition were associated with increased bacteraemic CAP.
Piper Jenks N.; *et al.* 2016 [104]<br><br>United States of America (USA),<br>New York	Hispanic and non-Hispanic men, women and children. Aged between 7- and 70 years. n= 129 attending an urban community health centre.	Age, sex, multi morbidities, income level, affordability of healthcare and education status	Attending community health centres providing services for immigrants.	Wound and nasal swabs prevalence of methicillin resistant *Staphylococcus aureus* (MRSA) and methicillin-susceptible *Staphylococcus aureus* (MSSA)	Quantitative study conducted as part of a research collaborative. The aim was to assess the prevalence and differences of community associated (CA)-MRSA/MSSA in patients with skin and soft tissue infections (SSTIs) in Community Health centres and identify risk factors, clinical outcomes, and recurrence. Characterisation was performed in all *S. aureus* isolates from wounds and nasal swabs collected from patients. Statistical analysis was undertaken to review the differences in specimens from immigrants and native-born participants.	Participants included 40 patients who were immigrants from fifteen countries. Infection sites included axilla (18.8%), buttocks (12.8%), head and neck (10.3%) and lower leg (10.3%). Foreign-born patients were more likely to have lesions located on the thigh than native-born patients (p Z 0.03), and more likely to have a wound characterised by draining pus (p Z 0.06). MRSA wound infections were found to be similar in both population groups. MSSA wound cultures were significantly different 28.2% of foreign-born participants had MSSA in their wounds compared to 10.8% of native-born participants (p Z 0.03). MSSA nasal carriage was similar in both groups (21.9% in native-born, 28.2% in foreign-born, p Z 0.49). The study outlined differences may be due to variations in healthcare received in the country of origin and the USA. Antibiotic use was also found to be less in immigrants.

Author, Year, Title, Country, region and reference	Population sample	Indicators of social determinants of health included as data	Evidence of health seeking behaviour	Infection related data	Study design and aim	Key findings
Quagliarello, A.B.; *et al.* 2003 [105]<br><br>Vietnam<br>Ho Chi Minh City, Dong Nai province	Children attending schools n= 529, (n=327 from urban and n=202 from rural areas)	Age, proximity and affordability of healthcare, use of traditional medicine.	Access to health facilities and use of antibiotics.	Carriage of penicillin-resistant *Streptococcus pneumoniae*.	Quantitative study to a) examine whether levels of antimicrobial resistance in *S. pneumoniae* were still evident three years after the original study b) determine whether a relationship existed between resistance patterns and patterns of antimicrobial usage. Methods included: nasal swabs taken for antimicrobial susceptibility. Questionnaires were given to caregivers to assess antibiotic usage and healthcare-seeking behaviour. An intervention study using roleplay - staff members attended local pharmacies to assess whether they could obtain antibiotics.	Nasal carriage study: penicillin-resistant pneumococcus was 15 times more likely in urban participants compared to rural school children. Penicillin, erythromycin and co-trimoxazole resistant pneumococci were still significantly more common among urban children compared to rural children. Questionnaire: antibiotic consumption - 80% of urban children had taken an antibiotic compared to 65% of rural children. Urban parents were more likely to see private doctors than rural parents and less likely to seek treatment at pharmacies. Despite length of transportation, pharmacies were the first choice for rural parents mainly due to 'cost'. Pharmacy study: both urban and rural pharmacies routinely prescribed antimicrobials and frequently dispensed inappropriate dosages. Antimicrobials were more likely to be given for respiratory cases. There was an increase in the carriage of non-susceptible *S. pneumoniae* in rural children over the three-year study period.
Ravensbergen, S.J.; *et al.* 2019 [106]<br><br>Netherlands, Northeast region	Asylum seekers - men and women requiring medical care at an urban hospital. Screening process n= 2091	Age, sex	Screening on arrival into new country.	Prevalence of multi drug resistant microorganisms.	Quantitative cross-sectional study to assess the duration of multi drug resistant organisms (MDRO) carriage. Samples were collected between 1 January 2014 and 31 December 2016 from participants who had presented for medical care. Samples included screening for MDRO carriage before admission (throat, rectum, and nose) and clinical samples. Organisms included methicillin-resistant *Staphylococcus aureus* (MRSA) and multi-drug resistant Enterobacteriaceae (MDRE). Postal codes were used to identify patients as asylum seekers for the study. Demographic data including Country of origin and arrival date was documented.	MRSA: 185 (9.3%) were positive. MDRE: 331 (18.5%) tested positive. MRSA and MDRE were more frequently detected in asylum seekers originating from Iraq (19.1 and 43.2%, respectively) and Syria (15.8 and 39.9%, respectively). *Escherichia coli (E. coli)* was the most frequently detected MDRE species (n = 301). The most frequent resistance pattern detected was ESBL-production followed by a combination of ESBL-production and Fluoroquinolone/Aminoglycoside Resistance. For MRSA, the median time after arrival - over 53 weeks was 1022 days (IQR 494 - 1892) and for MDRE was 1063 days (IQR 510 - 1950). The study demonstrated that MDRO in the studied population remained high even after a prolonged immigration period.

Author, Year, Title, Country, region and reference	Population sample	Indicators of social determinants of health included as data	Evidence of health seeking behaviour	Infection related data	Study design and aim	Key findings
Sloth L. B.; *et al.* 2019 [107]<br><br>Denmark, National study.	Data set including migrant and non-migrant men and women n= 14561 first-time urine samples.	Age, sex, income level.	Treatment for infection.	Antibiotic resistance patterns of *Escherichia coli* (*E. coli*) in people with urinary tract infection.	Quantitative cross- sectional study to investigate the distribution of urine isolates and antibiotic resistance patterns in *E. coli*. Data were analysed by examining urine cultures from both migrant and non-migrant populations.	4,561 first-time urine samples were included with *E. coli* being the most prevalent (79.9%). *E. coli* isolates, 4686 / 11737 -non-migrants 1032 / 2824 migrants (1873 from family-reunited migrants and 951 refugees). Multi-resistant *E. coli* isolates were rare however, there was a significant difference between migrants 4.1% and non-migrants 1.4% $P < 0.001$. Antibiotic resistance was more prevalent in family-reunited migrants compared with refugees. Higher odds of resistant *E. coli* isolates were seen among migrants from all five regions except Latin America and the Caribbean.
Stabler, S.; *et al.* 2022 [108]<br><br>France<br>Paris	Migrants who attended the Infectious Diseases department and were inpatient and or outpatients of Saint-Antoine university teaching hospital. Men and women n= 101. Region of origin: Sub-Saharan Africa, South America, Asia, Eastern Europe, North Africa, the Middle East.	Age, sex, marital status, education status, previous employment status, affordability of healthcare, housing.	Sought care from a tertiary hospital.	Prevalence of methicillin resistant *Staphylococcus aureus* (MRSA), extended spectrum beta-lactamase-producing Enterobacteriaceae.	Quantitative monocentric, cross-sectional study to estimate the rate of carriage of multidrug resistance (MDR) and extensive drug-resistant (XDR) bacteria and identify factors associated with (antimicrobial) AMR carriage. Patients who were migrants defined as a) foreign-born b) having arrived in France within 12 months from Eastern Europe, Africa, the Middle East, Asia, or South-America were eligible. Retrospective analysis of the defined patients' medical records was undertaken for the period November 2017 to the end of October 2018. Clinical and biological variables were obtained from the post-arrival survey which included demographics, medical history, history of migration and microbiological data, and forms the screening policy for migrant patients.	1023 records were screened, following exclusions the final 101 comprised of 71 were male (70.3%) with a median age of 30 years. 79 (78.2%) migrants from Sub-Saharan Africa and 30 (30.9%) were asylum seekers. Migrant routes included direct flight (n = 31, 32%), through Libya and Italy (n = 32, 33%), and through Morocco and Spain (n = 24, 24.7%). The median length of migration was 21.7 weeks and 30.1% had lived in at least one refugee camp. In France 65% were either homeless or living in poor conditions. Overall carriage rate of AMR bacteria was 20.7% [95% CI: 12.4; 28.9%]. 5/92 (5.4%) MRSA strains and 15 (16.3%) extended-spectrum beta-lactamase producing Enterobacteriaceae (ESBL-E), including 11 *Escherichia coli*, 3 *Klebsiella pneumoniae* and 1 *Enterobacter cloacae*. In univariate analysis, patients with ESBL-E carriage were significantly older (36 years-old vs. 28 years-old, $p < 0.001$) and originated from Eastern Europe (20% vs. 3.9%, $p = 0.05$) and North Africa (13.3% vs. 1.3%, $p = 0.05$). Patients with ESBL-E carriage more often travelled by direct flight ($p=0.033$). In multivariate analysis only older age remained independently statistically associated with ESBL -E carriage ($p=0.008$).

Author, Year, Title, Country, region and reference	Population sample	Indicators of social determinants of health included as data	Evidence of health seeking behaviour	Infection related data	Study design and aim	Key findings
Sutter, D.E.; *et al.* 2011[109]<br><br>Afghanistan, Bagram Air Base.	n=266 Afghan and American patients including men, women and children attending a field hospital.	Ethnicity, age, sex	Attending deployed United States of America (USA) Military Hospital for treatment post trauma.	Wounds post trauma, burns, urinary tract infection. A range of gram negative and gram-positive isolates acquired in both the community and hospital.	Quantitative cross-sectional study to examine the epidemiology of multi drug resistance (MDR) bacteria including whether organisms originated in the community or in the hospital environment. Retrospective analysis of clinical and microbiologic data obtained from September 2007 to August 2008. Clinical data were extracted from admission notes, history and physical examinations, operation notes, and discharge summaries. Specimen sources included blood, cerebrospinal fluid (CSF), sputum, urine, wound, and stool. Environmental sampling of select hospital surfaces and equipment undertaken in August 2008. Bacteria were identified and antimicrobial susceptibility testing was determined using the Microscan Autoscan 4.	The 266 patients had 411 bacterial isolates identified of which 211 were MDR bacteria (51%). USA personnel with positive bacterial cultures were mainly outpatients (79%). However, Afghans with positive culture results were predominantly inpatients (83%). Gram negative bacteria were frequently seen in Afghan patients 241 (76%) of 319 and 70% were MDR. These included *Escherichia coli* (53% were MDR), *Acinetobacter* (90% were MDR), and *Klebsiella* (63% were MDR). Almost one-half of potential extended-spectrum b-lactamase (ESBL) producers were community acquired. 113 of 166 Afghan patients (68%) had 1 or more types of MDR bacteria recovered, compared to only 19% of USA personnel. Positive Afghan outpatient cultures were mainly from wound samples (73%), in contrast to Afghan inpatients which included wound (40%) and sputum or endotracheal aspirate (36%).

Author, Year, Title, Country, region and reference	Population sample	Indicators of social determinants of health included as data	Evidence of health seeking behaviour	Infection related data	Study design and aim	Key findings
Tola, M.A.; *et al.* 2021 [110]<br><br>Ethiopia, Addis Ababa	Children under 5 years old attending the Addis Raey urban public health centre as outpatients<br>n= 269 children	Age of child and care giver, sex of child. Education status of mother, housing and home environment e.g. water by well, external latrines. Parents income level and size of family. Availability and proximity of health services.	Attending the outpatient department of a hospital.	Prevalence of extended-spectrum beta lactamase-producing *Escherichia coli* (*E. coli*) and *Klebsiella pneumoniae* faecal carriage.	Quantitative cross-sectional study from April to May 2017 to determine the prevalence of ESBL producing *E. coli* and *K. pneumoniae* faecal carriage. Consecutive sampling technique was used to collect the stool/rectal specimens. Information on socio-demographic and associated risk factors was collected using structured pretested questionnaires. Medical records were reviewed to assess antibiotic usage for the previous twelve months. Fresh faecal / rectal swabs were collected and analysed using VITEK 2 - automated microbiology bacterial identification and antimicrobial susceptibility system.	216 (80.3%) had exposure to previous antibiotic usage. A total of 264 *E. coli* and *Klebsiella* species were isolated from 269 faecal/rectal swab samples. *E. coli* 224 (84.8%) was the most prevalent followed by *K. pneumoniae* 39 (14.8%) and *K. oxytoca* 1 (0.4%). The overall prevalence of ESBL producing *E. coli* and *K. pneumonia* faecal carriage was 17.1% (46/269; 95% CI: 12.9% -22.7%). ESBL faecal carriage proportion was higher among children in the age groups of 29 days to 23 months 19.4% (30/155). ESBL faecal carriage was slightly higher among children who had moderate acute malnutrition 21.4% (3/14). ESBL carriage was low in children with a history of prior intake of antibiotics 16.7% (36/216). Overall, 65.4% (172/263) of ESBL producing and non-ESBL producing *E. coli* and *K. pneumoniae* isolates were multidrug-resistant (MDR, resistance to at least 3 antibiotics from different classes). Only children's mothers who had lower educational level (primary school) (OR: 2.472, 95% CI: 1.323 - 4.618, P = 0.0062) and children who used tap water for drinking (OR: 1.714, 95% CI: 1.001 - 3.659, P = 0.048) were significantly associated with higher ESBL faecal carriage.
Tornberg-Belanger, *et al.* 2022 [111]<br><br>Kenya, Western Kenya	Children aged 1 to 59 months n=406 who had been admitted to a rural hospital.	Age, sex, size of household, home environment e.g. water by well, external latrines, keeping livestock. Deprivation, including stunting and or wasting.	Screening on hospital discharge and trial of azithromycin to assess outcome improvement.	Prevalence and risk factors of *Escherichia coli* (*E. coli)* including Extended Spectrum Beta Lactamase (ESBL).	Quantitative cross-sectional study to determine the prevalence and risk factors of resistance to frequently used antibiotics in commensal *E. coli* isolated from participants discharged from hospital. Assessed whether a 5-day course of azithromycin reduces rehospitalisation and/or death in the subsequent 6-month period. Standardised questionnaire used for interviews with Caregivers to obtain sociodemographic information and medical history. Clinical information extracted from medical records. Physical examination undertaken by	Most frequent diagnoses were pneumonia, diarrhoea, anaemia and malaria. 406/448 participant faecal specimens had *E. coli* (90.6%). The median age was 19 months and 59.4% were male. Almost all (92.6%) children had *E. coli* isolated that lacked phenotypic susceptibility to ampicillin. Risk factors - administration of antibiotics while in hospital was positively associated with the presence of ESBL producing *E. coli* (adjusted prevalence ratio [aPR] = 2.23; 95% CI: 1.29 -3.83). The practice of open defaecation was associated with ESBL-producing E. coli (aPR = 2.02; 95% CI: 1.39-2.94) and sharing of sanitation facilities with other households (aPR = 1.49; 95% CI: 1.17 -1.89). Living in crowded housing and availability of improved water source were not associated with ESBL producing *E. coli*.

Author, Year, Title, Country, region and reference	Population sample	Indicators of social determinants of health included as data	Evidence of health seeking behaviour	Infection related data	Study design and aim	Key findings
					a study clinician and whole stool, or rectal swabs were collected.	
Williamson D.A.; *et al.* 2013 [112]<br><br>New Zealand, Auckland	Data set including men, women, and children n= 1507 who had attended an urban hospital. Ethnicities included European, Asian, Pacific Island peoples, Māori and other.	Ethnicity, age, and sex.	Hospital inpatients.	Community-associated and healthcare-associated *Escherichia coli* (*E. coli)* bloodstream infection.	Quantitative cross-sectional study to a) assess the incidence, antimicrobial resistance trends and outcomes of *Escherichia coli* bloodstream infection (EC-BSI) b) describe demographic variations in the incidence of community-associated (CA) vs. healthcare- associated (HCA) EC-BSI. Cases of EC-BSI were identified from the laboratory database for patients who had attended hospital between January 2005 and December 2011. Defined criteria were developed for both CA and HA infections. Clinical outcome measures included: length of hospital stay (days), requirement for intensive care unit admission, and all-cause in-patient hospital mortality. *E. coli* isolates were identified using the RapID One system.	There were 1507 patients with ECBSI during the study. The overall average annual incidence of EC-BSI was 52 per 100,000 population with the highest incidence in under 1 and over 65 age groups. The overall rate increased from 42 to 60 per 100,000 population over the study period. When stratified by ethnicity, the incidence was highest in Māori and Pacific Peoples 83 and 62 per 100,000 population, respectively. 806 (54.9%) patients were of European ethnicity, 288 (19.6%) were Pacific Peoples, 220 (15.0%) were Asian, 133 (9.1%) were Māori and 20 (1.4%) were of other ethnicities. The 1507 episodes of EC-BSI, 510/1507 (34%) were classified as CA EC-BSI, 608/1507 (40%) were HCA-CO EC-BSI, and 389/1507 (26%) were HCA-HO EC-BSI. The incidences of HCA-CO and HCA-HO EC-BSI did not increase significantly however, the incidence of CA EC-BSI increased significantly from 13 per 100,000 to 22 per 100,000 population ($P < 0.001$). Rates of resistance to any of the tested antimicrobials did not increase significantly over the study period. The incidence of EC-BSI showed significant demographic variation particularly in ethnic-specific incidence rates.
Zoorob R.; *et al.* 2016 [113]<br><br>United States of America, (USA)<br>Harris County, Texas	Hispanic, Black, White, Asian, and other men and women n= 400 who attended urban primary care clinics.	Race /ethnicity, age, sex, education status, employment status, income level.	Proxy: non prescribed use of antibiotics	Upper respiratory tract infection, urinary tract infection and use for tooth pain, stomach pain, and general infection.	Cross sectional study to a) assess the prevalence of nonprescription antibiotic use in the previous twelve months including intention to use antibiotics without a prescription and storage of antibiotics b) examine patient characteristics associated with nonprescription use in a random sample of adults.	The prevalence rate of nonprescription antibiotic use in the 12-month period was 5%. Intended use had a higher prevalence (25.4%) than actual nonprescription use, indicating that the population at risk is much larger than the population of those who have used nonprescription antibiotics in the previous 12 months. The prevalence rate of storage of antibiotics was 14.2%, indicating that many participants have antibiotics available at home. Antibiotics were frequently used for respiratory symptoms. In multivariate analyses, public clinic patients, those with less education, and younger patients were more likely to be intended users.

Evidence for secondary question A - What evidence is there from the global literature on AMR related interventions which specifically target marginalized / vulnerable populations?

Table 6: Included studies examining general interventions in relation to AMR

**Author, Year, Country, region and reference**	**Population and inclusion health groups**	**Evidence of the social determinants of health**	**Evidence of health seeking behaviour**	**Infection**	**Aims and Objectives**	**Key findings**
Hood G.; *et al.* 2019 [114]<br><br>United Kingdom England	Number not stated. Population not stated for each campaign.	Sex and ethnicity but not stated by ethnic group. Protected characteristics included in the Health Equity Assessment (HEAT) assessment.	Knowledge and use of antibiotics	Proxy: antibiotic knowledge and awareness	Assess whether antimicrobial (AMR) initiatives are inclusive and reaching diverse populations. Assessment of multiple public awareness campaigns including Antibiotic guardian, e bug, TARGET, Keep antibiotics working by using World Health Organisation (WHO) HEAT map. Qualitative, Case report<br>1. HEAT map developed by Public Health England to assess the campaigns inclusivity<br>2. Completed by the leads for each intervention e.g. TARGET leaflets.<br>3. Focus to assess protected characteristics and inclusivity.<br>4. 8-week timescale for completion	The four campaigns were found to be inclusive as outlined in the Equality Act 2010. However, some protected characteristics were not applicable. Recommendations included development of further resources using diverse user groups. HEAT tool identified as an effective method of assessing health inequalities.
Saito, N.; *et al.* 2018 [115]<br><br>Philippines Manila	n= 410 men and women attending an urban secondary care Hospital - San Lazaro Hospital (SLH) for a medical consultation.	Sex, age, monthly income, living area.	Assessment of self-medication	Proxy: use of antibiotics.	Investigating prior antibiotic use by using a urine bioassay to detect antibiotic use. Quantitative, prospective observational study. Sociodemographic and clinical information collected using a standard questionnaire. Additional information collected was the clinical diagnosis, examination findings, antibiotic use during the illness. Urine samples were collected before antibiotics were commenced in the hospital. Twenty urine samples of healthy volunteers with no history of antibiotic use were used as a negative control.	164 (40%) of people, predominantly from low-income urban areas had a positive specimen showing use of antibiotics prior to consultation. There was also a significant association between patients' monthly income and a positive urine bioassay result. Patients obtained antibiotics from community pharmacies, private clinics, and public health centres. Medication was not obtained from friends, relatives, or charities. Most antibiotics were beta-lactams; amoxicillin, cloxacillin, amoxicillin/clavulanate, and oral cephalosporins. Fluoroquinolone usage was (8%). Most patients took antibiotics for less than 7 days (91%).

Evidence for secondary question B – What evidence is there on existing AMR related interventions including public participation and co production targeting low socio-economic populations?

Table 7 Included studies that included evidence of participatory action with populations considered at risk of AMR

Author, Year, Country, region and reference	Target Population	Social determinants of health included as indicators	Evidence of health seeking behaviour	Infection related data	Study design and aims	Key findings
Cai H. T. N., *et al.* 2022 [116]<br><br>Vietnam<br>Vu Ban and Giao Thuy Districts of Nam Dinh Province	n=26 adult participants in four villages	Age, sex, ethnicity, socioeconomic deprivation.	Participatory action approach to engage communities. Assessment of behaviours associated with antimicrobial resistance (AMR) and antibiotic use.	Proxy antibiotic behaviour.	Research study was part of a large One health programme. Participatory learning action approach used as a pilot study to explore ways to reduce antibiotic consumption and promote preventive behaviours in healthcare, community, and farm settings. Purposive and self-selection of participants with local partners. Seven meetings and photovoice recordings with photographs of antibiotic use in the community including livestock. Team undertook detailed reflection and adjusted the methodology as required.	Communities did not perceive AMR as a critical situation therefore difficult to engage with communities in limited time. Challenges reported with context, time required to develop trust and relationships with participants and the dynamics of community stakeholders. Five themes identified highlighting community engagement and AMR. Low awareness and low perceived importance about AMR. Detailed theme analysis about the development of the implementation but not the results of Photovoice. Cross discussion about AMR e.g., women and farmers but some frustration from participants about not meeting expectations i.e. no specific lectures about AMR.
Haenssgen M.J., *et al.* 2018 [117]<br><br>Laos<br>Salavan	n=2480 adults from 459 peri urban households.	Sex, age, completed years of formal education, wealth index, ethnic groups, village size, household size, dependency ratio, households owning mobile phones, health literacy.	Detailed treatment seeking indicators.	Proxy: antibiotic awareness and assessment of related behaviours.	Quantitative and quasi experimental design. Household interviews and educational intervention design assessing the impact of an increasing awareness behavioural tool for antimicrobial (AMR).<br>Multiple methods including educational activity - six session activity over half a day, questionnaires, and analysis of two rounds of census data. Knowledge, attitude, and behaviour changes measured by interviews being conducted over a 3-month period before and after the interventional study occurred. 3 study groups including direct, indirect exposure and unexposed. Village head and public health officer present.	Two directional educational activity influenced awareness and understanding of drug resistance by the villagers and the activities informed the research team about participant medicine use. Awareness events reported as useful, however, evidence of behavioural changes very limited. Ambiguous links between awareness, attitudes, and behaviour. Group activities were not scalable for the whole village, dissemination of messages appeared to be to more privileged groups. Large media campaigns may show inequitable uptake and differences across socioeconomic levels. Indirect exposure less successful in reaching the more remote areas of the villages. Proposed solution for areas with high levels of antibiotic usage, poverty and poor access to public healthcare is access to healthcare and medicine rather than awareness raising.

Author, Year, Country, region and reference	Target Population	Social determinants of health included as indicators	Evidence of health seeking behaviour	Infection related data	Study design and aims	Key findings
Haenssgen M.J., *et al.* 2021 [118]<br><br>Laos Southern Lao PDR and Thailand Chiang Rai province	n=1158 adults, youth and children in rural marginalised villages	Sex, age, socio cultural factors, education, family relationships.	Treatment seeking behaviour and comparisons between traditional healing and modern medicine. Comparison between obtaining antibiotics for adults and children.	Case study public engagement interventions to examine local perspectives and people's behaviour about AMR and antibiotic use.	Mixed methods, case study design incorporating medical humanities. Studying how the inclusion of medical humanities methods in public engagement activities can examine the sociocultural context of antimicrobial (AMR) and its related topics of medicine use and health systems. Three interdisciplinary public engagement case studies for marginalised people. Workshops in three villages included co production. Electronic behaviour survey data and questionnaires.	The use of medical humanities with public participation worked and the use of oral narratives and photographic narratives was beneficial in challenging the research teams and provided new areas for consideration in the AMR debate. Recommendations to use the methods to support contextual requirements. However, with preconditions to use studies as learning exercises, and embed decolonisation of global health.
Jones N., *et al.* 2022 [119]<br><br>Nepal, Kathmandu, Chandragiri Municipality and Bhaktapur Lockanthal	n=20 Nepalese men (9) and women (11) in a peri urban community.	Gender, caste, ethnicity.	Healthcare and antibiotic access.	Proxy: antibiotic use and misuse.	Analysis of gendered themes from a qualitative community engagement project exploring antimicrobial (AMR) drivers, using the data set generated through community-based practices following a participatory video project operated in Nepal between 2017 and 2019. The Community Arts Against Antibiotic Resistance Nepal (CARAN) project used for gender data analysis.<br>Different castes, backgrounds and professions included in the five workshops, focus groups and six films.	Gender impacts AMR driving behaviours. Most women only have access to local and free health posts and therefore follow the traditional healer route. Women's inability to access paid medical help without express permission from their husband limits access to medications that may not be freely available at health posts. Men have a wider selection of options, pharmacies, hospitals, and doctors. Men seek the fastest form of recovery for themselves and their children. Female participants discuss barriers to healthcare with the associated power dynamics that reduce women's ability to make health decisions.
Leeman-Castillo B.A., *et al.* 2007 [120]<br><br>United States America (USA) Colorado	n=296 adults at an urban walk-in centre.	Medically underserved community, age, sex, ethnicity, literacy, health literacy, household income level.	Patients seeking urgent care for a respiratory infection.	Upper respiratory tract infection.	Quantitative; intervention study designed to reduce antibiotic use. Two computers placed in the clinic for participant use. A survey was used to collate information about socio demographics, behaviours and attitudes. The survey was undertaken in English or Spanish.	Respondents, who were predominantly young (82% were 18 to 44 years old), female (59%), Hispanic (54%), and poor (50% with annual household income less than $10k).<br>Interactive module with underserved communities was deemed successful after measuring survey responses to three themes complexity; compatibility and relative advantage. Module appropriate for low literacy and people with no information technology (IT) knowledge as audio and visual instructions were included as pre survey instructions / trial site for participants to increase their general IT skills.

Author, Year, Country, region and reference	Target Population	Social determinants of health included as indicators	Evidence of health seeking behaviour	Infection related data	Study design and aims	Key findings
Mainous A.G., *et al.* 2009 [121]<br><br>United States America (USA) Charleston, South Carolina	n= 250 Latino adults in a peri urban community.	Ethnicity, country of birth, age, sex, education, health insurance.	Obtaining antibiotics without a prescription. Exposure to antibiotic messaging via print and or radio.	Proxy antibiotic behaviour.	Quasi experimental pre- and post-intervention study, evaluating the implementation of a culturally sensitive educational intervention to decrease self-medication with antibiotics. Dissemination included community sites, newspaper, and radio. Focus on antibiotic use, resistance, and risks of self-medication. Survey post intervention evaluated the impact of the intervention through logistic regression analysis controlling for potential confounders.	Despite 69.0% of adults surveyed receiving communication about the appropriate use of antibiotics, the exposure did not lead to differences in attitudes about non-prescription antibiotics and or purchasing antibiotics without prescription. Health education intervention insufficient to change participants previous belief that antibiotics without prescription and self-medication are acceptable behavioural practices.
Mitchell J., *et al*. 2022 [122]<br><br>Bangladesh, Ghana, India, Nepal, Vietnam	Challenge cluster project covering six previous community engagement (CE) research studies in LMIC	Education - CE interventions in LMIC	CE projects including health seeking behavioural aspects.	Proxy antimicrobial resistance (AMR) CE projects.	Synthesise knowledge and experience regarding the current, and future uses of CE approaches to address AMR as a One Health issue. Challenge cluster involving six existing AMR research projects. All authors were co-investigators within the cluster who examined current, and future uses of CE approaches for AMR with a One Health approach.	CE can assist with behaviour change as the local communities engage in developing and ensuring the language and content are country and area specific. Solutions are developed locally and are therefore meaningful for the populations. However, these approaches make it difficult to scale and evaluate. The study highlights a requirement for a One Health approach.
Munoz G., *et al*. 2011 [123]<br><br>Ecuador San JosÃ de La Posta and Correuco	n= 65 rural households with children under 5 years old.	Sex, housing, water source, waste disposal, hand washing. Mothers practice re breast feeding and immunisations. Nutritional status. Knowledge assessment both traditional and western.	Treatment practices for stated infections.	Respiratory tract infection and diarrhoea.	Quantitative descriptive study as a baseline assessment to facilitate the development of a longitudinal intervention study. Characterise a) environmental risk factors, knowledge, attitudes, and practices of caregivers, antibiotic use, and child health b) to seek community input in applying an ecosystem approach. Included a small pilot intervention to obtain community views about the elements to include in a longitudinal multicomponent eco-health intervention trial.	Water and sanitation issues were major risk factors. Malnutrition was present in both communities. The study showed the use of both traditional and western medicine including antibiotics in approximately 60% of homes. There were serious knowledge gaps. However, knowledge, attitudes, and practice studies do not demonstrate the breadth of the problem to be addressed. Recommendations included addressing the SDoH e.g. water, sanitation and hygiene (WASH) and undertaking further research in the ecosystem field.

Author, Year, Country, region and reference	Target Population	Social determinants of health included as indicators	Evidence of health seeking behaviour	Infection related data	Study design and aims	Key findings
Stockwell M.S., *et al.* 2010 [124]<br><br>United States America (USA) Washington Heights, New York City	n =15 Latino early head start parents (EHS) at an urban community venue.	Socioeconomic deprivation, education status, health literacy.	Testing a health intervention.	Upper respiratory tract infection.	Non-randomised experimental study aiming to pilot evaluation of the impact of a community-based, culturally competent health literacy interventions focused on knowledge, attitudes, and care practices (KAP). Changes in parental knowledge and attitudes including health literacy level were assessed with a KAP tool before and after the educational modules were presented.	Parents found the sessions beneficial and requested longer sessions without their children. One parent reported seeking antibiotics without prescription after the intervention compared to six before. Of the six families who had an illness before and after the intervention, three inappropriately used over the counter medications before the study compared to none post intervention. There was one confirmed case of unprescribed antibiotic use pre-intervention, and none post.
Stockwell M.S., *et al.* 2014 [125]<br><br>United States America (USA) Washington Heights, New York City	n= 154 Latino families including 197 children aged 4 years or less recruited from four urban community sites.	Age, race, ethnicity. Location of birth, primary language, English proficiency, education status, health literacy.	The impact of limited health literacy causing increased visits to the paediatric emergency department (PED).	Upper respiratory tract infection (URTI).	Randomised controlled trial evaluating the effectiveness of an educational intervention to decrease PED visits and adverse care practices. Intervention sites received 3 x 1.5-hour education modules focused on the condition, over-the- counter medications, and medication management. Weekly telephone assessments for five months, with reports of URTI in family members, care sought, and medications given. Pre- and post-intervention knowledge-attitude surveys conducted.	Good participation by families who were primarily Latino, Spanish speaking from low-income backgrounds and only 17% had adequate health literacy. Over 94% of families reported data through the study and completed the post intervention study. Total of 396 episodes of infection reported. The intervention group had less PED visits than the group who only received the standard curriculum. The difference was also significant at the family level and there was less risk of obtaining over the counter medication. KAP scores were also higher for the intervention group.
Swe M.M.M., *et al.* 2020 [126]<br><br>Myanmar Yangon	n = 1175 adults from five deprived areas in a large peri urban township.	Overcrowding, poor living conditions, insufficient water, and poor sanitation. Manual and factory workers with below average socioeconomic status.	Testing interventions to increase knowledge about antibiotic behaviour.	Proxy antibiotic use and misuse.	Qualitative public engagement project using a forum theatre to understand antibiotic use for febrile illness and to increase awareness about antibiotic use in the community. Initially five story gathering workshops to develop scripts and songs for the theatre. Plays conducted with four messages based on WHO world antibiotic awareness week advocacy materials. Members of the public invited to intervene in the plays to give the correct answers. Focus group discussions with audience members to evaluate sessions.	Ten forum theatre plays performed on fever and antibiotics and mixed medicines. Thematic analysis showed: 1) Knowledge dissemination prior to the play most did not understand what antibiotics did. Post event aware antibiotics are to kill bacteria. 2) Enjoyment and fun. 3) Willingness to support and recommendations for other events - participants wanted to contribute and include other health topics. 4) Preference for theatre productions rather than traditional methods of health education.

Search strategy, MeSH terms used, and databases data were extracted from

Database:
Ovid MEDLINE(R) and Epub Ahead of Print, In-Process, In-Data-Review & Other Non-Indexed Citations and Daily <1946 to February 15, 2023>

#	Query	Results from 16 Feb 2023
1	inequalit*.mp. [mp=title, book title, abstract, original title, name of substance word, subject heading word, floating sub-heading word, keyword heading word, organism supplementary concept word, protocol supplementary concept word, rare disease supplementary concept word, unique identifier, synonyms]	48,081
2	socioeconomic factor*.mp. or Socioeconomic Factors/	179,102
3	social determinant* of health.mp. [mp=title, book title, abstract, original title, name of substance word, subject heading word, floating sub-heading word, keyword heading word, organism supplementary concept word, protocol supplementary concept word, rare disease supplementary concept word, unique identifier, synonyms]	14,019
4	(cult* determinant* or socio cultural determinant* or cultural value*).mp. or exp Culture/ [mp=title, book title, abstract, original title, name of substance word, subject heading word, floating sub-heading word, keyword heading word, organism supplementary concept word, protocol supplementary concept word, rare disease supplementary concept word, unique identifier, synonyms]	179,002
5	((social* adj1 depriv*) or psychosocial depriv*).mp. [mp=title, book title, abstract, original title, name of substance word, subject heading word, floating sub-heading word, keyword heading word, organism supplementary concept word, protocol supplementary concept word, rare disease supplementary concept word, unique identifier, synonyms]	4,730
6	(Education or school* or tuition* or train* or education* status or (educat* adj2 level)).mp. or exp education/	2,005,870
7	literacy.mp. or exp Literacy/ or exp Health Literacy/ or exp Information Literacy/ or exp Computer Literacy/ or exp internet literacy/ or exp ehealth literacy/	32,720
8	health behavio?r.mp. or exp Health Behavior/	367,039
9	(Level* of income or salary or pay).mp. [mp=title, book title, abstract, original title, name of substance word, subject heading word, floating sub-heading word, keyword heading word, organism supplementary concept word, protocol supplementary concept word, rare disease supplementary concept word, unique identifier, synonyms]	57,344
10	(employment or unemployment or occupation or work* or work* environment).mp. [mp=title, book title, abstract, original title, name of substance word, subject heading word, floating sub-heading word, keyword heading word, organism supplementary concept word, protocol supplementary concept word, rare disease supplementary concept word, unique identifier, synonyms]	2,140,603
11	social behavio?r.mp. or exp Social Behavior/ or exp Interpersonal Relations/ or interpersonal relation*.mp. or personality characteristic*.mp. [mp=title, book title, abstract, original title, name of substance word, subject heading word, floating sub-heading word, keyword heading word, organism supplementary concept word, protocol supplementary concept word, rare disease supplementary concept word, unique identifier, synonyms]	603,740
12	exp social status/ or exp "social aspects and related phenomena"/ or exp social stigma/ or exp social stratification/ or exp social stress/ or (socioeconomic gradient* or socioeconomic gradient*).mp. or social status.mp. or social stigma.mp. or social stratification.mp. or social	27,230

	stress.mp. [mp=title, book title, abstract, original title, name of substance word, subject heading word, floating sub-heading word, keyword heading word, organism supplementary concept word, protocol supplementary concept word, rare disease supplementary concept word, unique identifier, synonyms]	
13	(disadvantage* adj1 social*).mp. [mp=title, book title, abstract, original title, name of substance word, subject heading word, floating sub-heading word, keyword heading word, organism supplementary concept word, protocol supplementary concept word, rare disease supplementary concept word, unique identifier, synonyms]	3,023
14	(relig* or faith or creed).mp. or exp religion/ or buddhism/ or christianity/ or hinduism/ or islam/ or judaism/ or "religion and medicine"/ or "religion and psychology"/	99,293
15	belief.mp. or exp Culture/	214,045
16	exp Poverty Areas/ or poverty.mp. or exp Poverty/ or exp Child Poverty/ or multi* dimensional poverty.mp.	70,546
17	cultural deprivation.mp. or exp Cultural Deprivation/	1,198
18	exp Health Equity/ or health equit*.mp. or exp Healthcare Disparities/ or equit*.mp. or health in?quit*.mp.	64,738
19	exp Life Expectancy/ or life expectan*.mp. or life span.mp. or exp Longevity/	112,512
20	((social* adj1 exclu*) or Social isolation).mp. or exp Social Isolation/	31,866
21	exp "Quality of Life"/ or Social aspect*.mp.	264,110
22	exp social change/ or exp superstitions/ or exp taboo/ or social change*.mp. or superstition*.mp. or taboo.mp.	34,731
23	exp social support/ or exp community support/ or exp psychosocial support systems/ or exp social isolation/ or exp social marginalization/ or exp social norms/ or exp social vulnerability/ or exp socialization/ or exp sociodemographic factors/ or exp Interpersonal Relations/ or Social network*.mp. or exp Social Environment/ or exp Social Networking/	497,475
24	exp gender role/ or gender.mp.	414,200
25	exp social values/ or exp social class/ or exp social mobility/ or exp social factors/ or social class.mp. or social values.mp.	71,228
26	deprivation.mp. or exp food deprivation/ or deprivation.mp. or exp water deprivation/ or index of multiple deprivation.mp.	99,401
27	exp Hygiene/ or exp Sanitation/ or Hygiene.mp. or Sanitation.mp. [mp=title, book title, abstract, original title, name of substance word, subject heading word, floating sub-heading word, keyword heading word, organism supplementary concept word, protocol supplementary concept word, rare disease supplementary concept word, unique identifier, synonyms]	217,320
28	exp residence characteristics/ or exp catchment area, health/ or exp home environment/ or exp housing/ or exp neighborhood characteristics/ or housing.mp.	114,409
29	exp Family Characteristics/ or exp Family Health/ or exp Family Conflict/ or exp Family Relations/ or exp Family/ or famil*.mp.	1,649,433
30	exp Transportation/ or public transport.mp.	78,232
31	exp Hierarchy, Social/ or exp power, psychological/ or exp empowerment/ or intergenerational relations/ or exp maternal behavior/ or exp maternal deprivation/ or exp parent-child relations/ or exp parenting/ or exp paternal behavior/ or exp paternal deprivation/ or exp sibling relations/ or exp family separation/ or exp grandparents/ or exp single-parent family/	106,653
32	exp Environment/ or exp Social Environment/ or Social Environment.mp.	1,619,648
33	stress, psychological/ or financial stress/ or occupational stress/ or stress.mp.	1,124,241
34	(marginali?ed pop* or marginali?ed communit* or marginali?ed people or marginali?ed person or marginali?ed group*).mp. [mp=title, book title, abstract, original title, name of substance	3,902

	word, subject heading word, floating sub-heading word, keyword heading word, organism supplementary concept word, protocol supplementary concept word, rare disease supplementary concept word, unique identifier, synonyms]	
35	(vulnerable pop* or vulnerable person* or vulnerable people or vulnerable communit* or vulnerable group*).mp. or exp african americans/ or exp amish/ or exp arabs/ or exp asian americans/ or exp indigenous peoples/ or exp jews/ or exp roma/ or exp "sexual and gender minorities"/ or exp vulnerable populations/ or exp homebound persons/ or exp homeless persons/ or exp refugees/ [mp=title, book title, abstract, original title, name of substance word, subject heading word, floating sub-heading word, keyword heading word, organism supplementary concept word, protocol supplementary concept word, rare disease supplementary concept word, unique identifier, synonyms]	153,048
36	(disadvant* communit* or disadvant* people or disadvant* person or disadvant* pop* or disadvant* pop*).mp. or exp disadvantaged population/ [mp=title, book title, abstract, original title, name of substance word, subject heading word, floating sub-heading word, keyword heading word, organism supplementary concept word, protocol supplementary concept word, rare disease supplementary concept word, unique identifier, synonyms]	15,211
37	"Transients and Migrants"/ or asylum seek*.mp. or "Emigration and Immigration"/ or (migrant* or immigrant*).mp.	74,909
38	(Gyps* or traveller*).mp. [mp=title, book title, abstract, original title, name of substance word, subject heading word, floating sub-heading word, keyword heading word, organism supplementary concept word, protocol supplementary concept word, rare disease supplementary concept word, unique identifier, synonyms]	13,082
39	exp disabled persons/ or exp amputees/ or exp persons with mental disabilities/ or exp mentally ill persons/ or exp persons with hearing impairments/ or exp visually impaired persons/ or handicap*.mp. or hear* impair*.mp. or hear* loss.mp. or hear* disorder.mp. or deaf.mp. or dumb.mp. or blind*.mp. or visual impair*.mp. [mp=title, book title, abstract, original title, name of substance word, subject heading word, floating sub-heading word, keyword heading word, organism supplementary concept word, protocol supplementary concept word, rare disease supplementary concept word, unique identifier, synonyms]	600,309
40	low income group*.mp.	1,014
41	(Ethnic* or ethnic minorit* or racial minorit* or Asian or black* or ethnic group* or minorit* group* or race* or racial* or people of colo?r or Racial difference*).mp. or exp "Ethnic and Racial Minorities"/ or black.mp. or exp Black person/ or white.mp. or exp Caucasian/ or exp Southeast Asian/ or exp British Asian/ or exp West Asian/ or exp Asian continental ancestry group/ or exp Central Asian/ or exp East Asian/ or exp Asian American/ or exp Asian/ or asian.mp. or exp South Asian/ or mixed race.mp. or Alaska Native.mp. or exp American Indian/ or exp Alaska Native/ or American Indian.mp. or African American.mp. or exp African American/ or Hispanic.mp. or exp Hispanic/ or latino.mp. or Han chinese.mp. or exp Han Chinese/ or people of colo?r.mp. or exp Aborigine/ or Native Hawaiian.mp. or exp Native Hawaiian/ or Pacific Islander.mp. or exp Pacific Islander/	1,047,724
42	Gender.mp. or exp "Sexual and Gender Minorities"/ or Sex* orientation.mp. or exp Gender Identity/ or exp Transsexualism/ or Gender reassignment.mp. or exp Transgender Persons/	422,375
43	urban population.mp. or exp Urban Population/	66,580
44	rural population.mp. or exp Rural Population/	72,842
45	(protected character* or incl* health group*).mp.	24
46	hard to reach.mp.	2,691
47	(health* seek* or health seek* behav* or seek* behav*).mp. [mp=title, book title, abstract, original title, name of substance word, subject heading word, floating sub-heading word,	20,480

	keyword heading word, organism supplementary concept word, protocol supplementary concept word, rare disease supplementary concept word, unique identifier, synonyms]	
48	(health* access* or health care access* or health* service or access to health*).mp. or exp health insurance/ or exp health care access/	241,447
49	exp "Patient Acceptance of Health Care"/ or exp Help-Seeking Behavior/ or Help seek*behavio?r.mp.	172,189
50	Treatment* seeking behav*.mp.	857
51	"delivery of health care"/ or exp after-hours care/ or exp culturally competent care/ or exp delegation, professional/ or exp "delivery of health care, integrated"/ or exp health services accessibility/ or delivery of health care.mp.	253,450
52	exp Health Promotion/ or health promotion*.mp.	107,721
53	exp Health Services Accessibility/ or Human right*.mp.	146,695
54	health* provision.mp.	1,977
55	(healthcare staff or med* care or nurs* care or multidisc* team or health* work* or doctor* or Surgeon or physician* or Nurse* or pharmacist* or interdisciplin*).mp. [mp=title, book title, abstract, original title, name of substance word, subject heading word, floating sub-heading word, keyword heading word, organism supplementary concept word, protocol supplementary concept word, rare disease supplementary concept word, unique identifier, synonyms]	1,404,066
56	exp medicine, traditional/ or exp medicine, african traditional/ or exp medicine, arabic/ or exp medicine, ayurvedic/ or exp medicine, east asian traditional/ or exp medicine, persian/ or exp shamanism/ or traditional medicine.mp.	56,160
57	allopathic.mp.	2,113
58	preventative health*.mp.	774
59	Access to Information/ or "Access to Information".mp.	11,776
60	internet access.mp. or Internet Access/ or Patient Education as Topic/	90,275
61	Health* facilit*.mp. or exp Health Facilities/	908,605
62	Social Perception/ or Perception*.mp.	514,986
63	exp Rural Health Services/ or rural.mp. or exp Rural Health/	198,121
64	urban health.mp. or exp Urban Health/	23,910
65	exp "referral and consultation"/ or exp remote consultation/ or referral*.mp. or consult*.mp. [mp=title, book title, abstract, original title, name of substance word, subject heading word, floating sub-heading word, keyword heading word, organism supplementary concept word, protocol supplementary concept word, rare disease supplementary concept word, unique identifier, synonyms]	328,804
66	exp Health Behavior/ or behavio?r change.mp. or behavio?r intervention*.mp. [mp=title, book title, abstract, original title, name of substance word, subject heading word, floating sub-heading word, keyword heading word, organism supplementary concept word, protocol supplementary concept word, rare disease supplementary concept word, unique identifier, synonyms]	373,471
67	(co* produc* or public engagement or patient engagement).mp. or exp Patient Participation/ [mp=title, book title, abstract, original title, name of substance word, subject heading word, floating sub-heading word, keyword heading word, organism supplementary concept word, protocol supplementary concept word, rare disease supplementary concept word, unique identifier, synonyms]	162,820
68	community participation.mp. or exp Community Participation/	50,336
69	Health Education/ or Health Knowledge, Attitudes, Practice/	179,924
70	exp Attitude to Health/ or belief system.mp. or myth.mp.	474,809

71	Cultural Competency/ or exp "Attitude of Health Personnel"/ or exp Culturally Competent Care/ or exp Cultural Characteristics/ or Cultur* practice*.mp.	194,601
72	Anti microbial stewardship.mp. or exp Antimicrobial Stewardship/	3,257
73	(infection control or infection prevention control).mp. [mp=title, book title, abstract, original title, name of substance word, subject heading word, floating sub-heading word, keyword heading word, organism supplementary concept word, protocol supplementary concept word, rare disease supplementary concept word, unique identifier, synonyms]	49,068
74	antibiotic resistance.mp. or exp Drug Resistance, Microbial/	206,633
75	((antimicrob* adj2 resist*) or anti microb* resist* or AMR).mp.	42,704
76	Drug resistance , multiple, bacterial.mp. or exp Drug Resistance, Multiple, Bacterial/	25,227
77	(antibact* resist* or anti bact* resist* or ABR).mp. [mp=title, book title, abstract, original title, name of substance word, subject heading word, floating sub-heading word, keyword heading word, organism supplementary concept word, protocol supplementary concept word, rare disease supplementary concept word, unique identifier, synonyms]	7,719
78	exp Anti-Bacterial Agents/ or Anti* bacteria* agent*.mp. or anti* infective.mp. or antimicrobial agent*.mp. or antibiotic*.mp. [mp=title, book title, abstract, original title, name of substance word, subject heading word, floating sub-heading word, keyword heading word, organism supplementary concept word, protocol supplementary concept word, rare disease supplementary concept word, unique identifier, synonyms]	1,071,207
79	Streptococ* pneumoniae infec*.mp. or exp Streptococ* Infections/ or streptococ* infec.mp. or exp Streptococcus pyogenes infec*/ or strep* infec*.mp. or exp Pneumococcal Infections/ or Pneumococ* infec*.mp.	59,266
80	(Staphylococ* infec* or Vancomycin-Resistant Staphylococcus aureus infec).mp. or exp Staphylococcus aureus infec*/ or MRSA infec*.mp. or methicillin* resist* staph* aureus infec*.mp. [mp=title, book title, abstract, original title, name of substance word, subject heading word, floating sub-heading word, keyword heading word, organism supplementary concept word, protocol supplementary concept word, rare disease supplementary concept word, unique identifier, synonyms]	67,361
81	exp Gram-Positive Bacterial Infections/ or gram positive infec*.mp.	468,880
82	gram negative infec*.mp. or exp Gram-Negative Bacterial Infections/	396,700
83	clostridium infec*.mp. or exp Clostridium Infections/	32,444
84	(carbapenemase-producing enterobacteriaceae infec* or enterobacteriaceae infections).mp. or exp enterobacteriaceae infections/	109,675
85	Vancomycin resistant enterococ* infec*.mp.	136
86	exp Acinetobacter Infections/ or Acinobacter infec*.mp.	5,329
87	exp Surgical Wound Infection/ or Surg* infec*.mp.	41,656
88	exp Skin Diseases, Bacterial/ or skin infection.mp. or exp Skin Diseases, Infectious/	128,093
89	soft tissue infection.mp. or exp Soft Tissue Infections/	6,741
90	exp Sepsis/ or sepsis.mp.	207,151
91	exp Bacteremia/ or bacter?emia.mp.	52,153
92	exp Urinary Tract Infections/ or urinary tract infect*.mp.	73,349
93	Nosocomial infec*.mp.	17,287
94	(hospital acquired infec* or hospital associated infec* or community acquired infec*).mp. [mp=title, book title, abstract, original title, name of substance word, subject heading word, floating sub-heading word, keyword heading word, organism supplementary concept word, protocol supplementary concept word, rare disease supplementary concept word, unique identifier, synonyms]	22,703

95	exp Respiratory Tract Infections/ or Resp* infec*.mp. or chest infec*.mp. or pul* infec*.mp. or resp* tract infec*.mp. or pneumonia.mp. [mp=title, book title, abstract, original title, name of substance word, subject heading word, floating sub-heading word, keyword heading word, organism supplementary concept word, protocol supplementary concept word, rare disease supplementary concept word, unique identifier, synonyms]	698,484
96	klebsiella.mp. or exp Klebsiella Infections/	46,537
97	Pseudomonas infec*.mp. or Pseudomonas Infections/	22,689
98	E coli infec*.mp. or exp Escherichia coli Infections/	36,049
99	1 or 2 or 3 or 4 or 5 or 6 or 7 or 8 or 9 or 10 or 11 or 12 or 13 or 14 or 15 or 16 or 17 or 18 or 19 or 20 or 21 or 22 or 23 or 24 or 25 or 26 or 27 or 28 or 29 or 30 or 31 or 32 or 33	8,656,901
100	34 or 35 or 36 or 37 or 38 or 39 or 40 or 41 or 42 or 43 or 44 or 45 or 46	2,183,961
101	47 or 48 or 49 or 50 or 51 or 52 or 53 or 54 or 55 or 56 or 57 or 58 or 59 or 60 or 61 or 62 or 63 or 64 or 65 or 66 or 67 or 68 or 69 or 70 or 71	3,866,334
102	74 or 75 or 76 or 77	233,361
103	72 or 73 or 78 or 79 or 80 or 81 or 82 or 83 or 84 or 85 or 86 or 87 or 88 or 89 or 90 or 91 or 92 or 93 or 94 or 95 or 96 or 97 or 98	2,498,006
104	102 and 103	185,457
105	99 and 100 and 101 and 104	740
106	exp animals/ not humans.sh.	5,093,590
107	105 not 106	732
108	limit 107 to yr="2000 - 2022"	648

inequalit*.mp. [mp=title, book title, abstract, original title, name of substance word, subject heading word, floating sub-heading word, keyword heading word, organism supplementary concept word, protocol supplementary concept word, rare disease supplementary concept word, unique identifier, synonyms]
socioeconomic factor*.mp. or Socioeconomic Factors/
social determinant* of health.mp. [mp=title, book title, abstract, original title, name of substance word, subject heading word, floating sub-heading word, keyword heading word, organism supplementary concept word, protocol supplementary concept word, rare disease supplementary concept word, unique identifier, synonyms]
(cult* determinant* or socio cultural determinant* or cultural value*).mp. or exp Culture/ [mp=title, book title, abstract, original title, name of substance word, subject heading word, floating sub-heading word, keyword heading word, organism supplementary concept word, protocol supplementary concept word, rare disease supplementary concept word, unique identifier, synonyms]
((social* adj1 depriv*) or psychosocial depriv*).mp. [mp=title, book title, abstract, original title, name of substance word, subject heading word, floating sub-heading word, keyword heading word, organism supplementary concept word, protocol supplementary concept word, rare disease supplementary concept word, unique identifier, synonyms]
(Education or school* or tuition* or train* or education* status or (educat* adj2 level)).mp. or exp education/
literacy.mp. or exp Literacy/ or exp Health Literacy/ or exp Information Literacy/ or exp Computer Literacy/ or exp internet literacy/ or exp ehealth literacy/
health behavio?r.mp. or exp Health Behavior/
(Level* of income or salary or pay).mp. [mp=title, book title, abstract, original title, name of substance word, subject heading word, floating sub-heading word, keyword heading word, organism supplementary concept word, protocol supplementary concept word, rare disease supplementary

concept word, unique identifier, synonyms]
(employment or unemployment or occupation or work* or work* environment).mp. [mp=title, book title, abstract, original title, name of substance word, subject heading word, floating sub-heading word, keyword heading word, organism supplementary concept word, protocol supplementary concept word, rare disease supplementary concept word, unique identifier, synonyms]
social behavio?r.mp. or exp Social Behavior/ or exp Interpersonal Relations/ or interpersonal relation*.mp. or personality characteristic*.mp. [mp=title, book title, abstract, original title, name of substance word, subject heading word, floating sub-heading word, keyword heading word, organism supplementary concept word, protocol supplementary concept word, rare disease supplementary concept word, unique identifier, synonyms]
exp social status/ or exp "social aspects and related phenomena"/ or exp social stigma/ or exp social stratification/ or exp social stress/ or (socioeconomic gradient* or socioeconomic gradient*).mp. or social status.mp. or social stigma.mp. or social stratification.mp. or social stress.mp. [mp=title, book title, abstract, original title, name of substance word, subject heading word, floating sub-heading word, keyword heading word, organism supplementary concept word, protocol supplementary concept word, rare disease supplementary concept word, unique identifier, synonyms]
(disadvantage* adj1 social*).mp. [mp=title, book title, abstract, original title, name of substance word, subject heading word, floating sub-heading word, keyword heading word, organism supplementary concept word, protocol supplementary concept word, rare disease supplementary concept word, unique identifier, synonyms]
(relig* or faith or creed).mp. or exp religion/ or buddhism/ or christianity/ or hinduism/ or islam/ or judaism/ or "religion and medicine"/ or "religion and psychology"/
belief.mp. or exp Culture/
exp Poverty Areas/ or poverty.mp. or exp Poverty/ or exp Child Poverty/ or multi* dimensional poverty.mp.
cultural deprivation.mp. or exp Cultural Deprivation/
exp Health Equity/ or health equit*.mp. or exp Healthcare Disparities/ or equit*.mp. or health in?quit*.mp.
exp Life Expectancy/ or life expectan*.mp. or life span.mp. or exp Longevity/
((social* adj1 exclu*) or Social isolation).mp. or exp Social Isolation/
exp "Quality of Life"/ or Social aspect*.mp.
exp social change/ or exp superstitions/ or exp taboo/ or social change*.mp. or superstition*.mp. or taboo.mp.
exp social support/ or exp community support/ or exp psychosocial support systems/ or exp social isolation/ or exp social marginalization/ or exp social norms/ or exp social vulnerability/ or exp socialization/ or exp sociodemographic factors/ or exp Interpersonal Relations/ or Social network*.mp. or exp Social Environment/ or exp Social Networking/
exp gender role/ or gender.mp.
exp social values/ or exp social class/ or exp social mobility/ or exp social factors/ or social class.mp. or social values.mp.
deprivation.mp. or exp food deprivation/ or deprivation.mp. or exp water deprivation/ or index of multiple deprivation.mp.
exp Hygiene/ or exp Sanitation/ or Hygiene.mp. or Sanitation.mp. [mp=title, book title, abstract, original title, name of substance word, subject heading word, floating sub-heading word, keyword heading word, organism supplementary concept word, protocol supplementary concept word, rare disease supplementary concept word, unique identifier, synonyms]
exp residence characteristics/ or exp catchment area, health/ or exp home environment/ or exp housing/ or exp neighborhood characteristics/ or housing.mp.
exp Family Characteristics/ or exp Family Health/ or exp Family Conflict/ or exp Family Relations/ or exp Family/ or famil*.mp.
exp Transportation/ or public transport.mp.

exp Hierarchy, Social/ or exp power, psychological/ or exp empowerment/ or intergenerational relations/ or exp maternal behavior/ or exp maternal deprivation/ or exp parent-child relations/ or exp parenting/ or exp paternal behavior/ or exp paternal deprivation/ or exp sibling relations/ or exp family separation/ or exp grandparents/ or exp single-parent family/
exp Environment/ or exp Social Environment/ or Social Environment.mp.
stress, psychological/ or financial stress/ or occupational stress/ or stress.mp.
(marginali?ed pop* or marginali?ed communit* or marginali?ed people or marginali?ed person or marginali?ed group*).mp. [mp=title, book title, abstract, original title, name of substance word, subject heading word, floating sub-heading word, keyword heading word, organism supplementary concept word, protocol supplementary concept word, rare disease supplementary concept word, unique identifier, synonyms]
(vulnerable pop* or vulnerable person* or vulnerable people or vulnerable communit* or vulnerable group*).mp. or exp african americans/ or exp amish/ or exp arabs/ or exp asian americans/ or exp indigenous peoples/ or exp jews/ or exp roma/ or exp "sexual and gender minorities"/ or exp vulnerable populations/ or exp homebound persons/ or exp homeless persons/ or exp refugees/ [mp=title, book title, abstract, original title, name of substance word, subject heading word, floating sub-heading word, keyword heading word, organism supplementary concept word, protocol supplementary concept word, rare disease supplementary concept word, unique identifier, synonyms]
(disadvant* communit* or disadvant* people or disadvant* person or disadvant* pop* or disadvant* pop*).mp. or exp disadvantaged population/ [mp=title, book title, abstract, original title, name of substance word, subject heading word, floating sub-heading word, keyword heading word, organism supplementary concept word, protocol supplementary concept word, rare disease supplementary concept word, unique identifier, synonyms]
"Transients and Migrants"/ or asylum seek*.mp. or "Emigration and Immigration"/ or (migrant* or immigrant*).mp.
(Gyps* or traveller*).mp. [mp=title, book title, abstract, original title, name of substance word, subject heading word, floating sub-heading word, keyword heading word, organism supplementary concept word, protocol supplementary concept word, rare disease supplementary concept word, unique identifier, synonyms]
exp disabled persons/ or exp amputees/ or exp persons with mental disabilities/ or exp mentally ill persons/ or exp persons with hearing impairments/ or exp visually impaired persons/ or handicap*.mp. or hear* impair*.mp. or hear* loss.mp. or hear* disorder.mp. or deaf.mp. or dumb.mp. or blind*.mp. or visual impair*.mp. [mp=title, book title, abstract, original title, name of substance word, subject heading word, floating sub-heading word, keyword heading word, organism supplementary concept word, protocol supplementary concept word, rare disease supplementary concept word, unique identifier, synonyms]
low income group*.mp.
(Ethnic* or ethnic minorit* or racial minorit* or Asian or black* or ethnic group* or minorit* group* or race* or racial* or people of colo?r or Racial difference*).mp. or exp "Ethnic and Racial Minorities"/ or black.mp. or exp Black person/ or white.mp. or exp Caucasian/ or exp Southeast Asian/ or exp British Asian/ or exp West Asian/ or exp Asian continental ancestry group/ or exp Central Asian/ or exp East Asian/ or exp Asian American/ or exp Asian/ or asian.mp. or exp South Asian/ or mixed race.mp. or Alaska Native.mp. or exp American Indian/ or exp Alaska Native/ or American Indian.mp. or African American.mp. or exp African American/ or Hispanic.mp. or exp Hispanic/ or latino.mp. or Han chinese.mp. or exp Han Chinese/ or people of colo?r.mp. or exp Aborigine/ or Native Hawaiian.mp. or exp Native Hawaiian/ or Pacific Islander.mp. or exp Pacific Islander/
Gender.mp. or exp "Sexual and Gender Minorities"/ or Sex* orientation.mp. or exp Gender Identity/ or exp Transsexualism/ or Gender reassignment.mp. or exp Transgender Persons/
urban population.mp. or exp Urban Population/
rural population.mp. or exp Rural Population/
(protected character* or incl* health group*).mp.

hard to reach.mp.
(health* seek* or health seek* behav* or seek* behav*).mp. [mp=title, book title, abstract, original title, name of substance word, subject heading word, floating sub-heading word, keyword heading word, organism supplementary concept word, protocol supplementary concept word, rare disease supplementary concept word, unique identifier, synonyms]
(health* access* or health care access* or health* service or access to health*).mp. or exp health insurance/ or exp health care access/
exp "Patient Acceptance of Health Care"/ or exp Help-Seeking Behavior/ or Help seek*behavio?r.mp.
Treatment* seeking behav*.mp.
"delivery of health care"/ or exp after-hours care/ or exp culturally competent care/ or exp delegation, professional/ or exp "delivery of health care, integrated"/ or exp health services accessibility/ or delivery of health care.mp.
exp Health Promotion/ or health promotion*.mp.
exp Health Services Accessibility/ or Human right*.mp.
health* provision.mp.
(healthcare staff or med* care or nurs* care or multidisc* team or health* work* or doctor* or Surgeon or physician* or Nurse* or pharmacist* or interdisciplin*).mp. [mp=title, book title, abstract, original title, name of substance word, subject heading word, floating sub-heading word, keyword heading word, organism supplementary concept word, protocol supplementary concept word, rare disease supplementary concept word, unique identifier, synonyms]
exp medicine, traditional/ or exp medicine, african traditional/ or exp medicine, arabic/ or exp medicine, ayurvedic/ or exp medicine, east asian traditional/ or exp medicine, persian/ or exp shamanism/ or traditional medicine.mp.
allopathic.mp.
preventative health*.mp.
Access to Information/ or "Access to Information".mp.
internet access.mp. or Internet Access/ or Patient Education as Topic/
Health* facilit*.mp. or exp Health Facilities/
Social Perception/ or Perception*.mp.
exp Rural Health Services/ or rural.mp. or exp Rural Health/
urban health.mp. or exp Urban Health/
exp "referral and consultation"/ or exp remote consultation/ or referral*.mp. or consult*.mp. [mp=title, book title, abstract, original title, name of substance word, subject heading word, floating sub-heading word, keyword heading word, organism supplementary concept word, protocol supplementary concept word, rare disease supplementary concept word, unique identifier, synonyms]
exp Health Behavior/ or behavio?r change.mp. or behavio?r intervention*.mp. [mp=title, book title, abstract, original title, name of substance word, subject heading word, floating sub-heading word, keyword heading word, organism supplementary concept word, protocol supplementary concept word, rare disease supplementary concept word, unique identifier, synonyms]
(co* produc* or public engagement or patient engagement).mp. or exp Patient Participation/ [mp=title, book title, abstract, original title, name of substance word, subject heading word, floating sub-heading word, keyword heading word, organism supplementary concept word, protocol supplementary concept word, rare disease supplementary concept word, unique identifier, synonyms]
community participation.mp. or exp Community Participation/
Health Education/ or Health Knowledge, Attitudes, Practice/
exp Attitude to Health/ or belief system.mp. or myth.mp.
Cultural Competency/ or exp "Attitude of Health Personnel"/ or exp Culturally Competent Care/ or exp Cultural Characteristics/ or Cultur* practice*.mp.
Anti microbial stewardship.mp. or exp Antimicrobial Stewardship/
(infection control or infection prevention control).mp. [mp=title, book title, abstract, original title, name of substance word, subject heading word, floating sub-heading word, keyword heading word, organism

supplementary concept word, protocol supplementary concept word, rare disease supplementary concept word, unique identifier, synonyms]
antibiotic resistance.mp. or exp Drug Resistance, Microbial/
((antimicrob* adj2 resist*) or anti microb* resist* or AMR).mp.
Drug resistance , multiple, bacterial.mp. or exp Drug Resistance, Multiple, Bacterial/
(antibact* resist* or anti bact* resist* or ABR).mp. [mp=title, book title, abstract, original title, name of substance word, subject heading word, floating sub-heading word, keyword heading word, organism supplementary concept word, protocol supplementary concept word, rare disease supplementary concept word, unique identifier, synonyms]
exp Anti-Bacterial Agents/ or Anti* bacteria* agent*.mp. or anti* infective.mp. or antimicrobial agent*.mp. or antibiotic*.mp. [mp=title, book title, abstract, original title, name of substance word, subject heading word, floating sub-heading word, keyword heading word, organism supplementary concept word, protocol supplementary concept word, rare disease supplementary concept word, unique identifier, synonyms]
Streptococ* pneumoniae infec*.mp. or exp Streptococ* Infections/ or streptococ* infec.mp. or exp Streptococcus pyogenes infec*/ or strep* infec*.mp. or exp Pneumococcal Infections/ or Pneumococ* infec*.mp.
(Staphylococ* infec* or Vancomycin-Resistant Staphylococcus aureus infec).mp. or exp Staphylococcus aureus infec*/ or MRSA infec*.mp. or methicillin* resist* staph* aureus infec*.mp. [mp=title, book title, abstract, original title, name of substance word, subject heading word, floating sub-heading word, keyword heading word, organism supplementary concept word, protocol supplementary concept word, rare disease supplementary concept word, unique identifier, synonyms]
exp Gram-Positive Bacterial Infections/ or gram positive infec*.mp.
gram negative infec*.mp. or exp Gram-Negative Bacterial Infections/
clostridium infec*.mp. or exp Clostridium Infections/
(carbapenemase-producing enterobacteriaceae infec* or enterobacteriaceae infections).mp. or exp enterobacteriaceae infections/
Vancomycin resistant enterococ* infec*.mp.
exp Acinetobacter Infections/ or Acinobacter infec*.mp.
exp Surgical Wound Infection/ or Surg* infec*.mp.
exp Skin Diseases, Bacterial/ or skin infection.mp. or exp Skin Diseases, Infectious/
soft tissue infection.mp. or exp Soft Tissue Infections/
exp Sepsis/ or sepsis.mp.
exp Bacteremia/ or bacter?emia.mp.
exp Urinary Tract Infections/ or urinary tract infect*.mp.
Nosocomial infec*.mp.
(hospital acquired infec* or hospital associated infec* or community acquired infec*).mp. [mp=title, book title, abstract, original title, name of substance word, subject heading word, floating sub-heading word, keyword heading word, organism supplementary concept word, protocol supplementary concept word, rare disease supplementary concept word, unique identifier, synonyms]
exp Respiratory Tract Infections/ or Resp* infec*.mp. or chest infec*.mp. or pul* infec*.mp. or resp* tract infec*.mp. or pneumonia.mp. [mp=title, book title, abstract, original title, name of substance word, subject heading word, floating sub-heading word, keyword heading word, organism supplementary concept word, protocol supplementary concept word, rare disease supplementary concept word, unique identifier, synonyms]
klebsiella.mp. or exp Klebsiella Infections/
Pseudomonas infec*.mp. or Pseudomonas Infections/
E coli infec*.mp. or exp Escherichia coli Infections/
1 or 2 or 3 or 4 or 5 or 6 or 7 or 8 or 9 or 10 or 11 or 12 or 13 or 14 or 15 or 16 or 17 or 18 or 19 or 20 or 21 or 22 or 23 or 24 or 25 or 26 or 27 or 28 or 29 or 30 or 31 or 32 or 33
34 or 35 or 36 or 37 or 38 or 39 or 40 or 41 or 42 or 43 or 44 or 45 or 46

47 or 48 or 49 or 50 or 51 or 52 or 53 or 54 or 55 or 56 or 57 or 58 or 59 or 60 or 61 or 62 or 63 or 64 or 65 or 66 or 67 or 68 or 69 or 70 or 71
74 or 75 or 76 or 77
72 or 73 or 78 or 79 or 80 or 81 or 82 or 83 or 84 or 85 or 86 or 87 or 88 or 89 or 90 or 91 or 92 or 93 or 94 or 95 or 96 or 97 or 98
102 and 103
99 and 100 and 101 and 104
exp animals/ not humans.sh.
105 not 106
limit 107 to yr="2000 - 2022"

https://ovidsp.ovid.com/ovidweb.cgi?T=JS&NEWS=N&PAGE=main&SHAREDSEARCHID=72l3aDGfDBapRxt0WANsCBrU8mQuwj4wF1Sbesq18NCkHCWHtzhUdzzGP4bZx5RaX

Table 8 Intra -rater reliability Cohen Kapa score

Reviewer A	Reviewer B	A Yes, B Yes	A Yes, B No	A No, B Yes	A No, B No	Proportionate Agreement	Yes Probability	No Probability	Random Agreement Probability	Cohen's Kappa
AES	EC	143	321	91	3906	0.90764	0.00546	0.84899	0.85444	0.36549
AG	EC	46	69	59	5135	0.97589	0.00043	0.95899	0.95942	0.4059
EC	EC1	2	3	2	306	0.98403	0.0002	0.97145	0.97165	0.43644
AES	AG	24	107	10	4366	0.97404	0.00022	0.96361	0.96383	0.28231
AES	EC1	0	10	1	1196	0.99089	0.00001	0.99089	0.9909	-0.00151

Table 8: Intra -rater reliability Cohen Kapa score for the reviewers

References

1. Afari-Asiedu S., Oppong F. B., Tostmann A., et al. Determinants of Inappropriate Antibiotics Use in Rural Central Ghana Using a Mixed Methods Approach. *Front Public Health* 2020;8(101616579):90.doi:https://doi.org/10.3389/fpubh.2020.00090[published Online First: 20200324]

2. Babu G., Balamuruganvelu S., Reddy S. V., et al. Bacteriological Profile of Wounds Due to Occupational Injuries among Fisherman Community of Puducherry, India. *J Clin Diagn Res* 2018;12(10):Dc11-Dc15.doi:https://doi.org/10.7860/JCDR/2018/37409.12184

3. Boo Y. Y., Rai K., Cupp M. A., et al. What Are the Determinants of Childhood Infections in India's Peri-Urban Slums? A Case Study of Eight Cities. *PLoS One* 2021;16(10):e0257797.doi:https://doi.org/10.1371/journal.pone.0257797[published Online First: 20211015]

4. Casey J. A., Rudolph K. E., Robinson S. C., et al. Sociodemographic Inequalities in Urinary Tract Infection in 2 Large California Health Systems. *Open Forum Infect Dis* 2021;8(6):ofab276.doi:https://doi.org/10.1093/ofid/ofab276[published Online First: 20210526]

5. Caudell M. A., Mair C., Subbiah M., et al. Identification of Risk Factors Associated with Carriage of Resistant Escherichia Coli in Three Culturally Diverse Ethnic Groups in Tanzania: A Biological and Socioeconomic Analysis. *Lancet Planet Health* 2018;2(11):e489-e97.doi:https://doi.org/10.1016/S2542-5196(18)30225-0

6. Covvey J. R., Johnson B. F., Elliott V., et al. An Association between Socioeconomic Deprivation and Primary Care Antibiotic Prescribing in Scotland. *J Antimicrob Chemother* 2014;69(3):835-41.doi:https://doi.org/10.1093/jac/dkt439

[published Online First: 20131031]

7. See I., Wesson P., Gualandi N., et al. Socioeconomic Factors Explain Racial Disparities in Invasive Community-Associated Methicillin-Resistant Staphylococcus Aureus Disease Rates. *Clinical infectious diseases : an official publication of the Infectious Diseases Society of America* 2017;64(5):597-604.doi:https://dx.doi.org/10.1093/cid/ciw808

8. Svalestuen S., Svendsen K., Eggen A. E., et al. Association of Area-Level Education with the Regional Growth Trajectories of Rates of Antibacterial Dispensing to Patients under 3 Years in Norway: A Longitudinal Retrospective Study. *BMJ Open* 2022;12(9):e058491.doi:https://doi.org/10.1136/bmjopen-2021-058491[published Online First: 20220908]

9. Thomson K., Berry R., Robinson T., et al. An Examination of Trends in Antibiotic Prescribing in Primary Care and the Association with Area-Level Deprivation in England. *BMC Public Health* 2020;20(1):1148.doi:https://doi.org/10.1186/s12889-020-09227-x[published Online First: 20200803]

10. Tosas Auguet O., Betley J. R., Stabler R. A., et al. Evidence for Community Transmission of Community-Associated but Not Health-Care-Associated Methicillin-Resistant Staphylococcus Aureus Strains Linked to Social and Material Deprivation: Spatial Analysis of Cross-Sectional Data. *PLoS Med*

2016;13(1):e1001944.doi:https://doi.org/10.1371/journal.pmed.1001944[published Online First: 20160126]

11. Walls G., Vandal A. C., du Plessis T., et al. Socioeconomic Factors Correlating with Community Antimicrobial Prescribing. *N Z Med J* 2015;128(1417):16-23.doi:https://www.ncbi.nlm.nih.gov/pubmed/26149899[published Online First: 20150703]

12. Whyler N., Tomlin A., Tilyard M., et al. Ethnic Disparities in Community Antibacterial Dispensing in New Zealand, 2015. *N Z Med J* 2018;131(1480):50-60.doi:https://www.ncbi.nlm.nih.gov/pubmed/30116065[published Online First: 20180817]

13. Sokontheavy Y., Kittipong S., Wongsa L. Inappropriate Use of Antibiotics among Children under Five in Rural and Urban Communities of Cambodia. *Indian Journal of Forensic Medicine & Toxicology* 2021;16(1):1126-35.doi:https://doi.org/10.37506/ijfmt.v16i1.17647

14. Zheng C., Karkey A., Wang T., et al. Determinants and Patterns of Antibiotic Consumption for Children under Five in Nepal: Analysis and Modelling of Demographic Health Survey Data from 2006 to 2016. *Trop Med Int Health* 2021;26(4):397-409.doi:https://doi.org/10.1111/tmi.13540[published Online First: 20210122]

15. Afari-Asiedu S., Hulscher M., Abdulai M. A., et al. Every Medicine Is Medicine; Exploring Inappropriate Antibiotic Use at the Community Level in Rural Ghana. *BMC Public Health* 2020;20(1):1103.doi:https://doi.org/10.1186/s12889-020-09204-4[published Online First: 20200714]

16. Al Baz M., Law M. R., Saadeh R. Antibiotics Use among Palestine Refugees Attending Unrwa Primary Health Care Centers in Jordan - a Cross-Sectional Study. *Travel Med Infect Dis* 2018;22(101230758):25-29.doi:https://doi.org/10.1016/j.tmaid.2018.02.004[published Online First: 20180216]

17. Alkirawan R., Kawous R., Bloemen E., et al. Perspectives of Syrian Refugees on Antibiotic Use and Prescribing in Dutch Primary Care: A Qualitative Study. *International Journal of Migration Health and Social Care* 2022;18(2):153-63.doi:https://doi.org/10.1108/Ijmhsc-12-2021-0112

18. Barker A. K., Brown K., Ahsan M., et al. Social Determinants of Antibiotic Misuse: A Qualitative Study of Community Members in Haryana, India. *BMC Public Health* 2017;17(1):333.doi:https://doi.org/10.1186/s12889-017-4261-4[published Online First: 20170419]

19. Bernadas J. M. A. C. Antibiotic-Related Meanings, Experiences and Information Sources of Women in the Economic Margins of Urban Manila. *International Journal of Human Rights in Health Care* 2019;12(1):3-15.doi:https://doi.org/10.1108/Ijhrh-06-2018-0039

20. Bogale A. A., Amhare A. F., Chang J., et al. Knowledge, Attitude, and Practice of Self-Medication with Antibiotics among Community Residents in Addis Ababa, Ethiopia. *Expert Rev Anti Infect Ther* 2019;17(6):459-66.doi:https://doi.org/10.1080/14787210.2019.1620105[published Online First: 20190524]

21. Burtscher D., Van den Bergh R., Nasim M., et al. 'They Eat It Like Sweets': A Mixed Methods Study of Antibiotic Perceptions and Their Use among Patients, Prescribers and Pharmacists in a District Hospital in Kabul, Afghanistan. *PLoS One*

2021;16(11):e0260096.doi:https://doi.org/10.1371/journal.pone.0260096[published Online First: 20211119]

22. Cheng J., Coope C., Chai J., et al. Knowledge and Behaviors in Relation to Antibiotic Use among Rural Residents in Anhui, China. *Pharmacoepidemiol Drug Saf* 2018;27(6):652-59.doi:https://doi.org/10.1002/pds.4429[published Online First: 20180326]

23. Corbett K. K., Gonzales R., Leeman-Castillo B. A., et al. Appropriate Antibiotic Use: Variation in Knowledge and Awareness by Hispanic Ethnicity and Language. *Prev Med* 2005;40(2):162-9.doi:https://doi.org/10.1016/j.ypmed.2004.05.016

24. Crigger N. J., Holcomb L., Grogan R. L., et al. Development of the Choices and Acquisition of Antibiotics Model from a Descriptive Study of a Lay Honduran Population. *Int J Nurs Stud* 2004;41(7):745-53.doi:https://doi.org/10.1016/j.ijnurstu.2004.03.001

25. Dunn-Navarra A. M., Stockwell M. S., Meyer D., et al. Parental Health Literacy, Knowledge and Beliefs Regarding Upper Respiratory Infections (Uri) in an Urban Latino Immigrant Population. *J Urban Health* 2012;89(5):848-60.doi:https://doi.org/10.1007/s11524-012-9692-8

26. Emgard M., Mwangi R., Mayo C., et al. Antibiotic Use in Children under 5 Years of Age in Northern Tanzania: A Qualitative Study Exploring the Experiences of the Caring Mothers. *Antimicrob Resist Infect Control* 2022;11(1):130.doi:https://doi.org/10.1186/s13756-022-01169-w[published Online First: 20221103]

27. Francois Watkins L. K., Sanchez G. V., Albert A. P., et al. Knowledge and Attitudes Regarding Antibiotic Use among Adult Consumers, Adult Hispanic Consumers, and Health Care Providers--United States, 2012-2013. *MMWR Morb Mortal Wkly Rep* 2015;64(28):767-70.doi:https://doi.org/10.15585/mmwr.mm6428a5

28. Gebeyehu E., Bantie L., Azage M. Inappropriate Use of Antibiotics and Its Associated Factors among Urban and Rural Communities of Bahir Dar City Administration, Northwest Ethiopia. *PLoS One* 2015;10(9):e0138179.doi:https://doi.org/10.1371/journal.pone.0138179[published Online First: 20150917]

29. Geta K., Kibret M. Knowledge, Attitudes and Practices of Patients on Antibiotic Resistance and Use in Public Hospitals of Amhara Regional State, Northwestern Ethiopia: A Cross-Sectional Study. *Infect Drug Resist* 2022;15:193-209.doi:https://doi.org/10.2147/IDR.S348765[published Online First: 20220122]

30. Gunasekera Y. D., Kinnison T., Kottawatta S. A., et al. Misconceptions of Antibiotics as a Potential Explanation for Their Misuse. A Survey of the General Public in a Rural and Urban Community in Sri Lanka. *Antibiotics (Basel)* 2022;11(4):454.doi:https://doi.org/10.3390/antibiotics11040454[published Online First: 20220327]

31. Ha T. V., Nguyen A. M. T., Nguyen H. S. T. Public Awareness About Antibiotic Use and Resistance among Residents in Highland Areas of Vietnam. *Biomed Res Int* 2019;2019(101600173):9398536.doi:https://doi.org/10.1155/2019/9398536[published Online First: 20190516]

32. Haenssgen M. J., Charoenboon N., Zanello G., et al. Antibiotic Knowledge, Attitudes and Practices: New Insights from Cross-Sectional Rural Health Behaviour Surveys in Low-Income and

Middle-Income South-East Asia. *BMJ Open* 2019;9(8):e028224.doi:https://doi.org/10.1136/bmjopen-2018-028224[published Online First: 20190820]

33. Halfvarsson J., Heijne N., Ljungman P., et al. Knowing When but Not How!--Mothers' Perceptions and Use of Antibiotics in a Rural Area of Viet Nam. *Trop Doct* 2000;30(1):6-10.doi:https://doi.org/10.1177/004947550003000105

34. Hernandez-Diaz I., Ayala-Melendez A., Gonzalez-Gonzalez E., et al. Knowledge and Beliefs, Behaviors, and Adherence among Latino Parents or Legal Guardians Related to Antibiotic Use for Upper Respiratory Tract Infections in Children under 6 Years of Age. *J Am Pharm Assoc (2003)* 2019;59(4):506-13.doi:https://doi.org/10.1016/j.japh.2019.03.004[published Online First: 20190426]

35. Hika K., Harwood M., Ritchie S., et al. Maori Experiences and Beliefs About Antibiotics and Antimicrobial Resistance for Acute Upper Respiratory Tract Symptoms: A Qualitative Study. *Antibiotics (Basel)* 2022;11(6):714.doi:https://doi.org/10.3390/antibiotics11060714[published Online First: 20220526]

36. Irawati L., Alrasheedy A. A., Hassali M. A., et al. Low-Income Community Knowledge, Attitudes and Perceptions Regarding Antibiotics and Antibiotic Resistance in Jelutong District, Penang, Malaysia: A Qualitative Study. *BMC Public Health* 2019;19(1):1292.doi:https://doi.org/10.1186/s12889-019-7718-9[published Online First: 20191015]

37. Khan F. U., Khan F. U., Hayat K., et al. Knowledge, Attitude and Practices among Consumers toward Antibiotics Use and Antibiotic Resistance in Swat, Khyber-Pakhtunkhwa, Pakistan. *Expert Rev Anti Infect Ther* 2020;18(9):937-46.doi:https://doi.org/10.1080/14787210.2020.1769477[published Online First: 20200609]

38. Kong L. S., Islahudin F., Muthupalaniappen L., et al. Knowledge and Expectations on Antibiotic Use among Older Adults in Malaysia: A Cross-Sectional Survey. *Geriatrics (Basel)* 2019;4(4)doi:https://doi.org/10.3390/geriatrics4040061[published Online First: 20191025]

39. Larson E. L., Dilone J., Garcia M., et al. Factors Which Influence Latino Community Members to Self-Prescribe Antibiotics. *Nurs Res* 2006;55(2):94-102.doi:https://doi.org/10.1097/00006199-200603000-00004

40. Lindenmeyer A., Redwood S., Griffith L., et al. Recent Migrants' Perspectives on Antibiotic Use and Prescribing in Primary Care: A Qualitative Study. *Br J Gen Pract* 2016;66(652):e802-e09.doi:https://doi.org/10.3399/bjgp16X686809[published Online First: 20160830]

41. Mason T., Trochez C., Thomas R., et al. Knowledge and Awareness of the General Public and Perception of Pharmacists About Antibiotic Resistance. *BMC Public Health* 2018;18(1):711.doi:https://doi.org/10.1186/s12889-018-5614-3[published Online First: 20180608]

42. McNulty C. A. M., Collin S. M., Cooper E., et al. Public Understanding and Use of Antibiotics in England: Findings from a Household Survey in 2017. *BMJ Open* 2019;9(10):e030845.doi:https://doi.org/10.1136/bmjopen-2019-030845[published Online First: 20191028]

43. McNulty C., Read B., Quigley A., et al. What the Public in England Know About Antibiotic Use and Resistance in 2020: A Face-to-Face Questionnaire Survey. *BMJ Open* 2022;12(4):e055464.doi:https://doi.org/10.1136/bmjopen-2021-055464[published Online First: 20220406]

44. Norris P., Churchward M., Fa'alau F., et al. Understanding and Use of Antibiotics Amongst Samoan People in New Zealand. *J Prim Health Care* 2009;1(1):30-5.doi:https://www.ncbi.nlm.nih.gov/pubmed/20690484

45. Paredes J. L., Navarro R., Watanabe T., et al. Knowledge, Attitudes and Practices of Parents Towards Antibiotic Use in Rural Communities in Peru: A Cross-Sectional Multicentre Study. *BMC Public Health* 2022;22(1):459.doi:https://doi.org/10.1186/s12889-022-12855-0[published Online First: 20220307]

46. Pattnaik M., Nayak A. K., Karna S., et al. Perception and Determinants Leading to Antimicrobial (Mis)Use: A Knowledge, Attitude, and Practices Study in the Rural Communities of Odisha, India. *Front Public Health* 2022;10(101616579):1074154.doi:https://doi.org/10.3389/fpubh.2022.1074154[published Online First: 20230113]

47. Russom M., Bahta M., Debesai M., et al. Knowledge, Attitude and Practice of Antibiotics and Their Determinants in Eritrea: An Urban Population-Based Survey. *BMJ Open* 2021;11(9):e046432.doi:https://doi.org/10.1136/bmjopen-2020-046432[published Online First: 20210924]

48. Schuts E. C., van Dulm E., Boyd A., et al. Knowledge and Use of Antibiotics in Six Ethnic Groups: The Helius Study. *Antimicrob Resist Infect Control* 2019;8(1):200.doi:https://doi.org/10.1186/s13756-019-0636-x[published Online First: 20191206]

49. Sindato C., Mboera L. E. G., Katale B. Z., et al. Knowledge, Attitudes and Practices Regarding Antimicrobial Use and Resistance among Communities of Ilala, Kilosa and Kibaha Districts of Tanzania. *Antimicrob Resist Infect Control* 2020;9(1)doi:https://doi.org/10.1186/s13756-020-00862-y

50. Ulaya G., Nguyen T. C. T., Vu B. N. T., et al. Awareness of Antibiotics and Antibiotic Resistance in a Rural District of Ha Nam Province, Vietnam: A Cross-Sectional Survey. *Antibiotics (Basel)* 2022;11(12):1751.doi:https://doi.org/10.3390/antibiotics11121751[published Online First: 20221204]

51. Wang N. C. Pre-Visit Use of Non-Prescribed Antibiotics among Child Patients in China: Prevalence, Predictors, and Association with Physicians' Prescribing of Antibiotics at Medical Visits. *Antibiotics (Basel)* 2022;11(11)doi:https://doi.org/10.3390/antibiotics11111553[published Online First: 20221104]

52. Westerling R., Daryani A., Gershuni O., et al. Promoting Rational Antibiotic Use in Turkey and among Turkish Migrants in Europe - Implications of a Qualitative Study in Four Countries. *Global Health* 2020;16(1):108.doi:https://doi.org/10.1186/s12992-020-00637-5[published Online First: 20201111]

53. Whittaker A., Lohm D., Lemoh C., et al. Investigating Understandings of Antibiotics and Antimicrobial Resistance in Diverse Ethnic Communities in Australia: Findings from a

Qualitative Study. *Antibiotics (Basel)* 2019;8(3):135.doi:https://doi.org/10.3390/antibiotics8030135[published Online First: 20190902]

54. Xu Y., Lu J., Sun C., et al. A Cross-Sectional Study of Antibiotic Misuse among Chinese Children in Developed and Less Developed Provinces. *J Infect Dev Ctries* 2020;14(2):129-37.doi:https://doi.org/10.3855/jidc.11938[published Online First: 20200229]

55. Ahiabu M. A., Magnussen P., Bygbjerg I. C., et al. Treatment Practices of Households and Antibiotic Dispensing in Medicine Outlets in Developing Countries: The Case of Ghana. *Res Social Adm Pharm* 2018;14(12):1180-88.doi:https://doi.org/10.1016/j.sapharm.2018.01.013[published Online First: 20180207]

56. Albawani S. M., Bin Hassan Y., Abd-Aziz N., et al. Self-Medication with Antibiotics in Sana'a City, Yemen. *Trop J Pharm Res* 2017;16(5):1195-99.doi:https://doi.org/10.4314/tjpr.v16i5.30

57. Anderson A. Antibiotic Self-Medication and Antibiotic Resistance: Multilevel Regression Analysis of Repeat Cross-Sectional Survey Data in Europe. *Reg* 2021;8(2):121-45.doi:https://doi.org/10.18335/region.v8i2.339

58. Annadurai K., Selvasri S., Ramasamy J. Self Medication: Predictors and Practices among Rural Population of Nellikuppam Village, Kancheepuram District, Tamil Nadu. *J Krishna Inst Med Sci Univ* 2017;6(1):90-98.doi:https://www.jkimsu.com/jkimsu-vol6no1/JKIMSU,%20Vol.%206,%20No.%201,%20Jan-Mar%202017%20Page%2090-98.pdf

59. Ayana H., Sileshi T., Bule M. H., et al. Non-Prescription Antibiotics Use and Associated Factors among Drug Retail Outlets in Ambo, Ethiopia: A Cross-Sectional Study. *Patient Prefer Adherence* 2021;15:2739-47.doi:https://doi.org/10.2147/PPA.S337364[published Online First: 20211210]

60. Barber D. A., Casquejo E., Ybanez P. L., et al. Prevalence and Correlates of Antibiotic Sharing in the Philippines: Antibiotic Misconceptions and Community-Level Access to Non-Medical Sources of Antibiotics. *Trop Med Int Health* 2017;22(5):567-75.doi:https://doi.org/10.1111/tmi.12854[published Online First: 20170309]

61. Chowdhury M., Stewart Williams J., Wertheim H., et al. Rural Community Perceptions of Antibiotic Access and Understanding of Antimicrobial Resistance: Qualitative Evidence from the Health and Demographic Surveillance System Site in Matlab, Bangladesh. *Glob Health Action* 2019;12(sup1):1824383.doi:https://doi.org/10.1080/16549716.2020.1824383

62. Mainous A. G., 3rd, Diaz V. A., Carnemolla M. Factors Affecting Latino Adults' Use of Antibiotics for Self-Medication. *J Am Board Fam Med* 2008;21(2):128-34.doi:https://doi.org/10.3122/jabfm.2008.02.070149

63. Mishra S., Suwannapong N., Tipayamongkholgul M., et al. Access to Health Service and Social Support Related to Self-Medication. *J Nepal Health Res Counc* 2020;18(3):500-05.doi:https://doi.org/10.33314/jnhrc.v18i3.2649[published Online First: 20201114]

64. Nabaweesi I., Olum R., Sekite A. B., et al. Antibiotic Practices, Perceptions and Self-Medication among Patients at a National Referral Hospital in Uganda. *Infect Drug Resist* 2021;14:2155-64.doi:https://doi.org/10.2147/idr.s303075[published Online First: 20210610]

65. Om C., Daily F., Vlieghe E., et al. Pervasive Antibiotic Misuse in the Cambodian Community: Antibiotic-Seeking Behaviour with Unrestricted Access. *Antimicrob Resist Infect Control* 2017;6(1):30.doi:https://doi.org/10.1186/s13756-017-0187-y[published Online First: 20170324]

66. Saradamma R. D., Higginbotham N., Nichter M. Social Factors Influencing the Acquisition of Antibiotics without Prescription in Kerala State, South India. *Soc Sci Med* 2000;50(6):891-903.doi:https://doi.org/10.1016/s0277-9536(99)00380-9

67. Anstey Watkins J., Wagner F., Xavier Gomez-Olive F., et al. Rural South African Community Perceptions of Antibiotic Access and Use: Qualitative Evidence from a Health and Demographic Surveillance System Site. *Am J Trop Med Hyg* 2019;100(6):1378-90.doi:https://doi.org/10.4269/ajtmh.18-0171

68. Do N. T. T., Vu H. T. L., Nguyen C. T. K., et al. Community-Based Antibiotic Access and Use in Six Low-Income and Middle-Income Countries: A Mixed-Method Approach. *Lancet Glob Health* 2021;9(5):e610-e19.doi:https://doi.org/10.1016/S2214-109X(21)00024-3[published Online First: 20210310]

69. Essigmann H. T., Aguilar D. A., Perkison W. B., et al. Epidemiology of Antibiotic Use and Drivers of Cross-Border Procurement in a Mexican American Border Community. *Front Public Health* 2022;10(101616579):832266.doi:https://doi.org/10.3389/fpubh.2022.832266[published Online First: 20220310]

70. Haenssgen M. J., Charoenboon N., Xayavong T., et al. Precarity and Clinical Determinants of Healthcare-Seeking Behaviour and Antibiotic Use in Rural Laos and Thailand. *BMJ Glob Health* 2020;5(12)doi:https://doi.org/10.1136/bmjgh-2020-003779

71. Kamenshchikova A., Wolffs P. F. G., Hoebe C. J., et al. Complex Narratives of Health, Stigma and Control: Antimicrobial Resistance Screening among Non-Hospitalized Refugees. *Soc Sci Med* 2018;212:43-49.doi:https://doi.org/10.1016/j.socscimed.2018.07.012[published Online First: 20180707]

72. Khare S., Pathak A., Purohit M. R., et al. Determinants and Pathways of Healthcare-Seeking Behaviours in under-5 Children for Common Childhood Illnesses and Antibiotic Prescribing: A Cohort Study in Rural India. *BMJ Open* 2021;11(12):e052435.doi:https://doi.org/10.1136/bmjopen-2021-052435[published Online First: 20211203]

73. Kleinert E., Hillermann N., Jablonka A., et al. Prescription of Antibiotics in the Medical Care of Newly Arrived Refugees and Migrants. *Pharmacoepidemiol Drug Saf* 2021;30(8):1074-83.doi:https://doi.org/10.1002/pds.5254[published Online First: 20210504]

74. Lucas P. J., Uddin M. R., Khisa N., et al. Pathways to Antibiotics in Bangladesh: A Qualitative Study Investigating How and When Households Access Medicine Including Antibiotics for Humans or Animals When They Are Ill. *PLoS One* 2019;14(11):e0225270.doi:https://doi.org/10.1371/journal.pone.0225270[published Online First: 20191122]

75. Miller F., Zylbersztejn A., Favarato G., et al. Factors Predicting Amoxicillin Prescribing in Primary Care among Children: A Cohort Study. *Br J Gen Pract* 2022;72(722):e659-67.doi:https://doi.org/10.3399/BJGP.2021.0639[published Online First: 20220404]

76. Rahill G. J., Matthews R., Shelton D. Type and Frequency of Substances Injected in a Sample of Haitian Immigrant Picuristes (Informal Injectionists) and Clients. *J Health Care Poor Underserved* 2012;23(1):114-31.doi:https://doi.org/10.1353/hpu.2012.0018

77. Albanese B. A., Roche J. C., Pass M., et al. Geographic, Demographic, and Seasonal Differences in Penicillin-Resistant Streptococcus Pneumoniae in Baltimore. *Clin Infect Dis* 2002;34(1):15-21.doi:https://doi.org/10.1086/323674[published Online First: 20011120]

78. Angeletti S., Ceccarelli G., Vita S., et al. Unusual Microorganisms and Antimicrobial Resistances in a Group of Syrian Migrants: Sentinel Surveillance Data from an Asylum Seekers Centre in Italy. *Travel Med Infect Dis* 2016;14(2):115-22.doi:https://doi.org/10.1016/j.tmaid.2016.03.005[published Online First: 20160315]

79. Aro T., Kantele A. High Rates of Meticillin-Resistant Staphylococcus Aureus among Asylum Seekers and Refugees Admitted to Helsinki University Hospital, 2010 to 2017. *Euro Surveill* 2018;23(45)doi:https://doi.org/10.2807/1560-7917.ES.2018.23.45.1700797

80. Barger S. D., Lininger M. R., Trotter R. T., 2nd, et al. Educational Attainment and Staphylococcus Aureus Colonization in a Hispanic Border Community: Testing Fundamental Cause Theory. *mSphere* 2020;5(5)doi:https://doi.org/10.1128/mSphere.00623-20[published Online First: 20200930]

81. Charlebois E. D., Bangsberg D. R., Moss N. J., et al. Population-Based Community Prevalence of Methicillin-Resistant Staphylococcus Aureus in the Urban Poor of San Francisco. *Clin Infect Dis* 2002;34(4):425-33.doi:https://doi.org/10.1097/00019048-200202000-00032[published Online First: 20020102]

82. Chattopadhya D., Devi L. S., Grover S. S., et al. Urban Migrant Labourers as Potential Source for Transfer of Antimicrobial Resistance to Rural Community. *J Pure Appl Microbiol* 2020;14(4):2371-81.doi:https://doi.org/10.22207/JPAM.14.4.15

83. Conceicao T., Martins H., Rodrigues S., et al. Staphylococcus Aureus Nasal Carriage among Homeless Population in Lisbon, Portugal. *Eur J Clin Microbiol Infect Dis* 2019;38(11):2037-44.doi:https://doi.org/10.1007/s10096-019-03638-4[published Online First: 20190722]

84. Cooke F. J., Howard J. C., Hugh-Jones C., et al. Meticillin-Resistant Staphylococcus Aureus in the Community: Homeless Are Also at Risk. *J Hosp Infect* 2008;68(2):186-8.doi:https://doi.org/10.1016/j.jhin.2007.11.002[published Online First: 20080114]

85. Dinh A., Saliba M., Saadeh D., et al. Blood Stream Infections Due to Multidrug-Resistant Organisms among Spinal Cord-Injured Patients, Epidemiology over 16 Years and Associated Risks: A Comparative Study. *Spinal Cord* 2016;54(9):720-5.doi:https://doi.org/10.1038/sc.2015.234[published Online First: 20160216]

86. Eiset A. H., Stensvold C. R., Fuursted K., et al. High Prevalence of Methicillin-Resistant Staphylococcus Aureus, Giardia, and Blastocystis in Asymptomatic Syrian Asylum Seekers in Denmark during 2016 through 2018. *J Migr Health* 2020;1-2:100016.doi:https://doi.org/10.1016/j.jmh.2020.100016[published Online First: 20201205]

87. Eshetie S., Unakal C., Gelaw A., et al. Multidrug Resistant and Carbapenemase Producing Enterobacteriaceae among Patients with Urinary Tract Infection at Referral Hospital, Northwest

Ethiopia. *Antimicrob Resist Infect Control* 2015;4(1):12.doi:https://doi.org/10.1186/s13756-015-0054-7[published Online First: 20150417]

88. Farr A. M., Marx M. A., Weiss D., et al. Association of Neighborhood-Level Factors with Hospitalization for Community-Associated Methicillin-Resistant Staphylococcus Aureus, New York City, 2006: A Multilevel Observational Study. *BMC Infect Dis* 2013;13(1):84.doi:https://doi.org/10.1186/1471-2334-13-84[published Online First: 20130213]

89. Galindo G. R., Casey A. J., Yeung A., et al. Community Associated Methicillin Resistant Staphylococcus Aureus among New York City Men Who Have Sex with Men: Qualitative Research Findings and Implications for Public Health Practice. *J Community Health* 2012;37(2):458-67.doi:https://doi.org/10.1007/s10900-011-9463-6

90. Gualandi N., Mu Y., Bamberg W. M., et al. Racial Disparities in Invasive Methicillin-Resistant Staphylococcus Aureus Infections, 2005-2014. *Clin Infect Dis* 2018;67(8):1175-81.doi:https://doi.org/10.1093/cid/ciy277

91. Hewagama S., Spelman T., Einsiedel L. J. Staphylococcus Aureus Bacteraemia at Alice Springs Hospital, Central Australia, 2003-2006. *Intern Med J* 2012;42(5):505-12.doi:https://doi.org/10.1111/j.1445-5994.2011.02449.x

92. Hossain A., Hossain S. A., Fatema A. N., et al. Age and Gender-Specific Antibiotic Resistance Patterns among Bangladeshi Patients with Urinary Tract Infection Caused by Escherichia Coli. *Heliyon* 2020;6(6):e04161.doi:https://doi.org/10.1016/j.heliyon.2020.e04161[published Online First: 20200608]

93. Hota B., Ellenbogen C., Hayden M. K., et al. Community-Associated Methicillin-Resistant Staphylococcus Aureus Skin and Soft Tissue Infections at a Public Hospital: Do Public Housing and Incarceration Amplify Transmission? *Arch Intern Med* 2007;167(10):1026-33.doi:https://doi.org/10.1001/archinte.167.10.1026

94. Immergluck L. C., Leong T., Malhotra K., et al. Geographic Surveillance of Community Associated Mrsa Infections in Children Using Electronic Health Record Data. *BMC Infect Dis* 2019;19(1):170.doi:https://doi.org/10.1186/s12879-019-3682-3[published Online First: 20190218]

95. Intahphuak S., Apidechkul T., Kuipiaphum P. Antibiotic Resistance among the Lahu Hill Tribe People, Northern Thailand: A Cross-Sectional Study. *BMC Infect Dis* 2021;21(1):385.doi:https://doi.org/10.1186/s12879-021-06087-7[published Online First: 20210426]

96. Kurz M. S., Bayingana C., Ndoli J. M., et al. Intense Pre-Admission Carriage and Further Acquisition of Esbl-Producing Enterobacteriaceae among Patients and Their Caregivers in a Tertiary Hospital in Rwanda. *Trop Med Int Health* 2017;22(2):210-20.doi:https://doi.org/10.1111/tmi.12824[published Online First: 20170113]

97. Lambourg E., Siani C., de Preux L. Use of a High-Volume Prescription Database to Explore Health Inequalities in England: Assessing Impacts of Social Deprivation and Temperature on the Prescription Volume of Medicines. *Journal of Public Health-Heidelberg* 2022;30(9):2231-42.doi:https://doi.org/10.1007/s10389-021-01691-y

98. Lanyero H., Eriksen J., Obua C., et al. Use of Antibacterials in the Management of Symptoms of Acute Respiratory Tract Infections among Children under Five Years in Gulu, Northern Uganda: Prevalence and Determinants. *PLoS One* 2020;15(6):e0235164.doi:https://doi.org/10.1371/journal.pone.0235164[published Online First: 20200623]

99. Larramendy S., Gaultier A., Fournier J. P., et al. Local Characteristics Associated with Higher Prevalence of Esbl-Producing Escherichia Coli in Community-Acquired Urinary Tract Infections: An Observational, Cross-Sectional Study. *J Antimicrob Chemother* 2021;76(3):789-95.doi:https://doi.org/10.1093/jac/dkaa514

100. Larson E., Lin S. X., Gomez-Duarte C. Antibiotic Use in Hispanic Households, New York City. *Emerg Infect Dis* 2003;9(9):1096-102.doi:https://doi.org/10.3201/eid0909.020371

101. McMullen K. M., Warren D. K., Woeltje K. F. The Changing Susceptibilities of Methicillin-Resistant Staphylococcus Aureus at a Midwestern Hospital: The Emergence of "Community-Associated" Mrsa. *Am J Infect Control* 2009;37(6):454-7.doi:https://doi.org/10.1016/j.ajic.2008.09.015[published Online First: 20081231]

102. Moremi N., Claus H., Vogel U., et al. Faecal Carriage of Ctx-M Extended-Spectrum Beta-Lactamase-Producing Enterobacteriaceae among Street Children Dwelling in Mwanza City, Tanzania. *PLoS One* 2017;12(9):e0184592.doi:https://doi.org/10.1371/journal.pone.0184592[published Online First: 20170912]

103. Negash A. A., Asrat D., Abebe W., et al. Bacteremic Community-Acquired Pneumonia in Ethiopian Children: Etiology, Antibiotic Resistance, Risk Factors, and Clinical Outcome. *Open Forum Infect Dis* 2019;6(3):ofz029.doi:https://doi.org/10.1093/ofid/ofz029[published Online First: 20190123]

104. Piper Jenks N., Pardos de la Gandara M., D'Orazio B. M., et al. Differences in Prevalence of Community-Associated Mrsa and Mssa among U.S. And Non-U.S. Born Populations in Six New York Community Health Centers. *Travel Med Infect Dis* 2016;14(6):551-60.doi:https://doi.org/10.1016/j.tmaid.2016.10.003[published Online First: 20161020]

105. Quagliarello A. B., Parry C. M., Hien T. T., et al. Factors Associated with Carriage of Penicillin-Resistant Streptococcus Pneumoniae among Vietnamese Children: A Rural-Urban Divide. *J Health Popul Nutr* 2003;21(4):316-24.doi:https://www.ncbi.nlm.nih.gov/pubmed/15038586

106. Ravensbergen S. J., Louka C., Ott A., et al. Proportion of Asylum Seekers Carrying Multi-Drug Resistant Microorganisms Is Persistently Increased after Arrival in the Netherlands. *Antimicrob Resist Infect Control* 2019;8(1):6.doi:https://doi.org/10.1186/s13756-018-0455-5[published Online First: 20190107]

107. Sloth L. B., Nielsen R. T., Ostergaard C., et al. Antibiotic Resistance Patterns of Escherichia Coli in Migrants Vs Non-Migrants: A Study of 14 561 Urine Samples. *J Travel Med* 2019;26(8):taz080.doi:https://doi.org/10.1093/jtm/taz080

108. Stabler S., Paccoud O., Duchesne L., et al. Prevalence of Antimicrobial Resistance and Infectious Diseases in a Hospitalised Migrant Population in Paris, France, a Retrospective Study. *Int J Public Health* 2022;67:1604792.doi:https://doi.org/10.3389/ijph.2022.1604792[published Online First: 20221215]

109. Sutter D. E., Bradshaw L. U., Simkins L. H., et al. High Incidence of Multidrug-Resistant Gram-Negative Bacteria Recovered from Afghan Patients at a Deployed Us Military Hospital. *Infect Control Hosp Epidemiol* 2011;32(9):854-60.doi:https://doi.org/10.1086/661284

110. Tola M. A., Abera N. A., Gebeyehu Y. M., et al. High Prevalence of Extended-Spectrum Beta-Lactamase-Producing Escherichia Coli and Klebsiella Pneumoniae Fecal Carriage among Children under Five Years in Addis Ababa, Ethiopia. *PLoS One* 2021;16(10):e0258117.doi:https://doi.org/10.1371/journal.pone.0258117[published Online First: 20211001]

111. Tornberg-Belanger S. N., Rwigi D., Mugo M., et al. Antimicrobial Resistance Including Extended Spectrum Beta Lactamases (Esbl) among E. Coli Isolated from Kenyan Children at Hospital Discharge. *PLoS Negl Trop Dis* 2022;16(3):e0010283.doi:https://doi.org/10.1371/journal.pntd.0010283[published Online First: 20220331]

112. Williamson D. A., Lim A., Wiles S., et al. Population-Based Incidence and Comparative Demographics of Community-Associated and Healthcare-Associated Escherichia Coli Bloodstream Infection in Auckland, New Zealand, 2005-2011. *BMC Infect Dis* 2013;13(1):385.doi:https://doi.org/10.1186/1471-2334-13-385[published Online First: 20130821]

113. Zoorob R., Grigoryan L., Nash S., et al. Nonprescription Antimicrobial Use in a Primary Care Population in the United States. *Antimicrob Agents Chemother* 2016;60(9):5527-32.doi:https://doi.org/10.1128/AAC.00528-16[published Online First: 20160822]

114. Hood G., Toleikyte L., Ashiru-Oredope D. Assessing National Antimicrobial Resistance Campaigns Using a Health Equity Assessment Tool (Heat). *Antibiotics (Basel)* 2019;8(3):121.doi:https://doi.org/10.3390/antibiotics8030121[published Online First: 20190817]

115. Saito N., Takamura N., Retuerma G. P., et al. Frequent Community Use of Antibiotics among a Low-Economic Status Population in Manila, the Philippines: A Prospective Assessment Using a Urine Antibiotic Bioassay. *Am J Trop Med Hyg* 2018;98(5):1512-19.doi:https://doi.org/10.4269/ajtmh.17-0564[published Online First: 20180301]

116. Cai H. T. N., Tran H. T., Nguyen Y. H. T., et al. Challenges and Lessons Learned in the Development of a Participatory Learning and Action Intervention to Tackle Antibiotic Resistance: Experiences from Northern Vietnam. *Front Public Health* 2022;10:822873.doi:https://doi.org/10.3389/fpubh.2022.822873[published Online First: 20220726]

117. Haenssgen M. J., Xayavong T., Charoenboon N., et al. The Consequences of Amr Education and Awareness Raising: Outputs, Outcomes, and Behavioural Impacts of an Antibiotic-Related Educational Activity in Lao Pdr. *Antibiotics (Basel)* 2018;7(4):95.doi:https://doi.org/10.3390/antibiotics7040095[published Online First: 20181101]

118. Haenssgen M. J., Charoenboon N., Thavethanutthanawin P., et al. Tales of Treatment and New Perspectives for Global Health Research on Antimicrobial Resistance. *Med Humanit* 2021;47(4):e10.doi:https://doi.org/10.1136/medhum-2020-011894[published Online First: 20200918]

119. Jones N., Mitchell J., Cooke P., et al. Gender and Antimicrobial Resistance: What Can We Learn from Applying a Gendered Lens to Data Analysis Using a Participatory Arts Case Study? *Front Glob Womens Health* 2022;3:745862.doi:https://doi.org/10.3389/fgwh.2022.745862[published Online First: 20220527]

120. Leeman-Castillo B. A., Corbett K. K., Aagaard E. M., et al. Acceptability of a Bilingual Interactive Computerized Educational Module in a Poor, Medically Underserved Patient Population. *J Health Commun* 2007;12(1):77-94.doi:https://doi.org/10.1080/10810730601096630

121. Mainous A. G., 3rd, Diaz V. A., Carnemolla M. A Community Intervention to Decrease Antibiotics Used for Self-Medication among Latino Adults. *Ann Fam Med* 2009;7(6):520-6.doi:https://doi.org/10.1370/afm.1061

122. Mitchell J., Cooke P., Ahorlu C., et al. Community Engagement: The Key to Tackling Antimicrobial Resistance (Amr) across a One Health Context? *Glob Public Health* 2022;17(11):2647-64.doi:https://doi.org/10.1080/17441692.2021.2003839[published Online First: 20211209]

123. Munoz G., Mota L., Bowie W. R., et al. Ecosystem Approach to Promoting Appropriate Antibiotic Use for Children in Indigenous Communities in Ecuador. *Rev Panam Salud Publica* 2011;30(6):566-73.doi:https://www.ncbi.nlm.nih.gov/pubmed/22358404

124. Stockwell M. S., Catallozzi M., Meyer D., et al. Improving Care of Upper Respiratory Infections among Latino Early Head Start Parents. *J Immigr Minor Health* 2010;12(6):925-31.doi:https://doi.org/10.1007/s10903-010-9326-8

125. Stockwell M. S., Catallozzi M., Larson E., et al. Effect of a Uri-Related Educational Intervention in Early Head Start on Ed Visits. *Pediatrics* 2014;133(5):e1233-40.doi:https://doi.org/10.1542/peds.2013-2350[published Online First: 20140407]

126. Swe M. M. M., Hlaing P. H., Phyo A. P., et al. Evaluation of the Forum Theatre Approach for Public Engagement around Antibiotic Use in Myanmar. *PLoS One* 2020;15(7):e0235625.doi:https://doi.org/10.1371/journal.pone.0235625[published Online First: 20200709]
